# Genomic basis for an informed conservation management of *Pelophylax* water frogs in Luxembourg

**DOI:** 10.1002/ece3.8810

**Published:** 2022-04-11

**Authors:** Hannah Weigand, Jennifer Cross Lopez de Llergo, Alain C. Frantz

**Affiliations:** ^1^ Musée National d'Histoire Naturelle de Luxembourg Luxembourg City Luxembourg; ^2^ 554853 Fondation faune‐flore Luxembourg City Luxembourg

**Keywords:** ddRAD, hybrid complex, invasive species, polyploidization

## Abstract

Genetic identification methods have become increasingly important for species that are difficult to identify in the field. A case in point is *Pelophylax* water frogs. While their morphological determination is highly complex, they include species protected under EU law and some that are classified as invasive. Additionally, genetic data can provide insights into their complex breeding systems, which may or may not involve the reproductive dependency of one species on another. Here, we generate baseline data for water frog monitoring in Luxembourg. We applied a countrywide sampling approach and used SNPs generated by ddRAD sequencing to identify individuals and infer the breeding systems present in the country. We found *Pelophylax lessonae* and *P*. kl. *esculentus* throughout Luxembourg, mostly living in syntopy. In general, a reproductive dependency of *P*. kl. *esculentus* on *P*. *lessonae* (L‐E system) was revealed. Besides this general system, we detected triploid *P*. kl. *esculentus* in six ponds. This indicates a modified L‐E system with reproductive dependency of the triploids on the diploid *P*. kl. *esculentus*. The invasive *P*. cf. *bedriagae* was detected in three ponds in southern Luxembourg, with evidence for hybridization with native water frogs. In addition to the ddRAD data, we tested a simple genetic method for future monitoring based on the *MND1* marker. It showed in almost all cases, an identical species identification as the ddRAD data and was successfully applied to DNA extracts from mouth swabs. Combining this method with our baseline data will enable informed choices for the protection of native water frog species in Luxembourg.

## Graphical abstract

Information about the hybridogenetic system is important for informed conservation management of water frogs. Hence, we determined species identity and ploidy levels of hundreds of water frogs from Luxembourg using ddRAD sequencing. Our results show that most populations have a L‐E breeding system (*Pelophylax lessonae* and *P*. kl. *esculentus*), with occasional triploid *P*. kl. *esculentus* (LLR) and invasive *P*. cf. *bedriagae*.

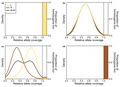

## 1 INTRODUCTION

Genetic identification methods have become increasingly important for species monitoring in recent years (Cordier et al., 2021; Hering et al., 2018). They can be used to determine species that are difficult or impossible to identify in the field (Brodin et al., 2013; Carew & Hoffmann, 2015), and they can compensate for a lack of taxonomic expertise (Hobern, 2021; Yu et al., 2012). In addition, they can be combined with methods that do not require visual encountering, for example, environmental DNA based methods (Biggs et al., 2015; Harper et al., 2019), or minimally invasive sampling methods, such as buccal or skin swabs (Mullin et al., 2021; Ringler, 2018), thus avoiding excessive disturbance of sensitive species.

**FIGURE 1:**
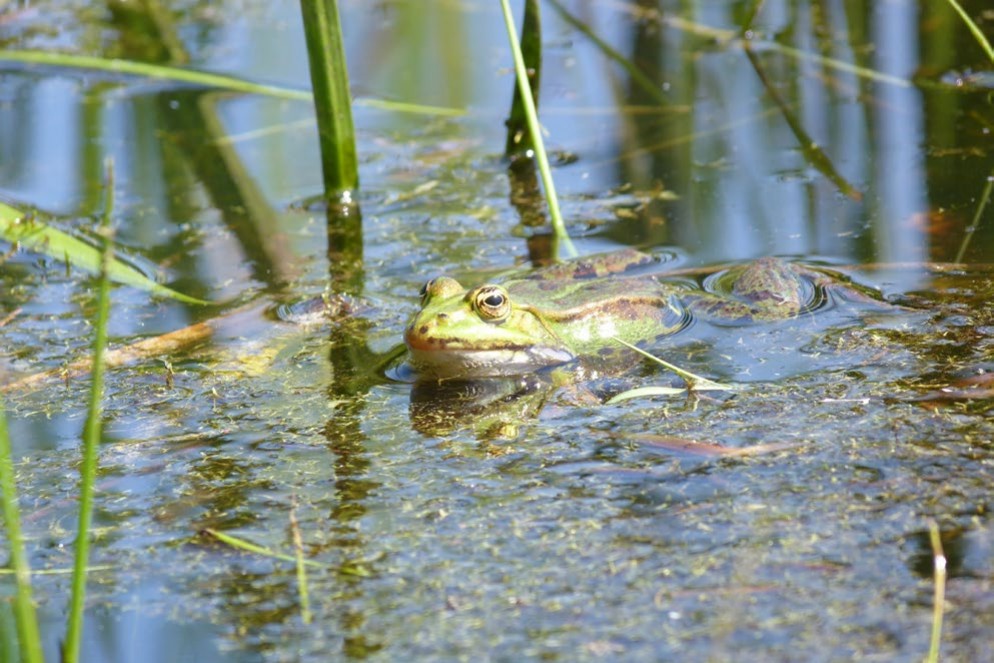
Photograph of a water frog taken in Luxembourg

One genus of interest for the establishment of genetic monitoring is *Pelophylax* water frogs (Figure 1). The three most common native species in Europe are the pool frog *Pelophylax lessonae* (Camerano, 1882), the marsh frog *P*. *ridibundus* (Pallas, 1771), and the edible frog *P*. kl. *esculentus* (Linnaeus, 1758). The three species differ in their ecological requirements (Holenweg Peter, 2001; Negovetic et al., 2001; Pagano et al., 2001) and have a different protection status under the EU Habitats Directive (Council Directive 92/43/EEC). While *P*. *lessonae* is listed in Annex IV and is thus strictly protected within the EU, the other two species are listed in Annex V and member states must ensure that their exploitation and collection in the wild does not have a negative impact on their conservation status. However, morphological determination of these species is highly complex (Plötner, 2010; Tecker et al., 2017), and individuals with intermediate phenotypes cannot be reliably identified (Günther, 1996; Kierzkowski et al., 2011).

Two species of water frogs, *P*. *lessonae* and *P*. kl. *esculentus*, are native to Luxembourg (Schmidt & Proess, 2016). However, as the species are difficult to distinguish during field surveys, knowledge about their abundance and geographical distribution in the country is lacking (Schmidt & Proess, 2016). Hence, they were recorded as a single taxonomic unit in the first and second reporting period of the EU Habitats Directive (Eionet Central Data Repository, 2007, 2013). Also, in addition to the two native water frog species, an invasive *Pelophylax* species, *P*. cf. *bedriagae* (Camerano, 1882), has been reported from the south of Luxembourg (Proess, 2016). This species, which has also been found in the nearby Mosel region in Germany (Ohst, 2008), is currently spreading in Belgium (Holsbeek et al., 2008). Because it is known to threaten native water frogs by competition, predation, or hybridization (Holsbeek et al., 2008; Ohst, 2008; Plötner, 2005), its expansion may negatively impact native Luxembourg water frogs. Genetic methods thus offer a promising tool for determining the composition of Luxembourg water frog populations.

**FIGURE 2:**
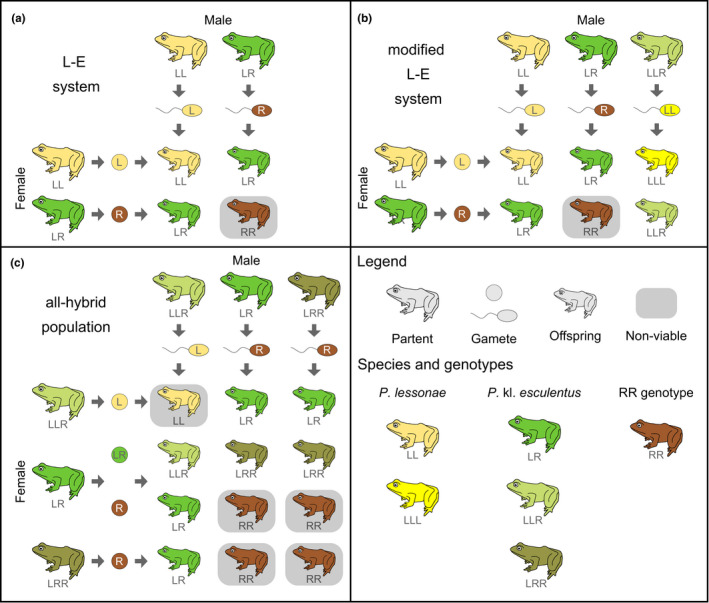
Breeding systems of water frogs that might occur in Luxembourg. (a) L‐E system (following Christiansen, 2009); (b) modified L‐E system (following Pruvost et al., 2013) and (c) all‐hybrid populations (E‐E system; following Christiansen, 2009). The species and their genotypes are color‐coded (see legend). The RR genotype resamples the phenotype of the parental species *P*. *ridibundus* involved in the original hybridization event leading to *P*. kl. *esculentus*. Offspring marked in gray are normally nonviable and do not reach adolescence

Species identification alone may not provide a comprehensive characterization of the diversity of water frogs, as local populations can have different, complex breeding systems. *Pelophylax* kl. *esculentus* is a fertile hybrid (“klepton,” abbreviated kl in nomenclature), resulting from the hybridization of *P*. *lessonae* and *P*. *ridibundus* (Berger, 1967, 1968). Hence, it often requires mating with another water frog species to produce viable offspring. However, the reproduction of *P*. kl. *esculentus* is not dependent on its parental species in all populations (Christiansen, 2009; Holsbeek & Jooris, 2010). Given the two native species *P*. *lessonae* and *P*. kl. *esculentus* (Figure 2), three different breeding systems are possible on a local scale in Luxembourg:

1. The *lessonae*‐*esculentus* system (L‐E system; Figure 2a) can be assumed to be predominant in this geographical region (Holsbeek & Jooris, 2010). Here, *P*. kl. *esculentus* (LR genotype) excludes the L genome from the germline, thus producing only R gametes (Uzzell & Berger, 1975). Interhybrid crosses yield RR offspring that are normally nonviable (Berger & Uzzell, 1977; Binkert et al., 1982), likely due to the accumulation of deleterious mutations (i.e., mutation load; Guex et al., 2002; Vorburger, 2001a). In order to restore the LR genotype and successfully reproduce, *P*. kl. *esculentus* thus needs to backcross with *P*. *lessonae* (LL genotype).
2. The modified L‐E system (Figure 2b) is, in general, similar to the L‐E system but additional triploid *P*. kl. *esculentus* with LLR genotypes are present (Pruvost et al., 2013). These produce an LL gamete. By an interhybrid mating with diploid *P*. kl. *esculentus* (R gamete), LLR offspring arise. As diploid *P*. kl. *esculentus* arise only by backcrossing with *P*. *lessonae*, similar as for the L‐E system, the persistence of diploid and triploid *P*. kl. *esculentus* depends on its parental species.
3. In contrast, the reproduction of *P*. kl. *esculentus* does not depend on *P*. *lessonae* in all‐hybrid populations (E‐E system; Figure 2c; Arioli et al., 2010; Christiansen, 2009; Jakob et al., 2010). Here, diploid and triploid *P*. kl. *esculentus* (with LLR and LRR genotypes) reproduce with each other, resulting in diploid and triploid *P*. kl. *esculentus* offspring. While all‐hybrid populations are mainly found in northwestern Europe (Arioli et al., 2010; Hoffmann et al., 2015; Jakob et al., 2010), they can occur across the geographical range of L‐E systems (Holsbeek & Jooris, 2010).

The monitoring of water frogs thus requires both the identification of the target species found in the local water frog populations and the determination of their ploidy levels and the associated genotypes (i.e., the number of L and R genomes). Several single locus marker (Hauswaldt et al., 2012; Tecker et al., 2017) and microsatellite sets (Christiansen, 2005; Hoffmann et al., 2015) as well as single nucleotide polymorphisms (SNPs) obtained using double digest restriction site‐associated DNA (ddRAD; Dubey et al., 2019) have all been used for species determination and ploidy level inference in water frogs. While technically demanding, the ddRAD approach reduces uncertainties in ploidy level estimation due to the analyses of hundreds to thousands of SNPs.

In the present study, we applied a countrywide sampling approach in combination with ddRAD sequencing to generate baseline data for the management of Luxembourg water frogs. Using thousands of SNPs, we wanted to (1) determine species identity of the sampled water frogs, (2) understand the geographic distribution and abundance of both native species as well as the invasive *P*. cf. *bedriagae*, (3) determine the ploidy level (genotype) of the sampled individuals and (4) map the geographic distribution of diploid and triploid *P*. kl. *esculentus*. Finally, in order to develop a simple method for routine monitoring, we also (5) performed species identification based on a single locus marker (*MND1*, Tecker et al., 2017) and different DNA source materials (toe clips, buccal and skin swab) and compared the results to ddRAD‐based species identification.

## 2 MATERIALS AND METHODS

### 2.1 Ethics approval

Frogs were captured under a permit issued by the Ministry of the Environment, Climate and Sustainable Development Luxembourg (90977 CD/NE).

### 2.2 Sampling

**FIGURE 3:**
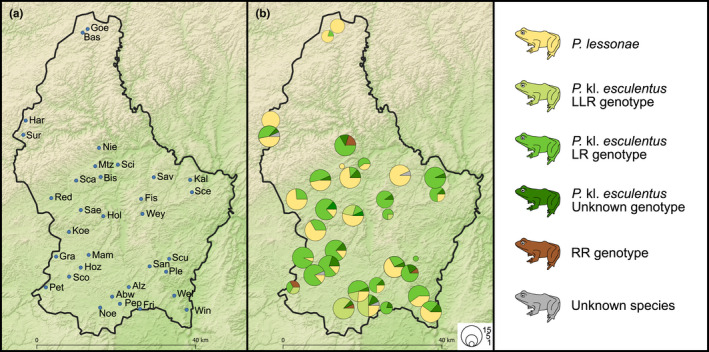
Map of Luxembourg showing the distribution of water frogs. (a) Sample sites. (b) Water frog species and genotypes. The size of the pie charts is scaled according to the number of analyzed specimens

We sampled 382 frogs from 33 ponds distributed across Luxembourg (Figure 3a; Table S1). Individuals were hand‐caught at night, and tissue samples (toe clips) were taken to obtain sufficient high‐quality DNA for ddRAD sequencing following DECC (2008). The third toe on the right forefoot was first disinfected with 1% iso‐Betadine dermicum (Meda Manufacturing, France) and locally anesthetized with a 2% Xylocaine gel (AstraZeneca, Germany). Then, the foremost toe fragment was removed with flame‐sterilized scissors. The wound was disinfected with iso‐Betadine and closed with Vetbond (3M Animal Care Products). Toe fragments were stored in 96% ethanol. In addition, we used Isohelix buccal swabs (Westburg, the Netherlands) to sample buccal and skin cells from 20 frogs. Swabs were put in 96% ethanol or kept dry. All samples were stored at −20°C until DNA extraction. Material that came into contact with pond water and/or water frogs was either discarded or disinfected with 1% Virkon S (DuPont).

### 2.3 DNA extraction

DNA was extracted from toe fragments and swabs using an ammonium‐acetate‐based salting‐out procedure (modified according to Richardson et al., 2001, Text S1).

### 2.4 ddRAD sequencing

DNA from toe fragments was used for ddRAD libraries preparation (detailed protocol in Text S2). Briefly, the DNA was digested with the restriction enzymes *MspI* (C|CGG) and *SbfI* (CCTGCA|GG; both New England Biolabs). Subsequently, the first parts of the sequencing adapters (Text S3) were ligated to the generated fragments using T4 DNA Ligase (New England Biolabs). Among others, the sequencing adapters contained a degenerated base region (DBR) for PCR duplicate detection (Schweyen et al., 2014). Size selection was performed with SPRIselect (Beckman Coulter) to eliminate small DNA fragments (e.g., sequencing adapter dimers) by using a SPRIselect‐to‐DNA ratio of 1:1. In the subsequent PCR with the Q5 High‐Fidelity DNA Polymerase (New England Biolabs), the remaining part of the sequencing adapters were added, including two index barcodes for specimen identification. Next, double size selection with SPRIselect (SPRIselect‐to‐DNA ratio of 0.7:1 and 0.85:1) was applied to retain fragments of approx. 200 to 550 bp. The ddRAD library preparation was repeated once or twice for 73 samples (Tables S2 and S3). Finally, the samples were pooled into four libraries that were each sequenced at the Luxembourg Centre for Systems Biomedicine, University of Luxembourg, with a NextSeq 500 (Illumina) High Output Flow Cell.

### 2.5 ddRAD analysis

First, a quality control of raw ddRAD reads was carried out with trim_galore (www.bioinformatics.babraham.ac.uk/projects/trim_galore). In this step, reads that were too short or had a low sequencing quality were removed. Subsequently, PCR duplicates were identified and excluded using a custom‐written script (all custom‐written scripts are deposited at https://github.com/haweigand/ddRAD_Pelophylax) following Schweyen et al. (2014). Afterward, loci were identified using Stacks v2 (Catchen et al., 2013).

A parameter test was run to evaluate the performance of different Stacks settings. We used different values for the minimal number of identical reads needed to build a stack (*m*: 3,5,7), the maximum distance allowed between stacks of the same locus and individual (*M*: 3,5,7) and the maximum distance allowed to align secondary reads to primary stacks (*n* = *M*). The parameter test was based only on replicated individuals and from these only the samples that had a minimum of 100,000 reads (39 individuals with two and two with three replicates). The data were filtered to include only SNPs with a minimum coverage of eight reads and a minor allele frequency of 0.05. The maximum percentage of missing data per SNP (mds: 30%, 40%, 50%) and per individual (mdi: 30%, 40%, 50%) were tested as filtering options (custom‐written script). The different Stacks settings and filtering options were evaluated by examining the number of loci and individuals included in the dataset and the proportion of missing data. Additionally, a SNP‐calling error rate was calculated by identifying the proportion of SNPs that were differentially called between the replicated individuals (custom‐written script). The final Stacks settings and filtering options were chosen to maximize the number of individuals and loci included, while having a low SNP‐calling error rate.

Next, in case of replicated individuals, either the replicate with the most reads was selected (>300,000 reads) or the reads from the replicates were combined. All individuals with more than 50,000 reads were used in the subsequent Stacks analysis. A high‐quality dataset was obtained by applying the Stacks settings and filtering options chosen from the parameter test.

### 2.6 Species identification and ploidy level inference

As the specimens were not identified morphologically, a priori information about species identity was missing. Hence, we used two methods to identify genetic clusters in the high‐quality dataset that could be assigned to species and ploidy level later on. First, we performed a principal component analysis (PCA) using the R package Lea v.2.0 (Frichot & François, 2015).

Second, we used a sparse non‐negative matrix factorization (sNMF; Frichot et al., 2014) with the R package Lea. In this clustering method, the admixture coefficients of each sample were inferred for one to ten ancestral populations (*K*). We performed ten independent replicates for each value of *K* and retained the replicate with lowest cross‐entropy value. Next, the number of clusters in the dataset was chosen by taking the value of *K* for which the cross‐entropy curve showed a plateau. If an individual's ancestry coefficient was *q *> 0.8 for this *K*, the corresponding cluster was assumed to be its population of origin.

In the sNMF method, the ploidy level of the dataset has to be defined a priori. Given the potential presence of diploids and triploids, we run the analysis one time setting ploidy to two and one time setting it to three.

With the identified clusters based on PCA and sNMF analysis, we analyzed the genomic composition of each individual. This enabled us to assign species name and ploidy levels to the genetic clusters. To this end, we assumed that specific alleles of certain diagnostic loci should be fixed in the L and R genomes, respectively. *Pelophylax lessonae* should thus be homozygous for one allele and members of the *P*. *ridibundus* complex (if present) homozygous for the alternative allele, while *P*. kl. *esculentus* should be heterozygous. We estimate ploidy level based on a dosage effect. For diploid *P*. kl. *esculentus*, we assumed that the majority of the loci should have equal read coverages for both alleles. In contrast, triploid individuals should have several loci with relative read coverages of 1/3 or 2/3 for the R and L alleles.

In order to identify these diagnostic loci, we assumed that the two largest clusters in the PCA represent *P*. *lessonae* and diploid *P*. kl. *esculentus*. From these two clusters, ten samples each were selected that were centrally located in the clusters and had genotype information for a high percentage of loci. Next, we identified loci that were homozygous for all ten individuals of one cluster and heterozygous for all ten individuals of the other cluster and vice versa. Per cluster and diagnostic locus, we tolerated three samples with missing data. Only one of the two assignments of the PCA clusters to *P*. *lessonae* and diploid *P*. kl. *esculentus* resulted in several diagnostic loci. Hence, this assignment was used to determine which of the alleles of the diagnostic loci originated from the L and which from the R genome.

For each individual in the high‐quality dataset, we estimated the proportion of homozygous diagnostic loci and the read coverage per allele for heterozygous diagnostic loci. The results were visualized using a custom‐written script.

Several individuals were lacking from the high‐quality dataset due to high proportions of missing data. We filtered their SNP genotypes for the diagnostic loci defined above and performed species and ploidy level identification using the genomic composition analysis.

### 2.7 Single marker analysis

In addition to ddRAD data, we wanted to test a simple genetic marker for species identification, that could be used in future water frog monitoring in Luxembourg. Mitochondrial markers such as 16S or COI, that are commonly applied for species identification, do not differentiate between *P*. *lessonae* and *P*. kl. *esculentus*, as they share their mitochondrial linage. Hence, we tested the nuclear marker *MND1* (meiotic nuclear division gene 1; Tecker et al., 2017), which was successfully applied for species identification in a nearby region (North Rhine‐Westphalia, Germany). Because the R and L alleles differ by two nucleotide substitutions (Tecker et al., 2017), we sequenced a 33 bp‐long fragment of the *MND1* locus following Tecker et al. (2017) with modifications (Text S4). Sequences were aligned using Muscle (Edgar, 2004) in Geneious v.5 (www.geneious.com). Sequencing was performed using DNA extracts from toe clips. In addition, 20 samples each were analyzed with DNA extracts from buccal and skin swabs.

There are different explanations for individuals with RR genotypes in the ddRAD data. First, they could represent *P*. *ridibundus* s.s. Although not native to Luxembourg, the species was found in nearby regions and seems to expand its range in the last decades (Holsbeek & Jooris, 2010; Holsbeek et al., 2008). Alternatively, *P*. cf. *bedriagae* as member of the *P*. *ridibundus* species complex has an RR genotype, too. Furthermore, backcrosses of *P*. kl. *esculentus* have RR genotypes, though they are normally nonviable. To discriminate between these cases, we analyzed the mitochondrial lineages of individuals with RR genotype using the *ND1* marker (NADH dehydrogenase subunit 1; Holsbeek et al., 2008). Additionally, other individuals from ponds with RR genotypes as well as individuals from ponds with triploid *P*. kl. *esculentus* were analyzed. A 574‐bp‐long fragment of the marker was sequenced from toe clip DNA following Holsbeek et al. (2008) with modifications (Text S4). Sequences were aligned with Muscle in Geneious. Reference sequences for *P*. *lessonae* [EU835584], *P*. *ridibundus* [EU835583] and *P*. cf. *bedriagae* [EU835605] from Holsbeek et al. (2008) were added. A minimum spanning network (Bandelt et al., 1999) was generated using popart v.1.7 (www.popart.otago.ac.nz).

## 3 RESULTS

### 3.1 ddRAD data

The sequencing of four ddRAD libraries generated a total of 1,890,485,794 reads (min.: library1 – 439,074,203; max.: library3 – 506,137,511). After stringent quality control, between 64,747 und 2,079,656 reads per sample (average: 484,346 reads; Table S3) were used for further analysis. Two out of the 382 individuals generated too few reads for subsequent analysis (Sav06 und Sco06).

In the parameter test for Stacks settings and subsequent filtering options, 88 replicates from 43 individuals were included. The number of SNPs per dataset ranged from 671 (*m*: 7; *M*: 3; mds: 30%; mdi: 30%; Table S4) to 3434 (*m*: 3; *M*: 7; mds: 50%, mdi: 40%) and the number of samples from 41 (*m*: 7; *M*: 5; mds: 50%, mdi: 30%) to 83 (*m*: 7; *M*: 3 and 5; mds: 30%, mdi: 50%). The proportion of SNP calling errors ranged from 1.51% (*m*: 3; *M*: 3; mds: 50%, mdi: 30%) to 2.53% (*m*: 7; *M*: 7; mds: 40%, mdi: 30%; Table S4). As final setting *m*: 3, *M*: 3, mds: 30%, mdi: 30% was chosen, having overall a low error rate (1.56%) with an intermediate number of SNPs (1020) and samples (*N *= 67, Table S4). Using these parameters, we generated a high‐quality dataset that contained *N* = 292 samples with 1081 SNPs.

### 3.2 Species identification and ploidy level inference

**FIGURE 4:**
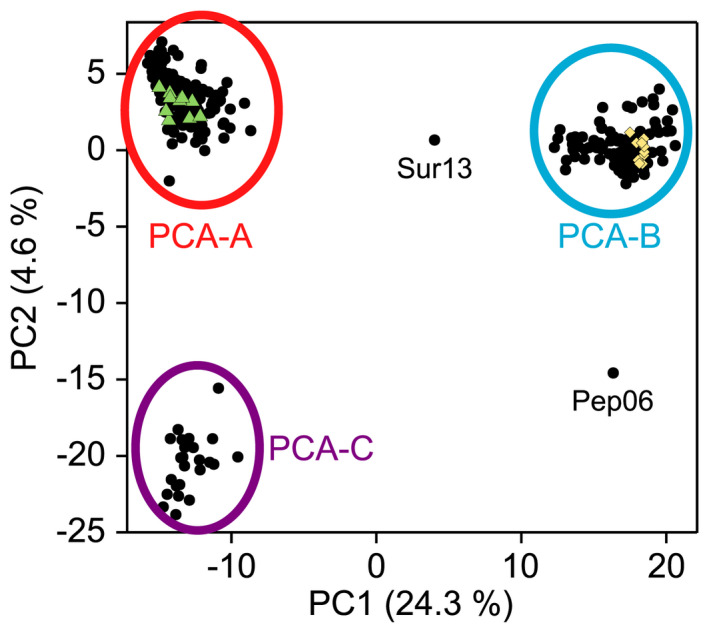
Principal component analysis. Clusters are marked with circles. Individuals used as reference for the genomic composition analysis are highlighted in yellow (*P*. *lessonae*) and green (*P*. kl. *esculentus*)

In the PCA, the first axis explained 24.3% of the variance in the data, while the second explained 4.6% (Figure 4). We identified three clearly distinct clusters. Clusters PCA‐A (140 samples) and PCA‐C (24 samples) were separated from PCA‐B (126 samples) along the first axis. Cluster PCA‐C was located in the same range as PCA‐A on the first axis, but separated from PCA‐A along the second axis. We detected two outliers: Sur13 was located between clusters PCA‐A and PCA‐B, while Pep06 was located on the same level as PCA‐B at the first PCA axis and as PCA‐C on the second axis.

**FIGURE 5:**
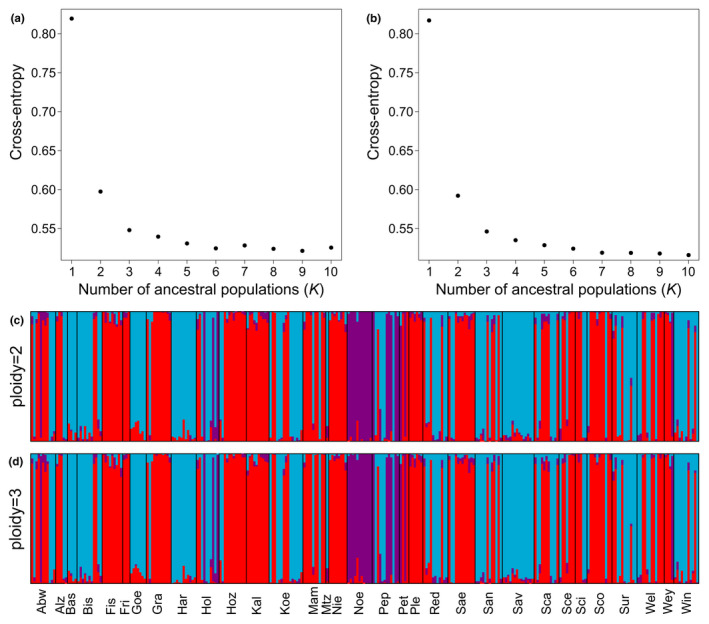
sNMF analysis. (a, b) Cross‐entropy values for *K* ranging from one to ten. The replicate with the lowest cross‐entropy per *K*‐value is shown. Ploidy was set to two (a) or three (b). (c, d) sNMF barplots show the ancestry coefficient (population origin) per individuals for *K *= 3. Ploidy was set to two (c) or three (d). The three clusters corresponded to the three PCA clusters A, B and C (Figure 4)

In the sNMF analysis, the clustering and assignment results obtained for the different ploidy levels were essentially identical (Figure 5, Table S2). In both cases, the largest decrease of cross‐entropy was seen for *K *= 3 with no further strong decrease for increasing values of *K* (Figure 5a,b). Hence, we used *K *= 3 for cluster assignment. The three clusters corresponded to the three PCA clusters A, B, and C (Figures 4 and 5, same color). As in the PCA, frog Sur13 had no clear population origin, but the admixture coefficients of the equivalent of PCA cluster A (0.45) and B (0.54) had both high proportions (Figure 5c,d, Table S2). Another three samples (Sur01, Noe07, Pep06) did not allow a clear assignment to a single population, as they had admixture coefficients of *q* < 0.8 for both tested ploidy value settings (Figure 5c,d, Table S2). From these samples, Sur01 and Noe07 had relatively high proportions of missing data (approx. 28%).

A set of 176 diagnostic SNPs for the L and R genomes was identified for the genomic composition analysis using the reference individuals from PCA‐A and PCA‐B (Figure 4).

**FIGURE 6:**
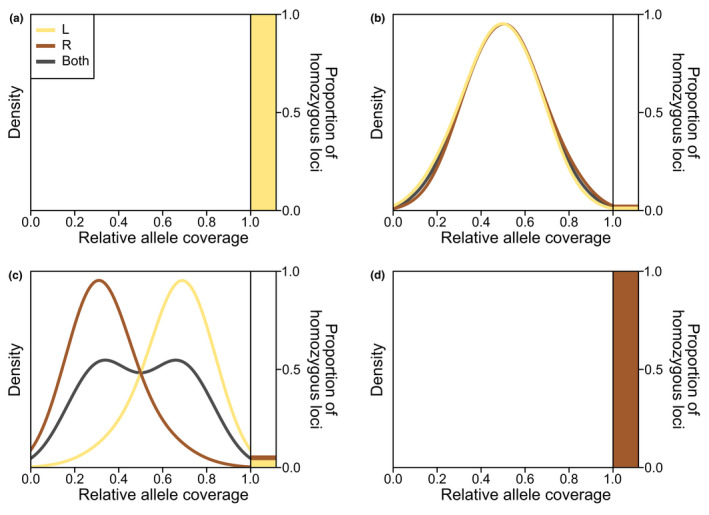
Examples for the genomic composition analysis. The left part of each plot shows for the heterozygous loci the density distribution of the relative coverage for the L and R allele as well as for both alleles together. The right part of the plots indicates the proportion of homozygous loci fixed for the L and R allele. (a) *P*. *lessonae* (LL): Bis04; (b) diploid *P*. kl. *esculentus* (LR): Gra11; (c) triploid *P*. kl. *esculentus* (LLR): Abw06; (d) RR genotype: Nie09

With these diagnostic SNPs, it was possible to differentiate between the different species and ploidy levels (Figure 6, Figure S1). *Pelophylax lessonae* (Figure 6a) corresponded to PCA‐B (Figure 4), diploid *P*. kl. *esculentus* (Figure 6b) corresponded to PCA‐A (Figure 4), while triploid *P*. kl. *esculentus* (Figure 6c) corresponded to PCA‐C (Figure 4). Almost all individuals could be clearly identified by the genomic composition analysis. One exception was Sur13, for which 56% of the diagnostic loci were fixed for the *P*. *lessonae* genome, while the remaining ones showed a distribution typical for diploid *P*. kl. *esculentus* (Figure S1, Table S2). Additionally, 18 samples could only be determined to be *P*. kl. *esculentus*, as the variable loci did not show a clear distribution pattern. Overall, species identification was possible for 290 of the 292 samples of the high‐quality dataset by two of the three methods (Table S2): 126 *P*. *lessonae* (126), 139 diploid *P*. kl. *esculentus*, 24 triploid *P*. kl. *esculentus* and one *P*. kl. *esculentus* with unclear ploidy (Sur01). Only Sur13 and Pep06 could not be determined.

The 88 samples, that were not included in the high‐quality dataset, were identified using the genomic composition analysis. In addition to the previously described patterns, seven individuals showed a high proportion of diagnostic loci with homozygous R alleles. This indicates a potential *P*. *ridibundus* complex identification (Figure 6d, Figure S1). Additionally, 13 individuals were identified as *P*. *lessonae*, 34 as diploid *P*. kl. *esculentus* and seven as triploid *P*. kl. *esculentus*. Twenty‐seven individuals were identified as *P*. kl. *esculentus*, but the ploidy level could not be determined.

Out of 380 individuals with ddRAD data, 139 were identified as *P*. *lessonae*, 232 as *P*. kl. *esculentus* and seven as having a RR genotype, with two individuals (Sur13, Pep06) not being determined (Figure 3; Tables [Link], [Link] and [Link], [Link]). Of all *P*. kl. *esculentus*, 173 were diploid and 31 triploid, and in 28 cases, the ploidy level remained undetermined. *Pelophylax lessonae* and *P*. kl. *esculentus* were found throughout Luxembourg and occurred frequently (20 out of 33 ponds, Figure 3b) in syntopy (i.e., in the same ponds). In contrast, specimens with RR genotypes were only found in four ponds in south and central Luxembourg, always in syntopy with *P*. kl. *esculentus* but never with *P*. *lessonae*. Triploid *P*. kl. *esculentus* were found in six ponds located in south and central Luxembourg (Figure 3, Table S1). *P*. *lessonae* occurred in three of them, while individuals with RR genotypes were found in two of the others.

### 3.3 Mitochondrial lineage identification

**FIGURE 7:**
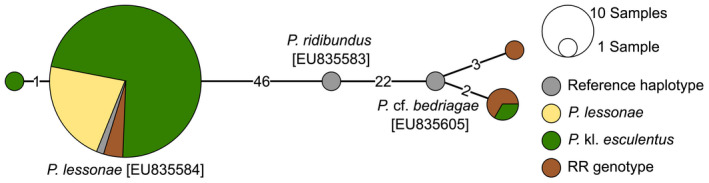
Haplotype network based on the mitochondrial *ND1* fragment. Reference sequences with GenBank accessions are labelled

A 336‐bp‐long fragment of the *ND1* marker was sequenced successfully for six of the seven samples with an RR genotype as well as for 71 additional individuals. Three haplotype groups were identified that were separated by several mutational steps (Figure 7). The haplotype group that included the *P*. *lessonae* reference sequence contained all *P*. *lessonae* and all but one *P*. kl. *esculentus* sequence. Additionally, the three individuals with RR genotype from Nie were assigned to this haplotype group. The haplotype group that included the *P*. cf. *bedriagae* reference sequence contained the remaining three individuals with RR genotype as well as one individual with a *P*. kl. *esculentus* genotype (Pet01). No individual was associated with the *P*. *ridibundus* reference sequence.

### 3.4 Single locus markers for species identification

Using DNA extracted from toe clips, we generated 33‐bp‐long *MND1* sequences that allowed a unique species identification for 366 out of the 382 individuals (Figure S2). For 349 individuals, it matched the ddRAD species identification, while 14 samples did not show a coherent result (Table 1). Although the overall success was high, three out of seven individuals with RR genotype were misidentified as *P*. kl. *esculentus* (one clustering in the *P*. *lessonae* haplogroup, one in the *P*. cf. *bedriagae* haplogroup).

**TABLE 1:** Comparison of species identification using ddRAD and *MND1*

		ddRAD			
		*P. lessonae*	*P*. kl. *esculentus*	RR genotype	?
*MND1*	*P. lessonae*	130	8	0	0
	*P*. kl. *esculentus*	3	215	3	3
	RR genotype	0	0	4	0
	?	7	9	0	0

Note

DNA extracts from toe clips were used as starting material for the *MND1* analysis.? = Could not be identified or individuals with missing data.

*MND1* sequences based on DNA extracts from buccal swabs were consisted with ddRAD species identification for all 20 samples (Table 2, Figure S3). In contrast, *MND1* sequences based on DNA from skin swabs identified 12 individuals as *P*. *lessonae* that were either as *P*. kl. *esculentus* or as having an RR genotype by ddRAD (Table 2, Figure S3).

**TABLE 2:** Comparison of species identification using ddRAD and *MND1*

			ddRAD		
			*P. lessonae*	*P*. kl. *esculentus*	RR genotype
*MND1*	Buccal swabs	*P. lessonae*	6	0	0
		*P*. kl. *esculentus*	0	13	0
		RR genotype	0	0	1
	Skin swabs	*P. lessonae*	6	11	1
		*P*. kl. *esculentus*	0	2	0
		RR genotype	0	0	0

Note

DNA extracts from buccal or skin swabs were used as starting material for the *MND1* analysis.

## 4 DISCUSSION

### 4.1 Species identification of Luxembourg water frogs

In the present study, we were able to determine almost all sampled individuals at the species level and thus can provide a realistic assessment of prevalence and distribution of water frog species in Luxembourg. As assumed by Schmidt and Proess (2016), we found *P*. *lessonae* and *P*. kl. *esculentus* to occur predominantly in the country. Additionally, we identified seven individuals with an RR genotype. Based on the mitochondrial *ND1* marker, three of these were identified as *P*. cf. *bedriagae*. This invasive species was previously reported from southern Luxembourg (Proess, 2016), where it was also found in the current study. As a *P*. cf. *bedriagae* haplotype was found in one *P*. kl. *esculentus* individual, hybridization among the native and invasive water frog species appears to be taking place. In contrast to the individuals from the southern populations, the three individuals from Nie with RR genotypes clustered in the *P*. *lessonae*/*P*. kl. *esculentus* haplotype group of *ND1*. Hence, rather than being the offspring of *P*. cf. *bedriagae* parents, we hypothesize that they originated from mating of two *P*. kl. *esculentus* parents. RR individuals resulting from these mating are usually not viable and die before adolescence (Berger, 1967; Guex et al., 2002; Vorburger, 2001b). Nevertheless, RR individuals originating from *P*. kl. *esculentus* were also found in two populations in Switzerland (Dubey et al., 2019; Vorburger, 2001a). As this phenomenon was only found in one pond in Luxembourg, but with three individuals, this population might represent a special situation in which RR offspring have a higher viability compared to other Luxembourgish populations.

Two samples could not be identified unambiguously. First, Sur13 showed an intermixture among *P*. *lessonae* and diploid *P*. kl. *esculentus* for all three methods. Hence, we assume that the sample was contaminated with the DNA of the second species. Second, while Pep06 showed ambiguous results, much is in favor for this individual being *P*. *lessonae*: (i) in the genomic composition method a clear *P*. *lessonae* origin was found (ii) although ambiguous in the sNMF analysis, the *P*. *lessonae* cluster had values above 0.7 (iii) on the first PCA axis, the sample was found at the same level as the other *P*. *lessonae* individuals.

### 4.2 Population system of water frogs in Luxembourg

*Pelophylax lessonae* and *P*. kl. *esculentus* occurred in syntopy in the majority of ponds and most of the *P*. kl. *esculentus* individuals were diploid, corroborating the expectations of Holsbeek and Jooris (2010) that an L‐E system can be inferred as the primary breeding system in Luxembourg. Still, there are two exceptions to this general assumption: *Pelophylax* kl. *esculentus* occurred in nine ponds without proven presence of *P*. *lessonae*, and triploid *P*. kl. *esculentus* (all LLR genotypes) were detected in six ponds. Both findings imply a more complex breeding system. One potential explanation for the finding of triploid *P*. kl. *esculentus* and the absence of *P*. *lessonae* are all‐hybrid populations. In this case, LRR individuals are usually found alongside LR and LLR individuals (Arioli et al., 2010; Jakob et al., 2010). However, we did not detect any LRR individuals in the present study.

Alternatively, the LLR individuals could indicate a modified L‐E system in which LRR individuals are absent. This is supported by the PCA, in which all LLR individuals formed their own cluster. For all‐hybrid populations, both genomes have been found to "travel" between the different genotypes (Arioli et al., 2010). Therefore, we would expect to find no clear separation of LR, LRR and LLR individuals in a PCA. In contrast, in a modified LE system, both L genomes from the LLR individual are passed clonally to the new LLR progeny, while only the R is derived from the diploid *P*. kl. *esculentus* individual (Pruvost et al., 2013). This makes a separate cluster with all LLR individuals likely. Also, in a modified L‐E system, LLL individuals might result from the mating of *P*. *lessonae* with an LLR individual. An LLL genotype might explain the unclear species identification of Pep06. First, in the PCA, it clustered with the *P*. *lessonae* group along the first axis and with the LLR group along the second axis. Second, in the sNMF analysis at Pep06 shared approx. 25% of its ancestry with the LLR cluster. In the genomic composition analysis, an LLL individuals should not be distinguishable from a diploid *P*. *lessonae*, as all loci should be homozygous for the L‐allele. This pattern was also found for Pep06.

Rather than being indicative of all‐hybrid populations, the absence of *P*. *lessonae* from some ponds with *P*. kl. *esculentus* could result from a sampling artifact. For example, unfavorable ecological conditions for *P*. *lessonae* may have resulted in low abundance compared to *P*. kl. *esculentus* (see Mikulíček et al., 2015; Pagano et al., 2001), or, alternatively, not all sampled populations represent breeding sites (e.g., Dubey et al., 2019).

When taken together, several indications suggest a modified L‐E system rather than all‐hybrid populations. However, the low frequency of LLR individuals in the total dataset and the limited number of samples per population permits only a presumption about the origin of the triploids. The hypothesis could be validated by analyzing the genomic composition of the gametes, since in the modified L‐E system LLR individuals are assumed to produce LL gametes and LR individuals R gametes (Mikulíček et al., 2015; Mikulíček & Kotlík, 2001; Pruvost et al., 2013), while in all‐hybrid populations, LLR individuals are assumed to produce L gametes and LR individuals LR and R gametes (Christiansen, 2009; Günther et al., 1979).

### 4.3 Recommendations for future monitoring

Our findings on the species and breeding system have different implications for water frog management in Luxembourg. First, in the two most likely occurring breeding systems, the L‐E system and the modified L‐E system, the persistence of *P*. kl. *esculentus* depends on its mating with *P*. *lessonae*. Hence, ponds must present conditions suitable for this species, which has also the higher protection status (Council Directive 92/43/EEC).

Second, the modified L‐E system seems to occur currently only in few ponds (triploid found in six ponds only) in central and south Luxembourg. Hence, these ponds should be priories in water frog management. Furthermore, applying ddRAD sequencing again in a few years’ time could show if the modified L‐E system is spreading or limited to its current populations. This might enable further ecological studies analyzing the evolutionary trade‐offs of this system.

Third, we found three individuals with RR genotype, most likely origination from *P*. kl. *esculentus* mating. As all were found in one pond at Niederfeulen (Nie), and this seems to be a rare event (see Vorburger, 2001a), we advocate to give a high priority in water frog conservation to this population.

Finally, *P*. cf. *bedriagae* was found in three ponds in the south of Luxembourg. As it is known to threaten native water frogs by competition, predation, or hybridization (Holsbeek et al., 2008; Ohst, 2008; Plötner, 2005), its expansion should be monitored in the future. This might be even more pressing, as we have already observed one case of hybridization.

To enable a future easy and inexpensive monitoring of water frogs in Luxembourg, we tested species identification with the *MND1* marker (Tecker et al., 2017). While the marker had in general a high identification rate of 96%, three out of seven individuals with RR genotype were misidentified as *P*. kl. *esculentus*. Hence, we recommend its combination with *ND1*. Although this may not prevent misidentifications in the case of RR individuals with *P*. kl. *esculentus* origin, individuals of the invasive species *P*. cf. *bedriagae* should be clearly identifiable.

For ethical, but also practical reasons, invasive sampling methods such as toe clipping should be reduced to a minimum. Hence, we tested skin and buccal swabs as potential source material for DNA extraction in genetic monitoring. Skin swabs did not lead to a sufficient amount of high‐quality DNA and thus to misidentifications due to nonamplified alleles. In contrast, *MND1* analyses from buccal swabs performed very well, so that buccal swabs can be recommended for the future.

A noninvasive, rapid, and cost‐effective screening for *P*. cf. *bedriagae* could be done in the future by the application of aquatic eDNA analyses based on the *ND1* gene. This could be achieved by either designing species‐specific primers (e.g., as for *P*. *lessonae* by Eiler et al., 2018) or by using a metabarcoding approach (see Ficetola et al., 2019).

In summary, the application of a country‐wide sampling in combination with ddRAD sequencing provided a profound insight into the complex population systems of Luxembourg's water frogs. As it might be too expensive and labor‐intensive for routine monitoring, buccal swabs in combination with *MND1*‐ and *ND1*‐sequencing showed a high ability for correct species identification.

Still, repeating the current approach within a few years’ time might enable a deeper understanding of the ongoing population processes, such as a potential spread of the modified L‐E system, Furthermore, it might generate insights into the impact of *P*. cf. *bedriagae* as invasive species on the genetic diversity of native water frog populations.

## CONFLICT OF INTEREST

None.

## AUTHOR CONTRIBUTIONS

**Hannah Weigand:** Conceptualization (equal); Data curation (lead); Formal analysis (lead); Funding acquisition (equal); Investigation (lead); Visualization (lead); Writing – original draft (lead); Writing – review & editing (equal). **Jennifer Cross Lopez de Llergo:** Investigation (supporting); Writing – review & editing (supporting). **Alain C. Frantz:** Conceptualization (equal); Funding acquisition (equal); Resources (lead); Writing – original draft (supporting); Writing – review & editing (equal).

## Supporting information

Figure S1

Figure S2

Figure S3

Table S1

Table S2

Table S3

Table S4

Text S1

Text S2

Text S3

Text S4

## Data Availability

*ddRAD data:* NCBI BioProject: PRJNA678837; NCBI SRA: SRR13073509–SRR13073602; SRR13123976–SRR13124078; SRR13154636–SRR13154733; SRR13189070–SRR13189173. *ND1* sequences: NCBI GenBank: MZ457241–MZ457319.
