# Supplementary material for: Genomic basis for an informed conservation management of Pelophylax water frogs in Luxembourg
Source: Ecol Evol. 2022 Apr 11;12(4):e8810. doi: 10.1002/ece3.8810 (PMC9001158; doi:10.1002/ece3.8810)
Supplement: Supplementary file 1 — Figure S1 [file ECE3-12-e8810-s003.pdf]

Figure S1. Visualisation of the genomic composition analysis. The first part of each plot shows for heterozygous loci the density distribution of the relative coverage for the L (green) and R allele (brown) as well as for both alleles together (black). The second part of the plots indicates the proportion of homozygous loci for the L (green) and R allele (brown).

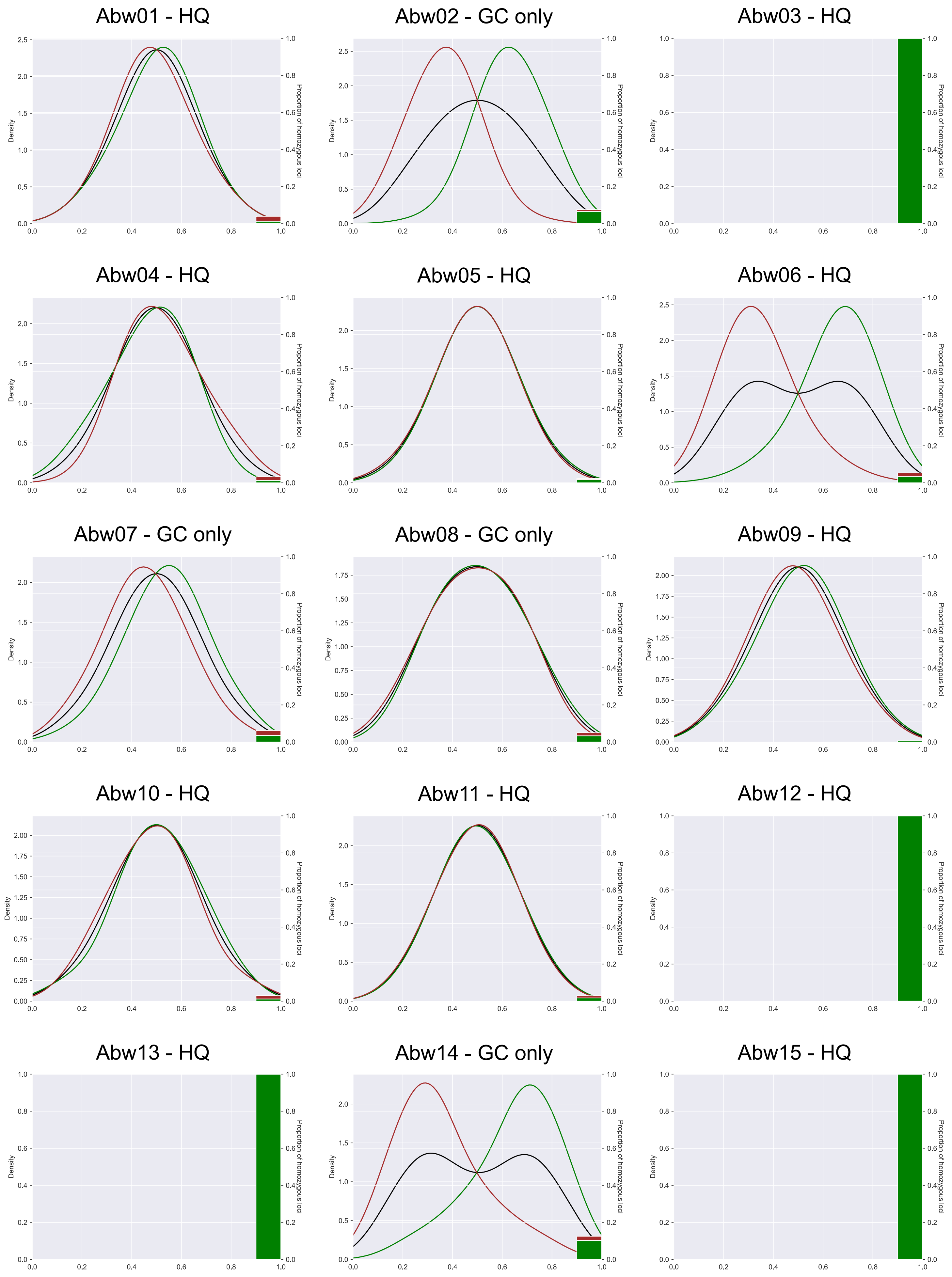
Abw01 - HQ
Abw02 - GC only
Abw03 - HQ
Abw04 - HQ
Abw05 - HQ
Abw06 - HQ
Abw07 - GC only
Abw08 - GC only
Abw09 - HQ
Abw10 - HQ
Abw11 - HQ
Abw12 - HQ
Abw13 - HQ
Abw14 - GC only
Abw15 - HQ
Density
Proportion of homozygous loci

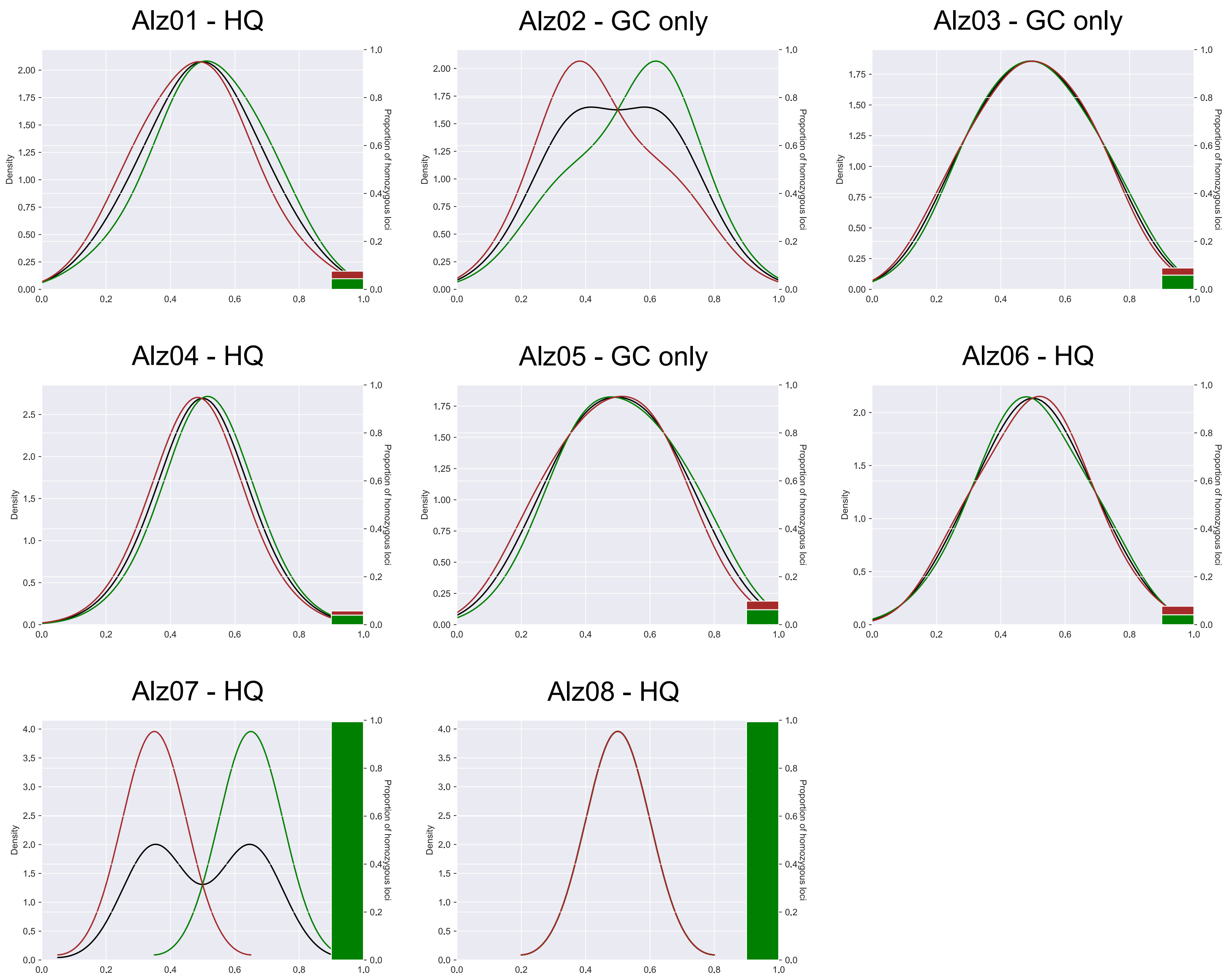

Alz01 - HQ
Alz02 - GC only
Alz03 - GC only
Alz04 - HQ
Alz05 - GC only
Alz06 - HQ
Alz07 - HQ
Alz08 - HQ
Density
Proportion of homozygous loci

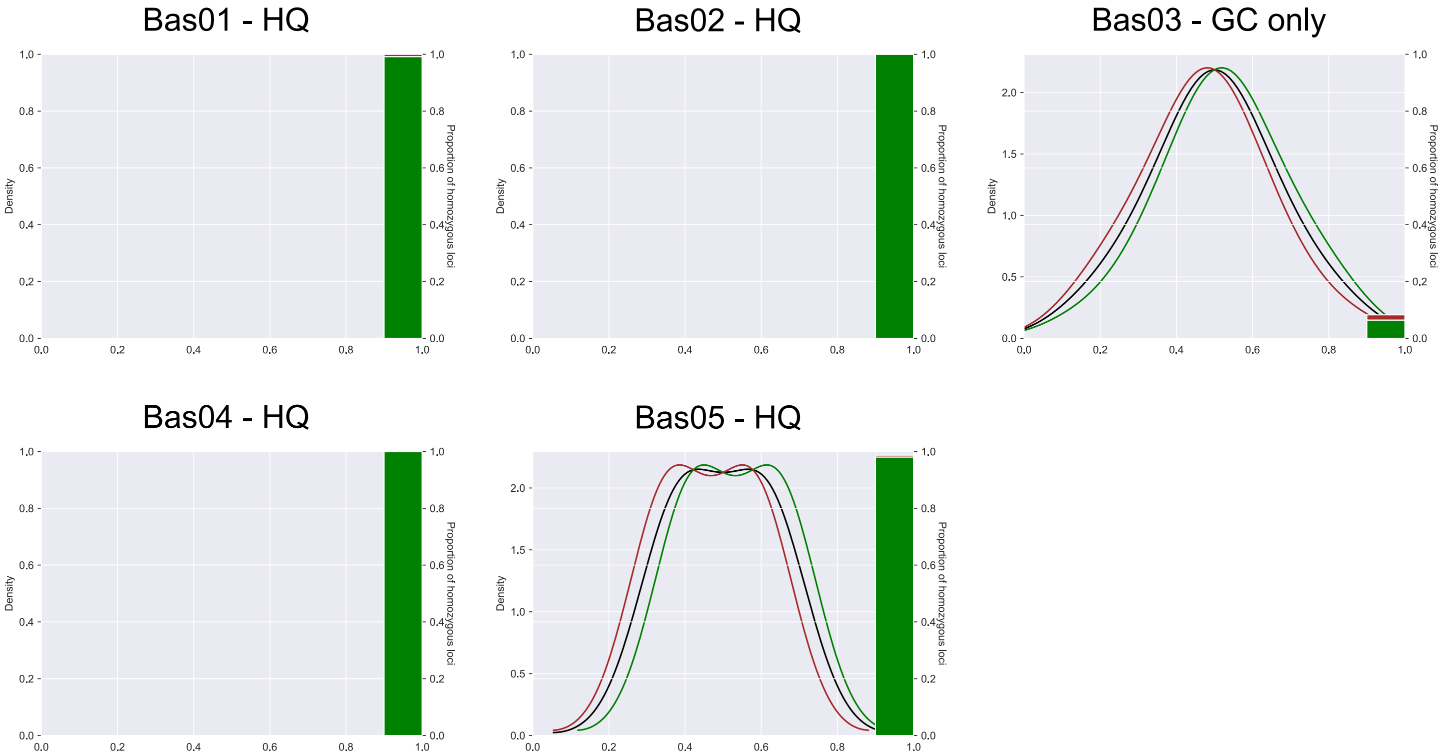

Bas01 - HQ
Bas02 - HQ
Bas03 - GC only
Bas04 - HQ
Bas05 - HQ
Density
Proportion of homozygous loci
0.0
0.2
0.4
0.6
0.8
1.0
0.5
1.5
2.0

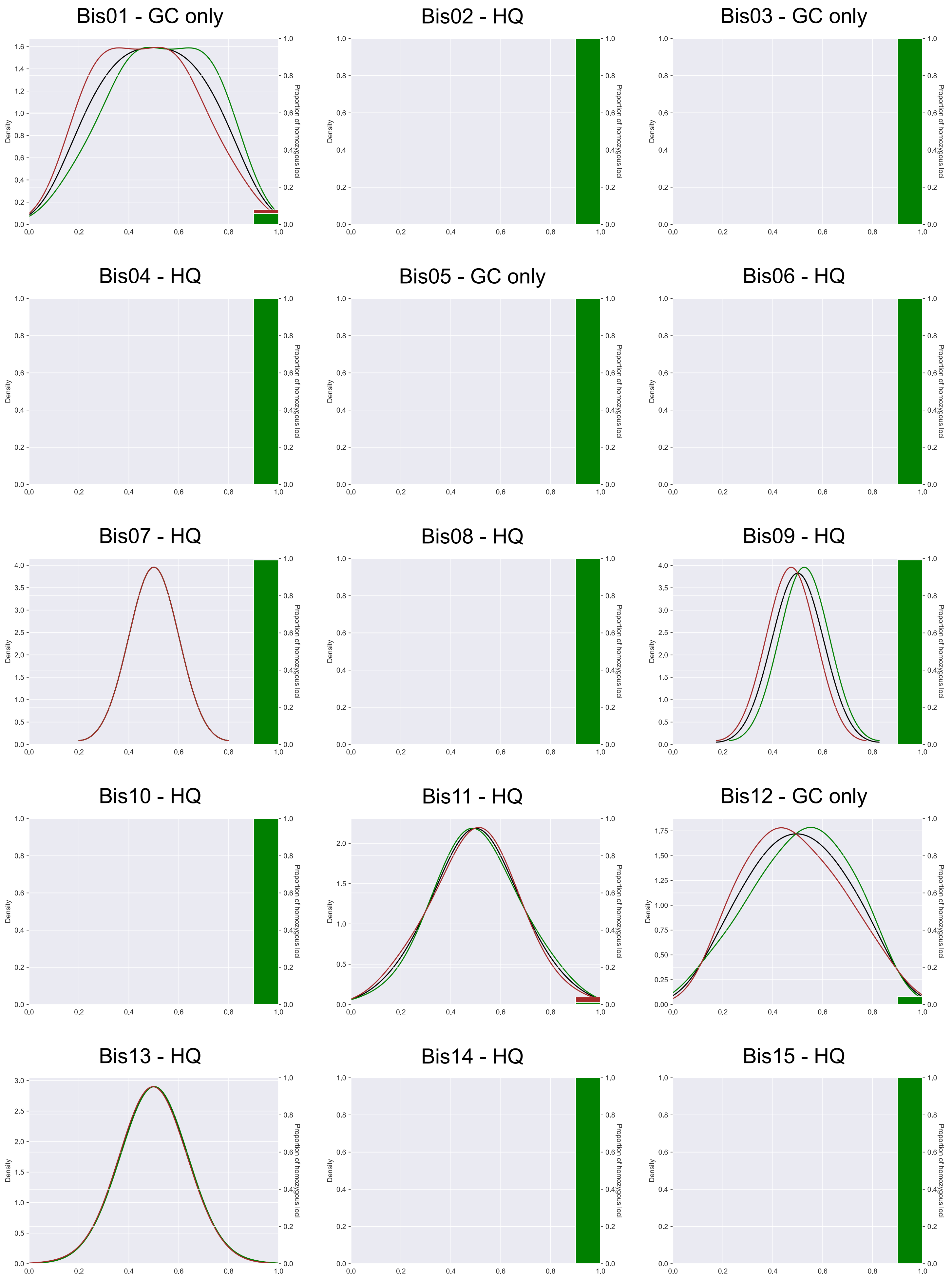
Bis01 - GC only
Bis02 - HQ
Bis03 - GC only
Bis04 - HQ
Bis05 - GC only
Bis06 - HQ
Bis07 - HQ
Bis08 - HQ
Bis09 - HQ
Bis10 - HQ
Bis11 - HQ
Bis12 - GC only
Bis13 - HQ
Bis14 - HQ
Bis15 - HQ
Density
Proportion of homozygous loci

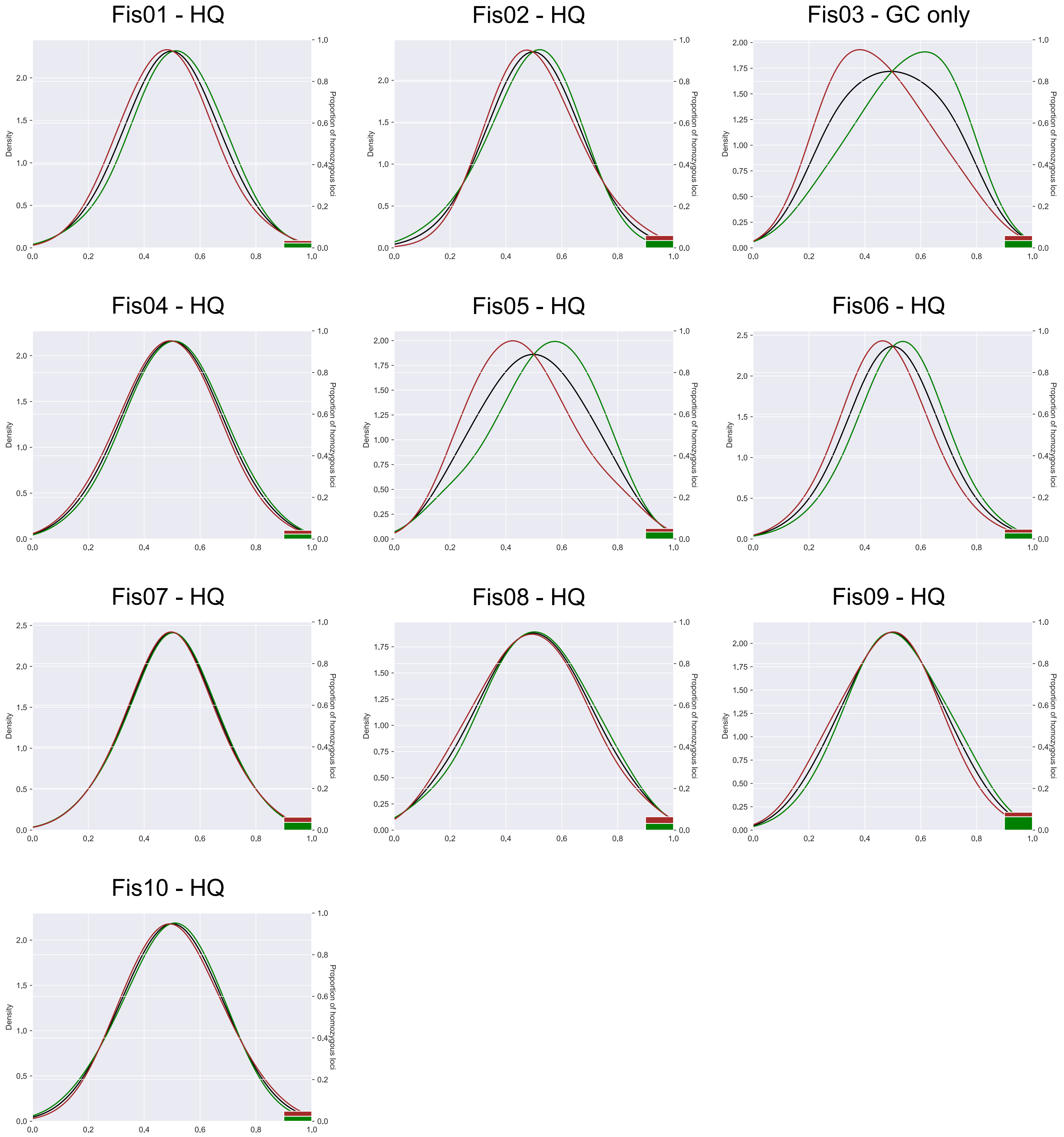

Fis01 - HQ
Fis02 - HQ
Fis03 - GC only
Fis04 - HQ
Fis05 - HQ
Fis06 - HQ
Fis07 - HQ
Fis08 - HQ
Fis09 - HQ
Fis10 - HQ
Density
Proportion of homozygous loci

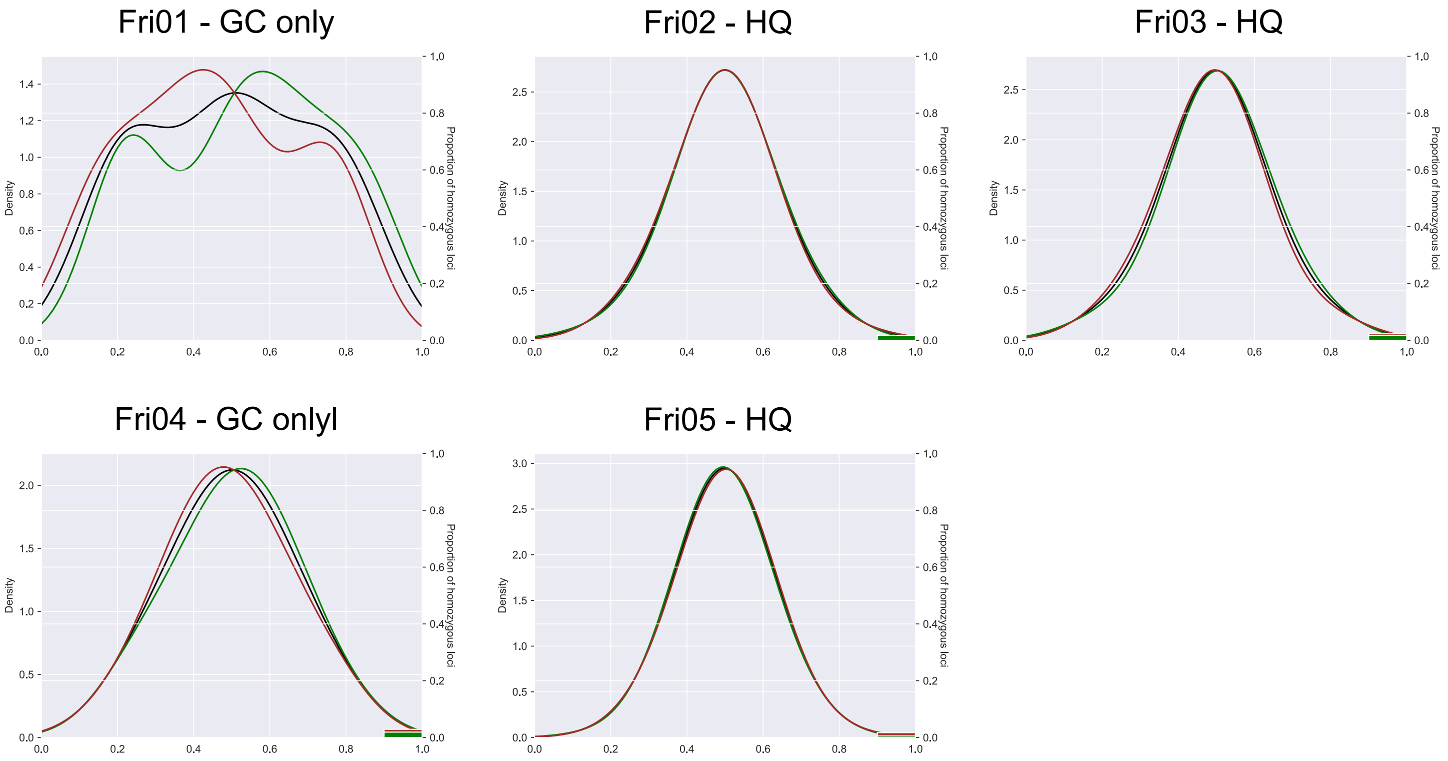
Fri01 - GC only
Fri02 - HQ
Fri03 - HQ
Fri04 - GC onlyl
Fri05 - HQ
Density
Proportion of homozygous loci

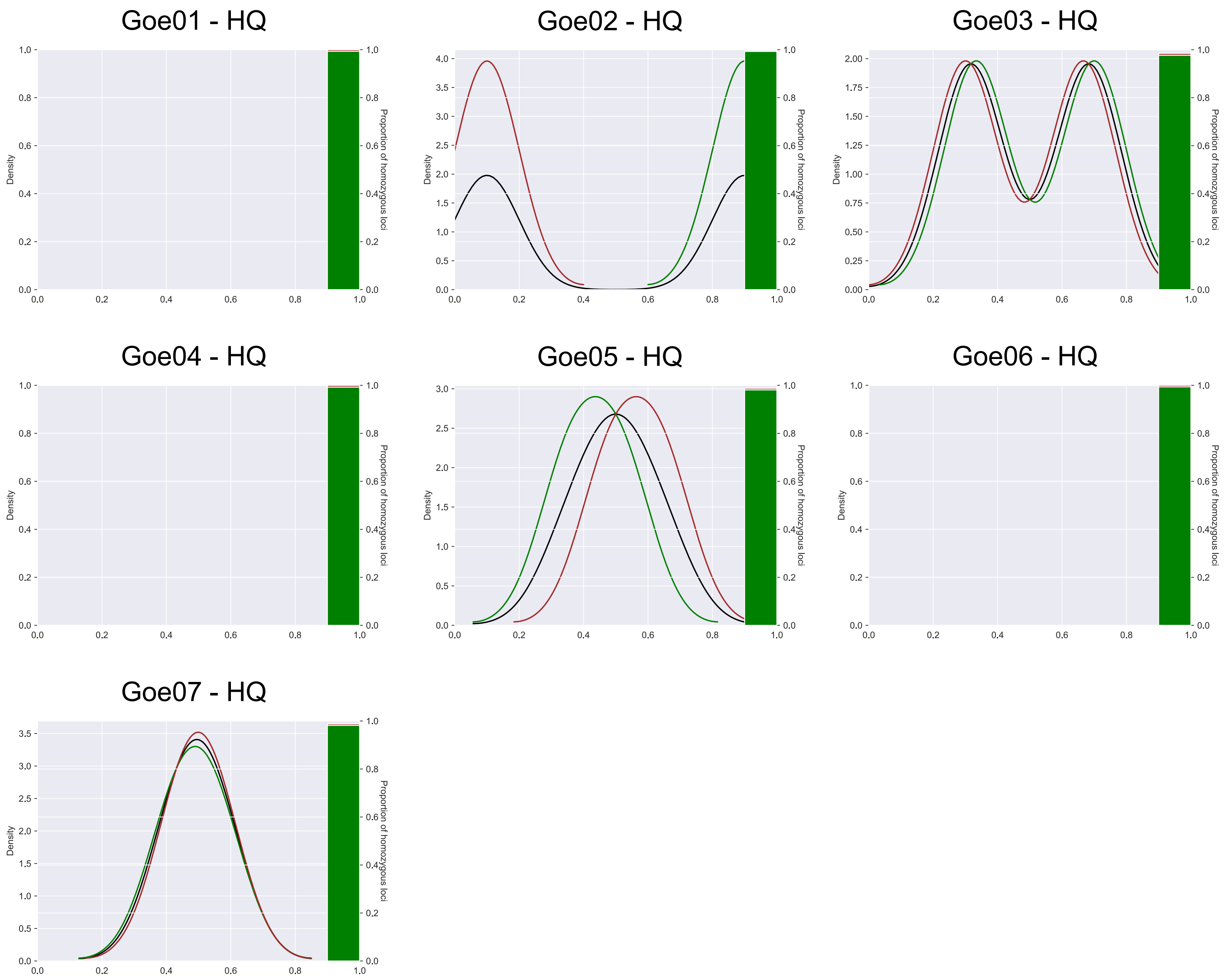
Goe01 - HQ
Goe02 - HQ
Goe03 - HQ
Goe04 - HQ
Goe05 - HQ
Goe06 - HQ
Goe07 - HQ
Density
Proportion of homozygous loci

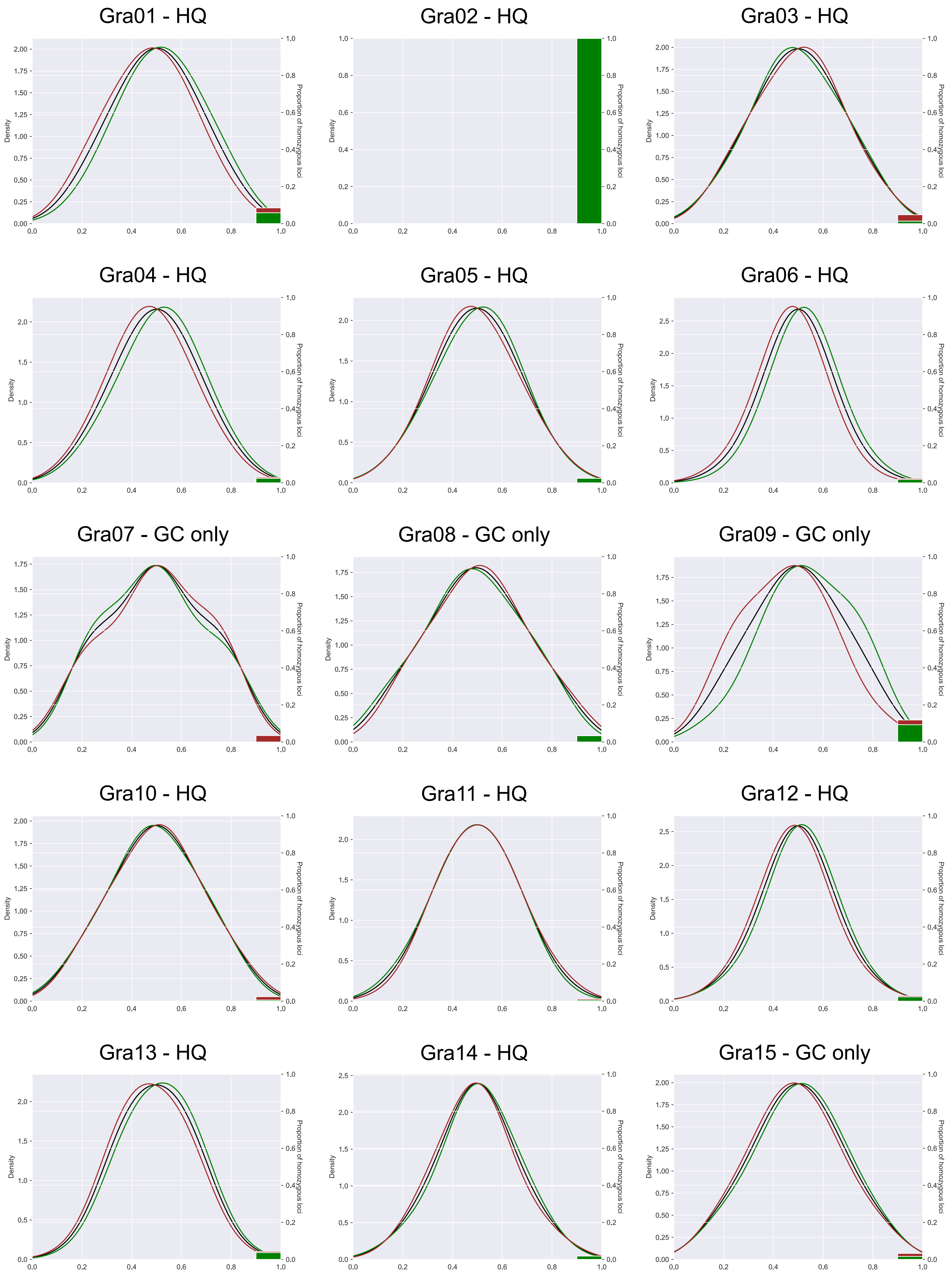
Gra01 - HQ
Gra02 - HQ
Gra03 - HQ
Gra04 - HQ
Gra05 - HQ
Gra06 - HQ
Gra07 - GC only
Gra08 - GC only
Gra09 - GC only
Gra10 - HQ
Gra11 - HQ
Gra12 - HQ
Gra13 - HQ
Gra14 - HQ
Gra15 - GC only
Density
Proportion of homozygous loci

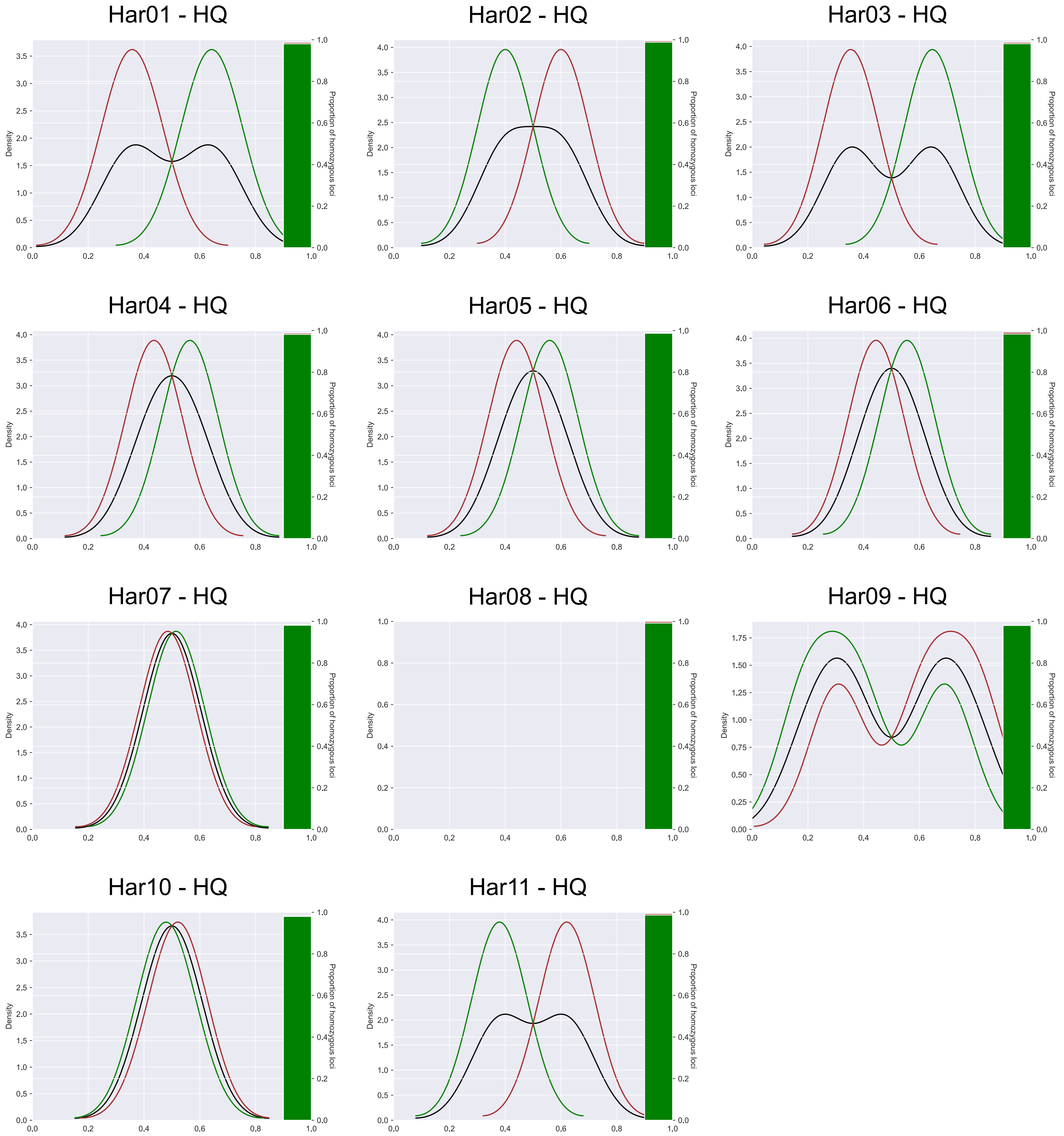
Har01 - HQ
Har02 - HQ
Har03 - HQ
Har04 - HQ
Har05 - HQ
Har06 - HQ
Har07 - HQ
Har08 - HQ
Har09 - HQ
Har10 - HQ
Har11 - HQ
Density
Proportion of homozygous loci

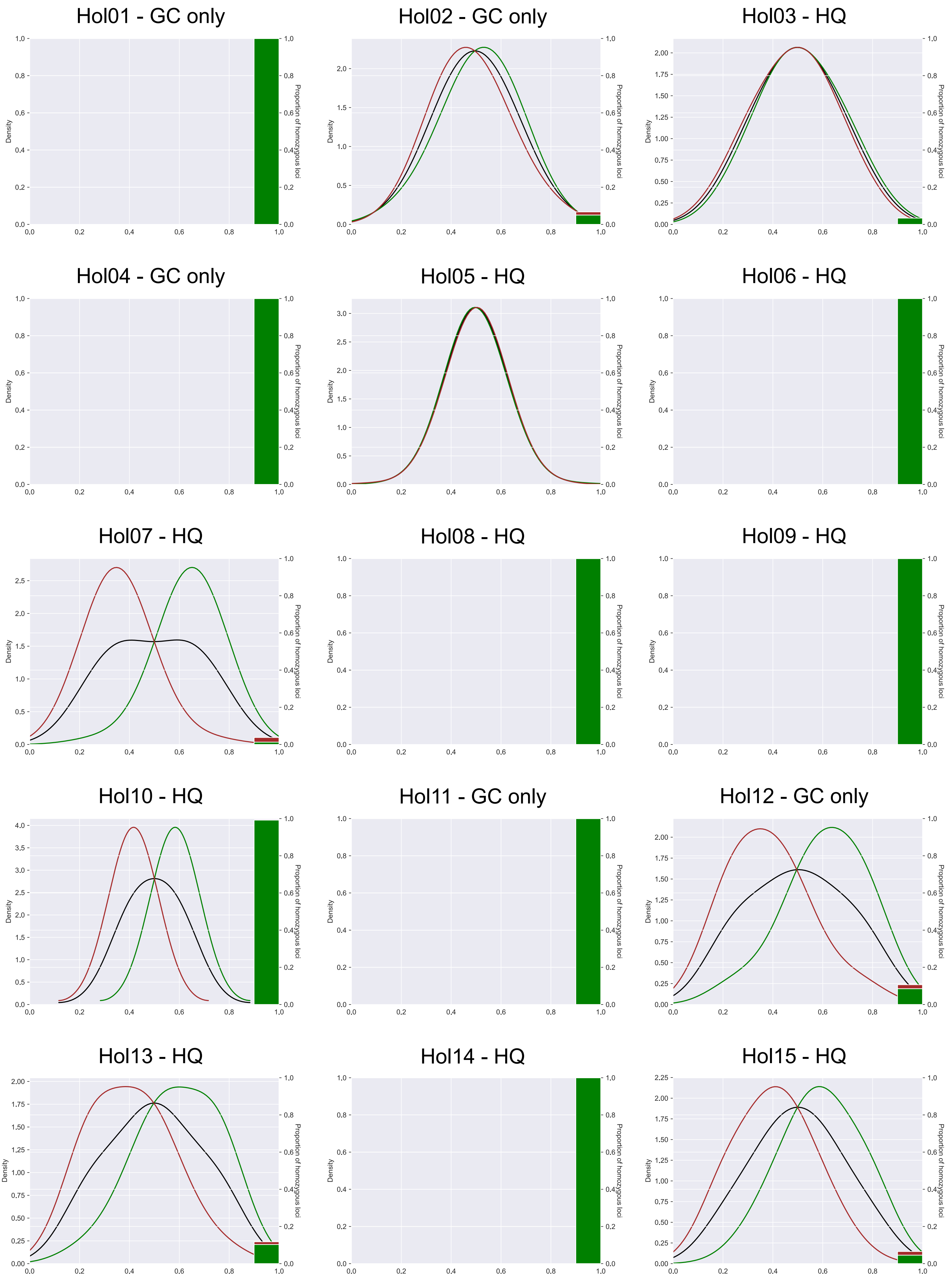
Hol01 - GC only
Hol02 - GC only
Hol03 - HQ
Hol04 - GC only
Hol05 - HQ
Hol06 - HQ
Hol07 - HQ
Hol08 - HQ
Hol09 - HQ
Hol10 - HQ
Hol11 - GC only
Hol12 - GC only
Hol13 - HQ
Hol14 - HQ
Hol15 - HQ
Density
Proportion of homozygous loci

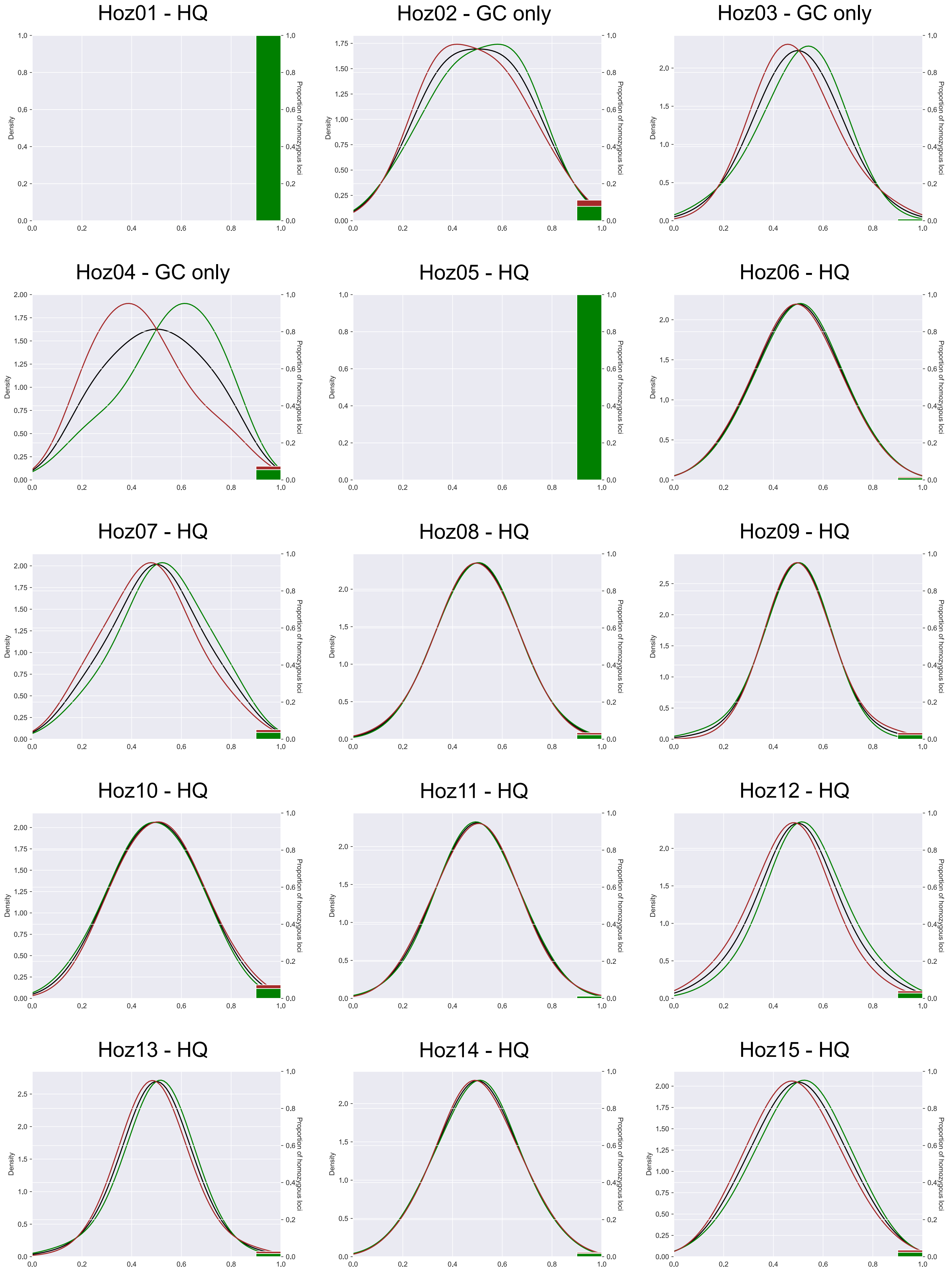

Hoz01 - HQ
Hoz02 - GC only
Hoz03 - GC only
Hoz04 - GC only
Hoz05 - HQ
Hoz06 - HQ
Hoz07 - HQ
Hoz08 - HQ
Hoz09 - HQ
Hoz10 - HQ
Hoz11 - HQ
Hoz12 - HQ
Hoz13 - HQ
Hoz14 - HQ
Hoz15 - HQ
Density
Proportion of homozygous loci
0.0
0.2
0.4
0.6
0.8
1.0

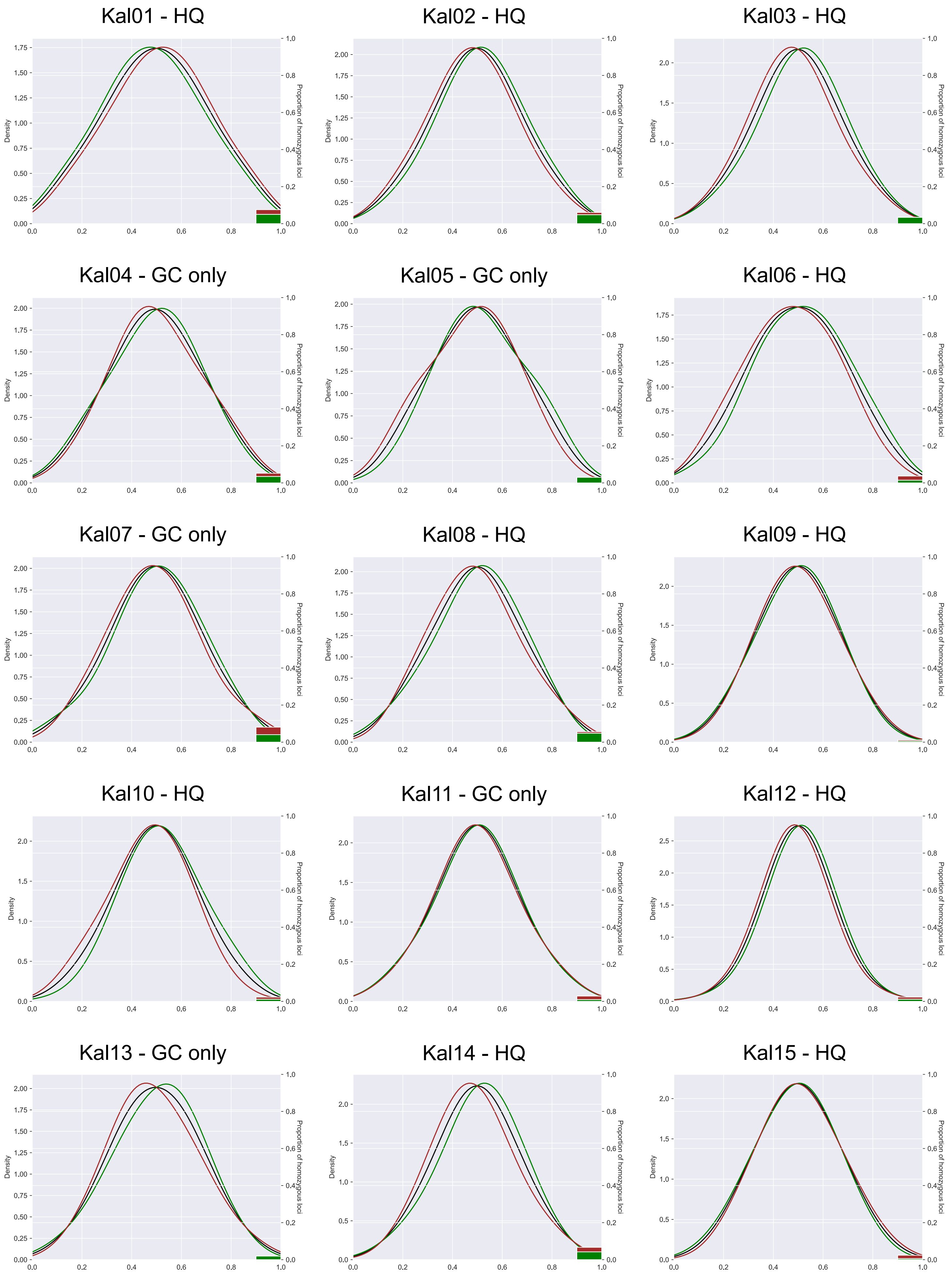
Kal01 - HQ
Kal02 - HQ
Kal03 - HQ
Kal04 - GC only
Kal05 - GC only
Kal06 - HQ
Kal07 - GC only
Kal08 - HQ
Kal09 - HQ
Kal10 - HQ
Kal11 - GC only
Kal12 - HQ
Kal13 - GC only
Kal14 - HQ
Kal15 - HQ
Density
Proportion of homozygous loci

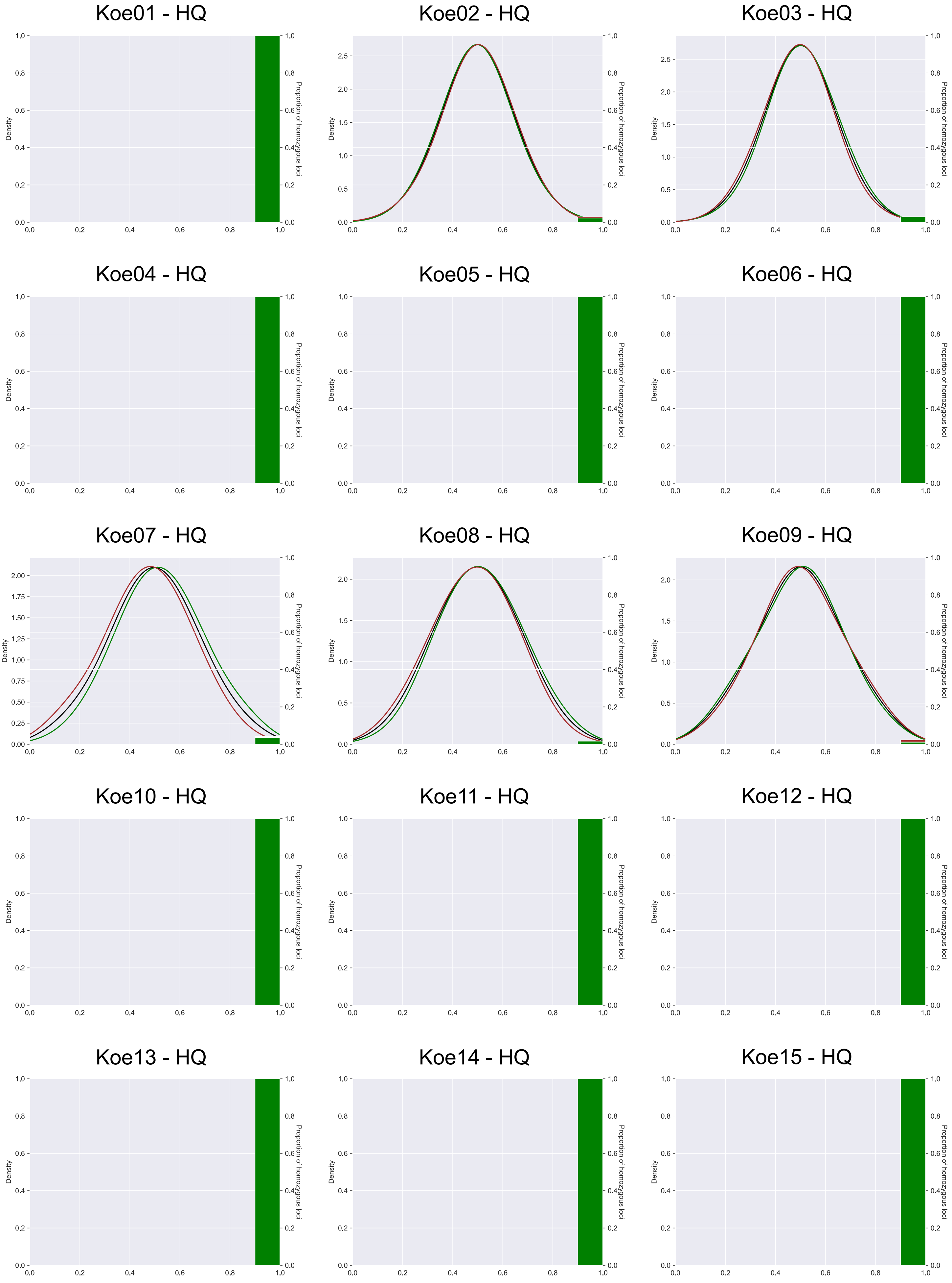

Koe01 - HQ
Koe02 - HQ
Koe03 - HQ
Koe04 - HQ
Koe05 - HQ
Koe06 - HQ
Koe07 - HQ
Koe08 - HQ
Koe09 - HQ
Koe10 - HQ
Koe11 - HQ
Koe12 - HQ
Koe13 - HQ
Koe14 - HQ
Koe15 - HQ
Density
Proportion of homozygous loci

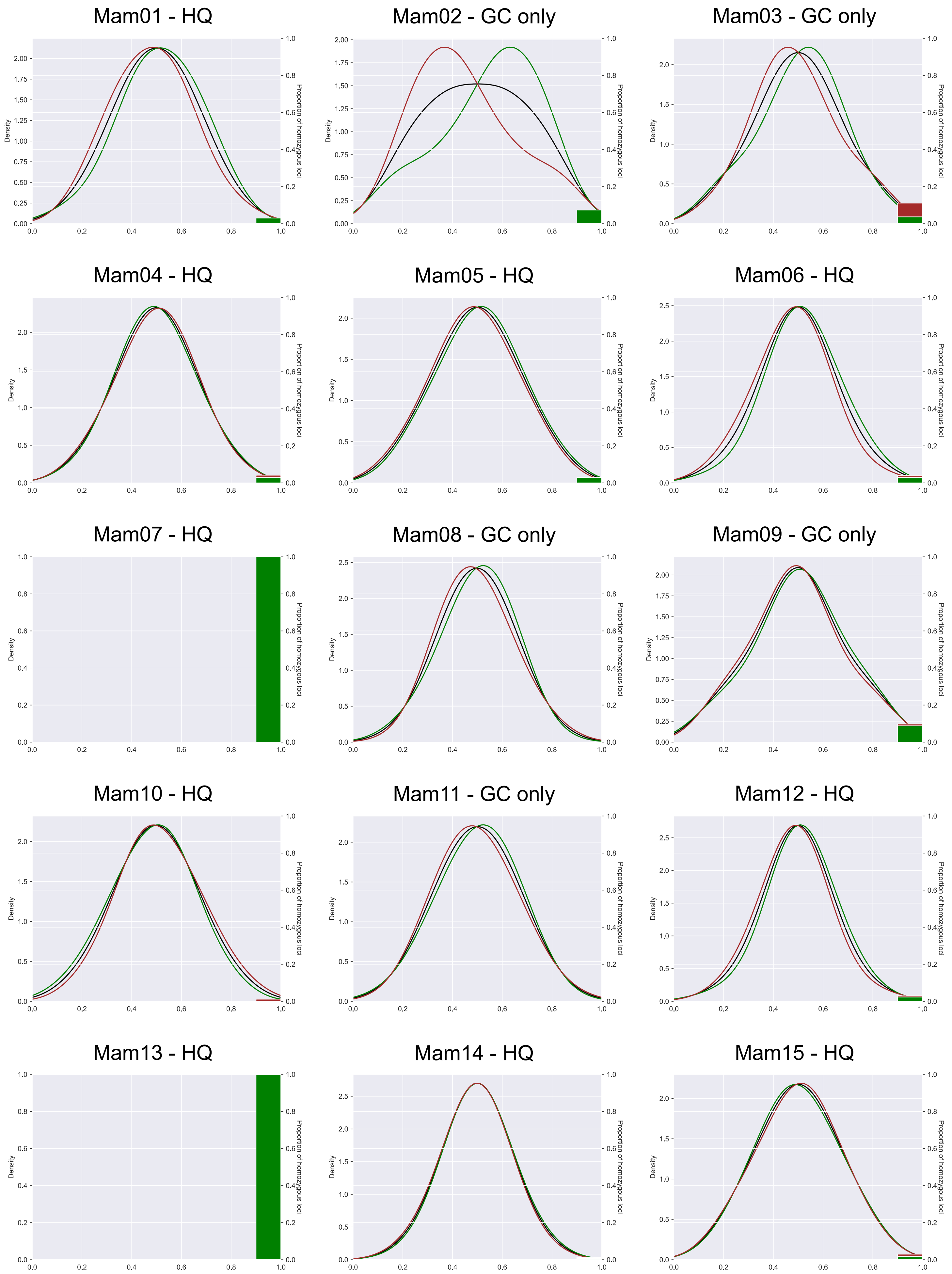
Mam01 - HQ
Mam02 - GC only
Mam03 - GC only
Mam04 - HQ
Mam05 - HQ
Mam06 - HQ
Mam07 - HQ
Mam08 - GC only
Mam09 - GC only
Mam10 - HQ
Mam11 - GC only
Mam12 - HQ
Mam13 - HQ
Mam14 - HQ
Mam15 - HQ
Density
Proportion of homozygous loci

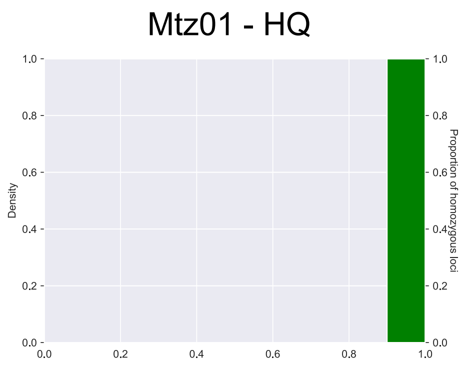

Mtz01 - HQ
Density
Proportion of homozygous loci
0.0
0.2
0.4
0.6
0.8
1.0

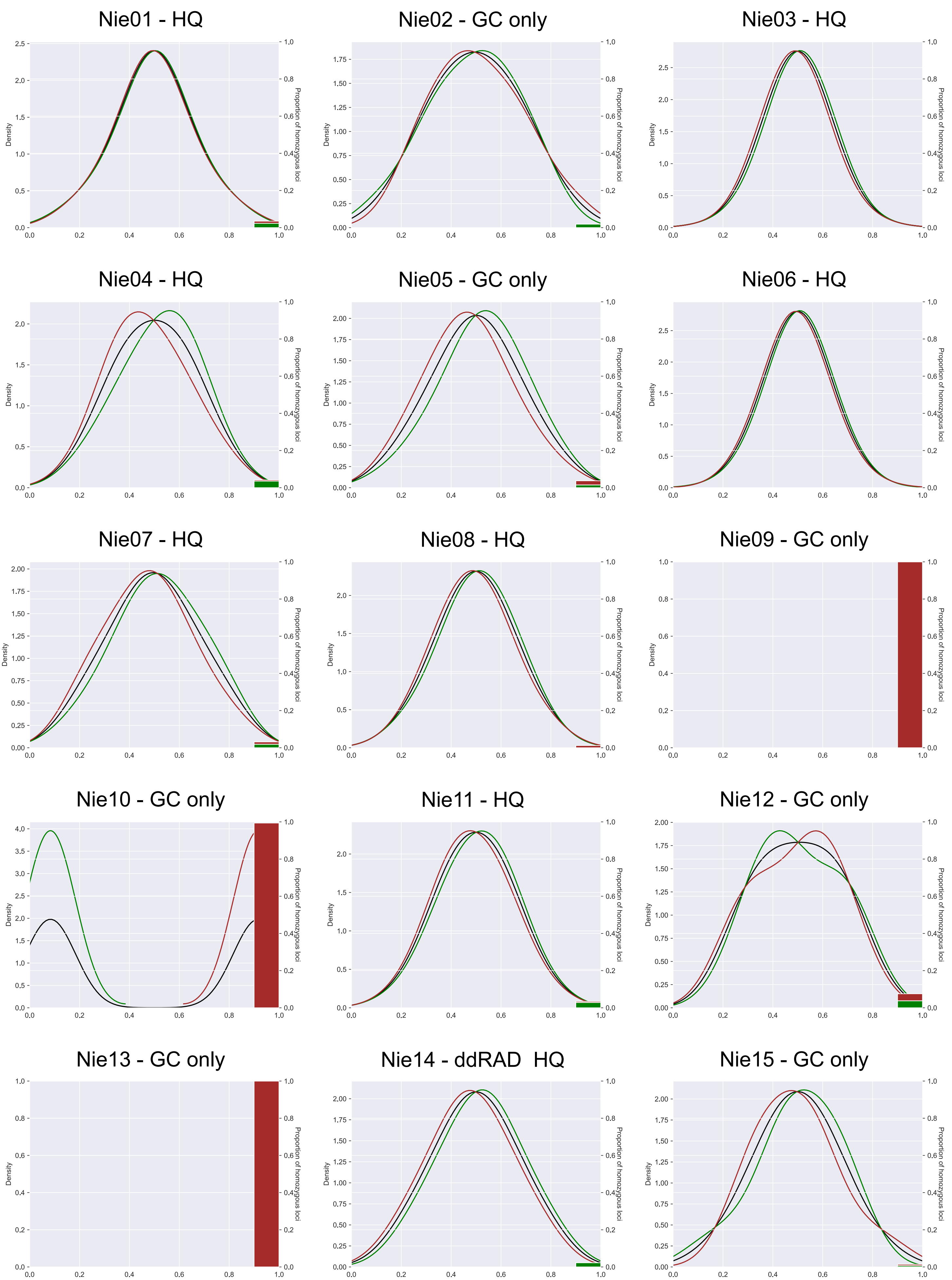
Nie01 - HQ
Nie02 - GC only
Nie03 - HQ
Nie04 - HQ
Nie05 - GC only
Nie06 - HQ
Nie07 - HQ
Nie08 - HQ
Nie09 - GC only
Nie10 - GC only
Nie11 - HQ
Nie12 - GC only
Nie13 - GC only
Nie14 - ddRAD HQ
Nie15 - GC only
Density
Proportion of homozygous loci

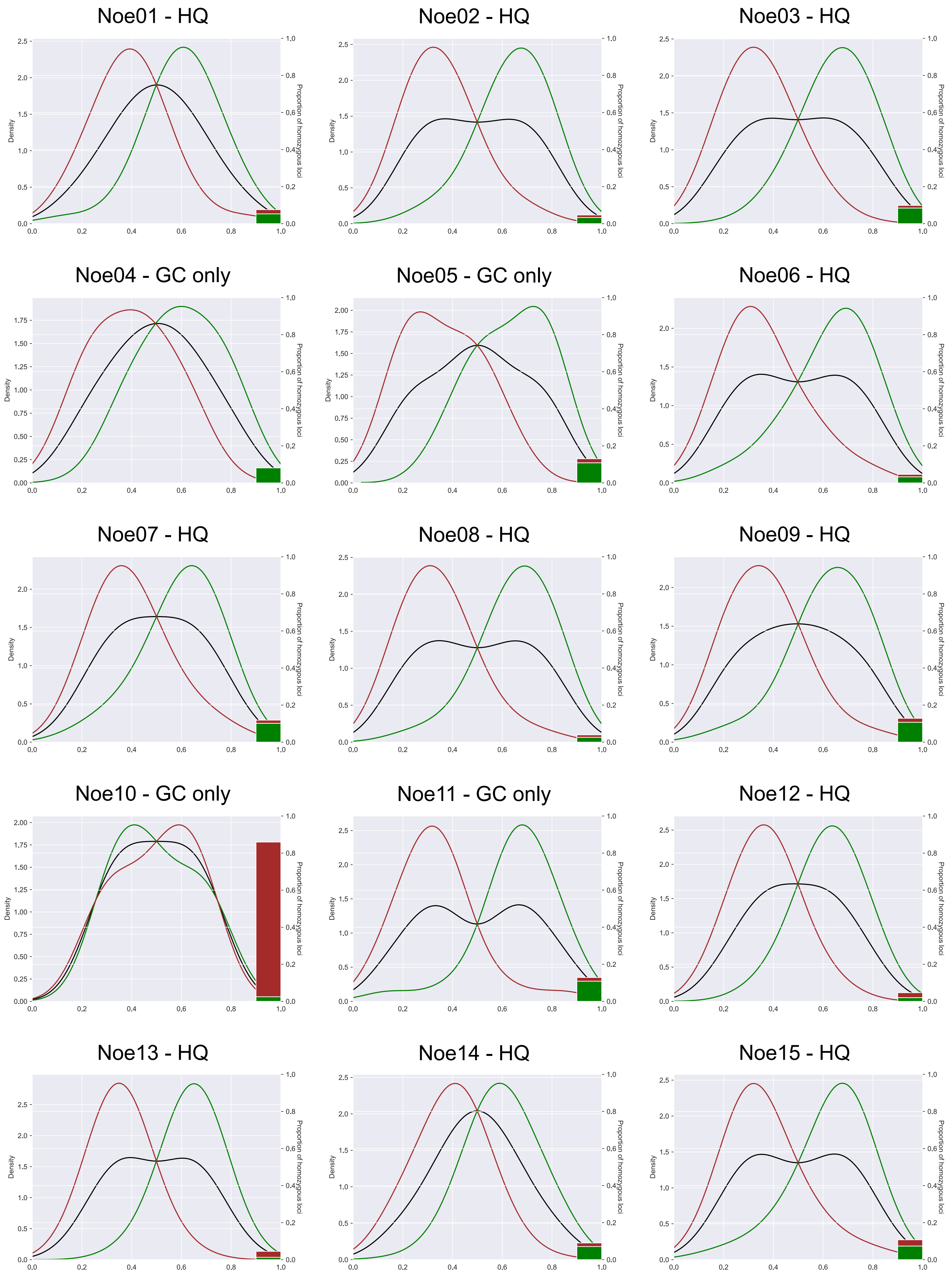
Noe01 - HQ
Noe02 - HQ
Noe03 - HQ
Noe04 - GC only
Noe05 - GC only
Noe06 - HQ
Noe07 - HQ
Noe08 - HQ
Noe09 - HQ
Noe10 - GC only
Noe11 - GC only
Noe12 - HQ
Noe13 - HQ
Noe14 - HQ
Noe15 - HQ
Density
Proportion of homozygous loci

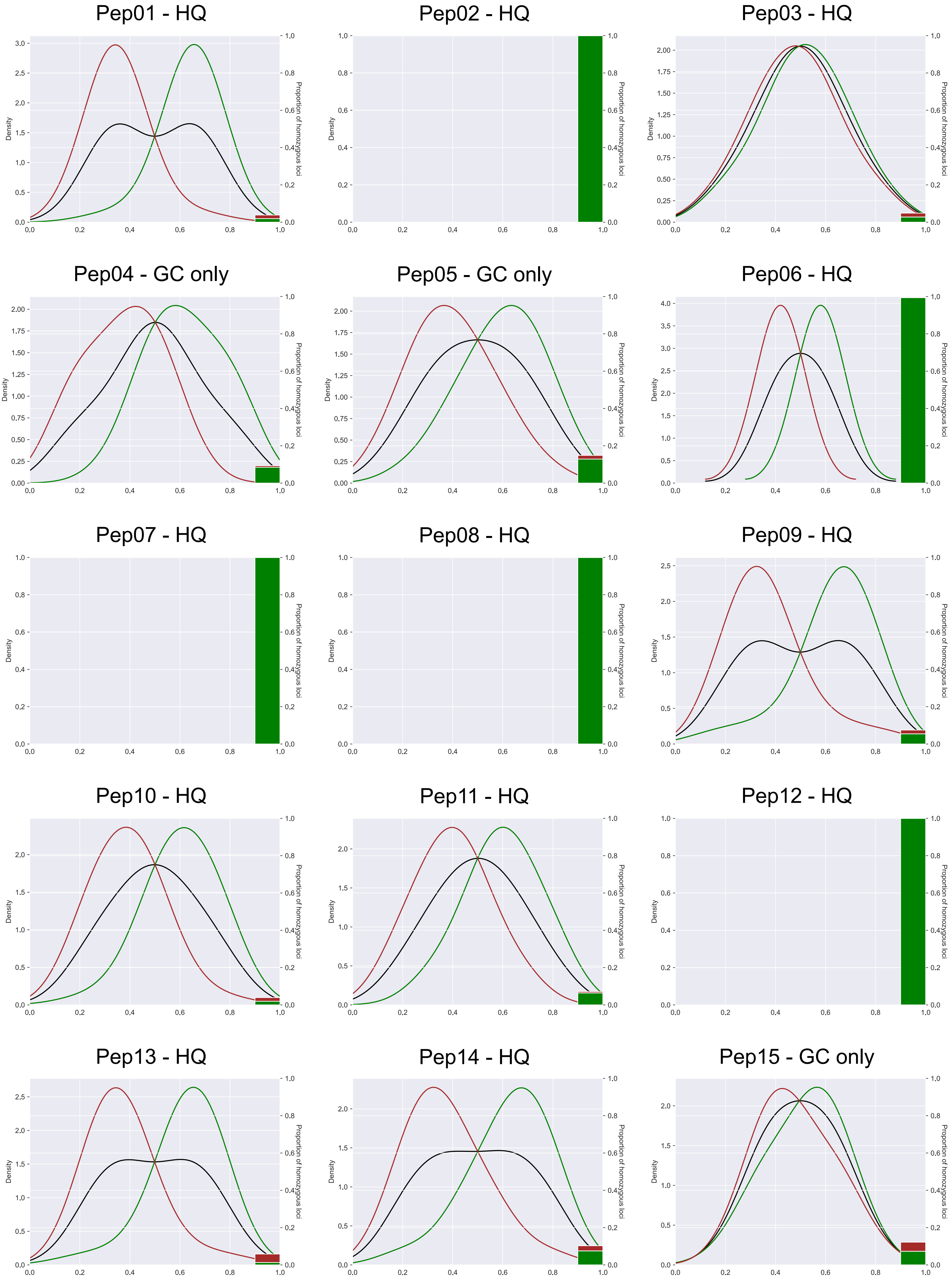
Pep01 - HQ
Pep02 - HQ
Pep03 - HQ
Pep04 - GC only
Pep05 - GC only
Pep06 - HQ
Pep07 - HQ
Pep08 - HQ
Pep09 - HQ
Pep10 - HQ
Pep11 - HQ
Pep12 - HQ
Pep13 - HQ
Pep14 - HQ
Pep15 - GC only
Density
Proportion of homozygous loci

Pep16 - GC only

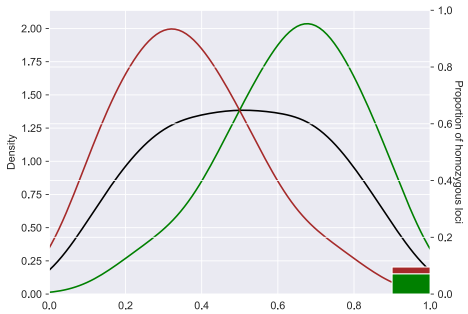

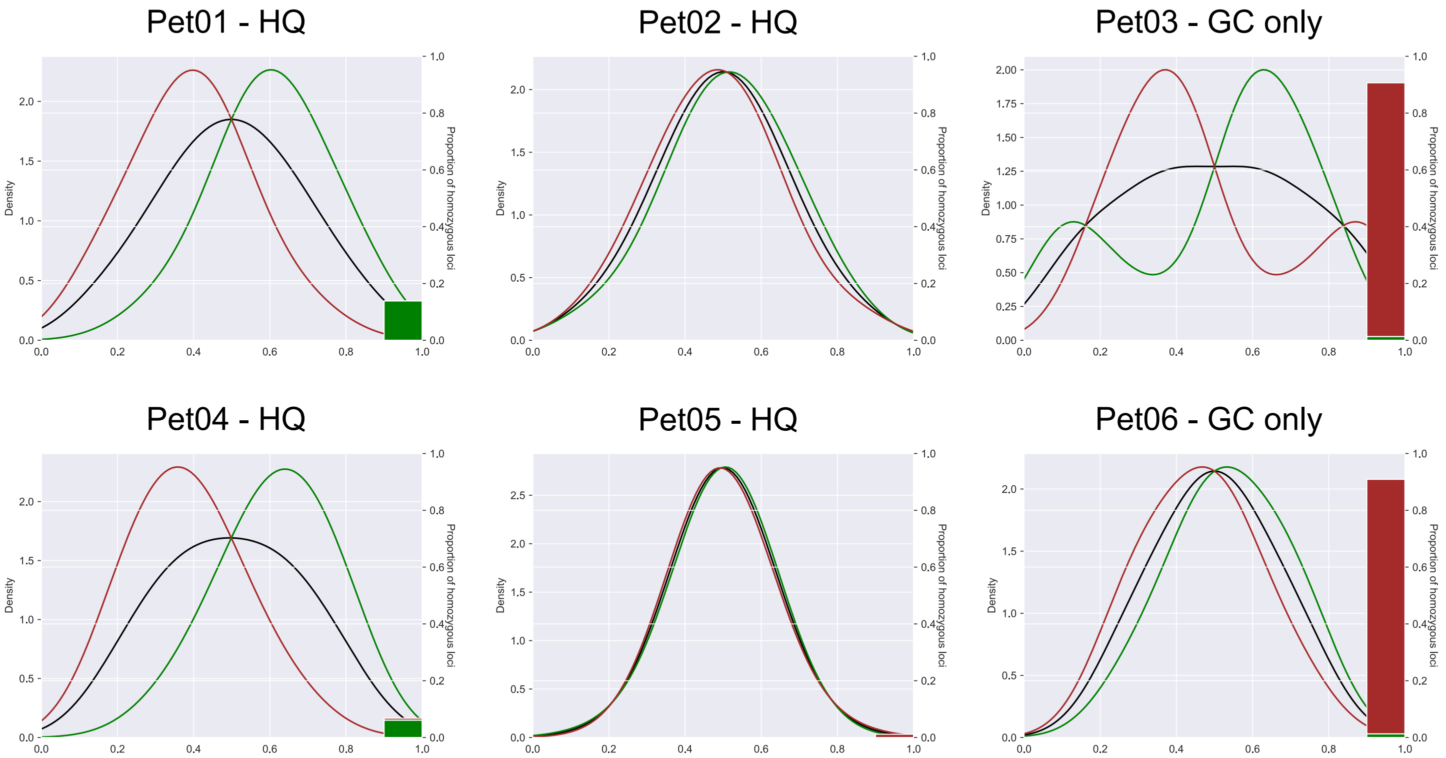

Pet01 - HQ
Pet02 - HQ
Pet03 - GC only
Pet04 - HQ
Pet05 - HQ
Pet06 - GC only
Density
Proportion of homozygous loci

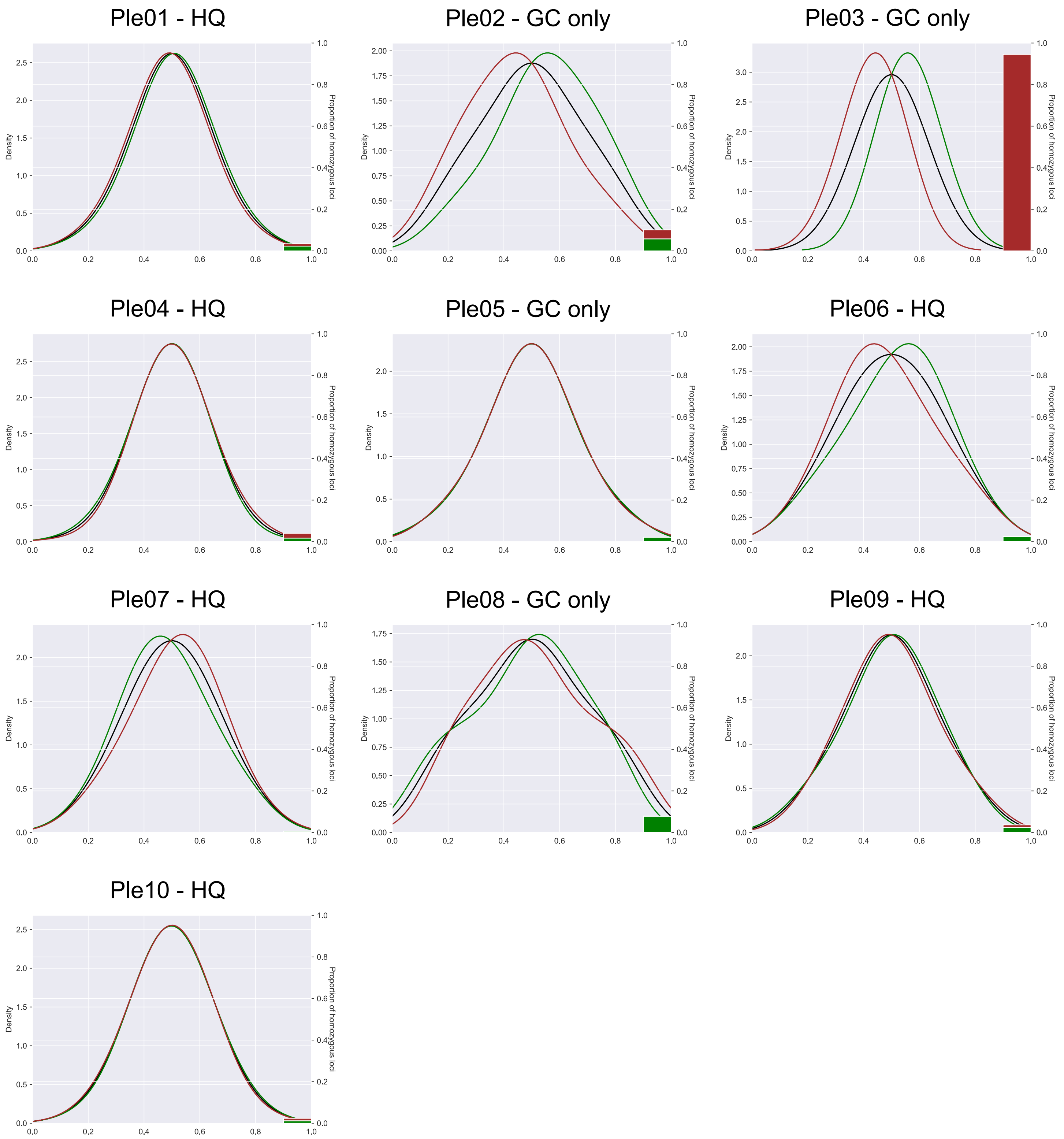

Ple01 - HQ
Ple02 - GC only
Ple03 - GC only
Ple04 - HQ
Ple05 - GC only
Ple06 - HQ
Ple07 - HQ
Ple08 - GC only
Ple09 - HQ
Ple10 - HQ
Density
Proportion of homozygous loci

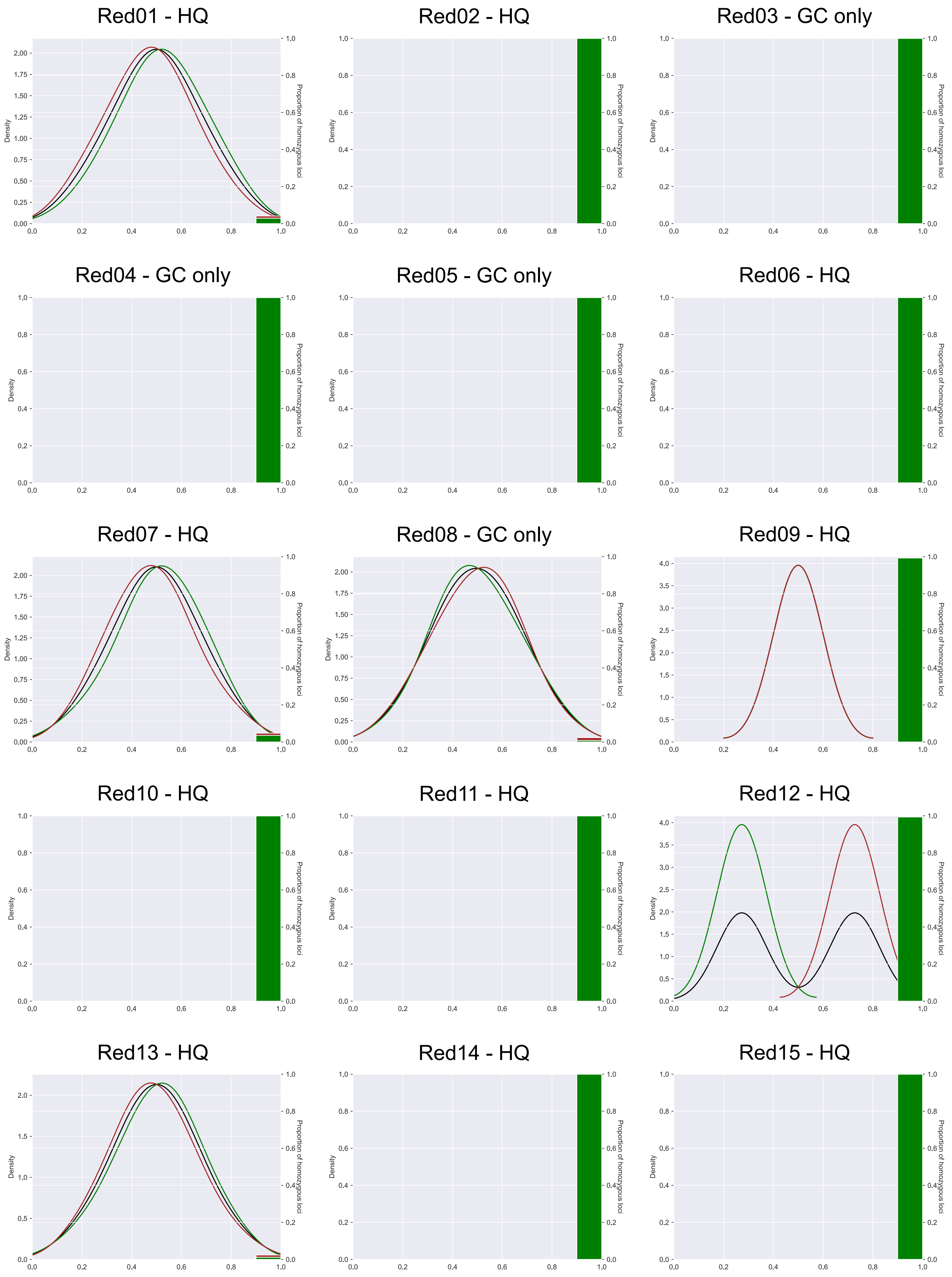

Red01 - HQ
Red02 - HQ
Red03 - GC only
Red04 - GC only
Red05 - GC only
Red06 - HQ
Red07 - HQ
Red08 - GC only
Red09 - HQ
Red10 - HQ
Red11 - HQ
Red12 - HQ
Red13 - HQ
Red14 - HQ
Red15 - HQ
Density
Proportion of homozygous loci

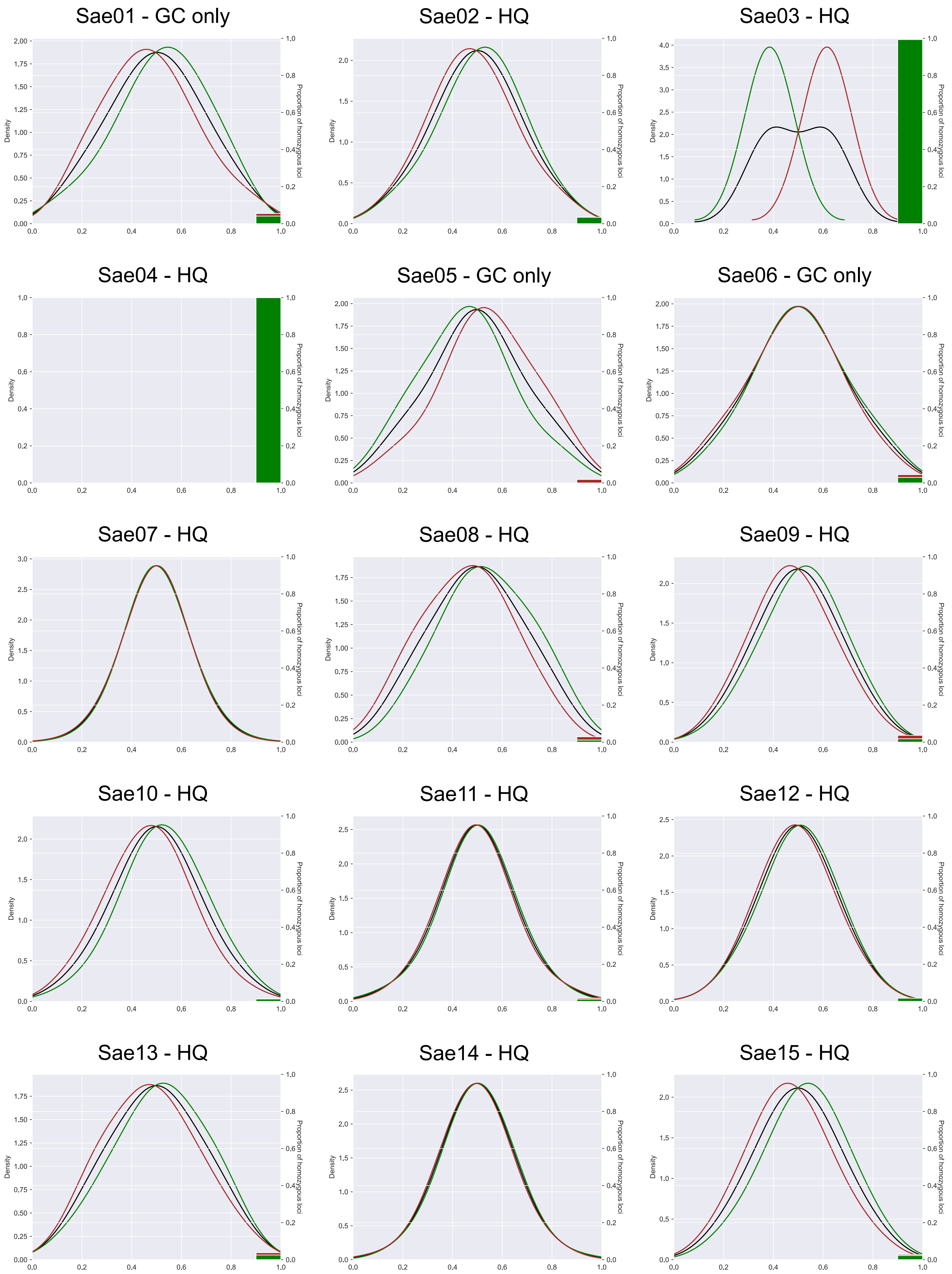

Sae01 - GC only
Sae02 - HQ
Sae03 - HQ
Sae04 - HQ
Sae05 - GC only
Sae06 - GC only
Sae07 - HQ
Sae08 - HQ
Sae09 - HQ
Sae10 - HQ
Sae11 - HQ
Sae12 - HQ
Sae13 - HQ
Sae14 - HQ
Sae15 - HQ
Density
Proportion of homozygous loci

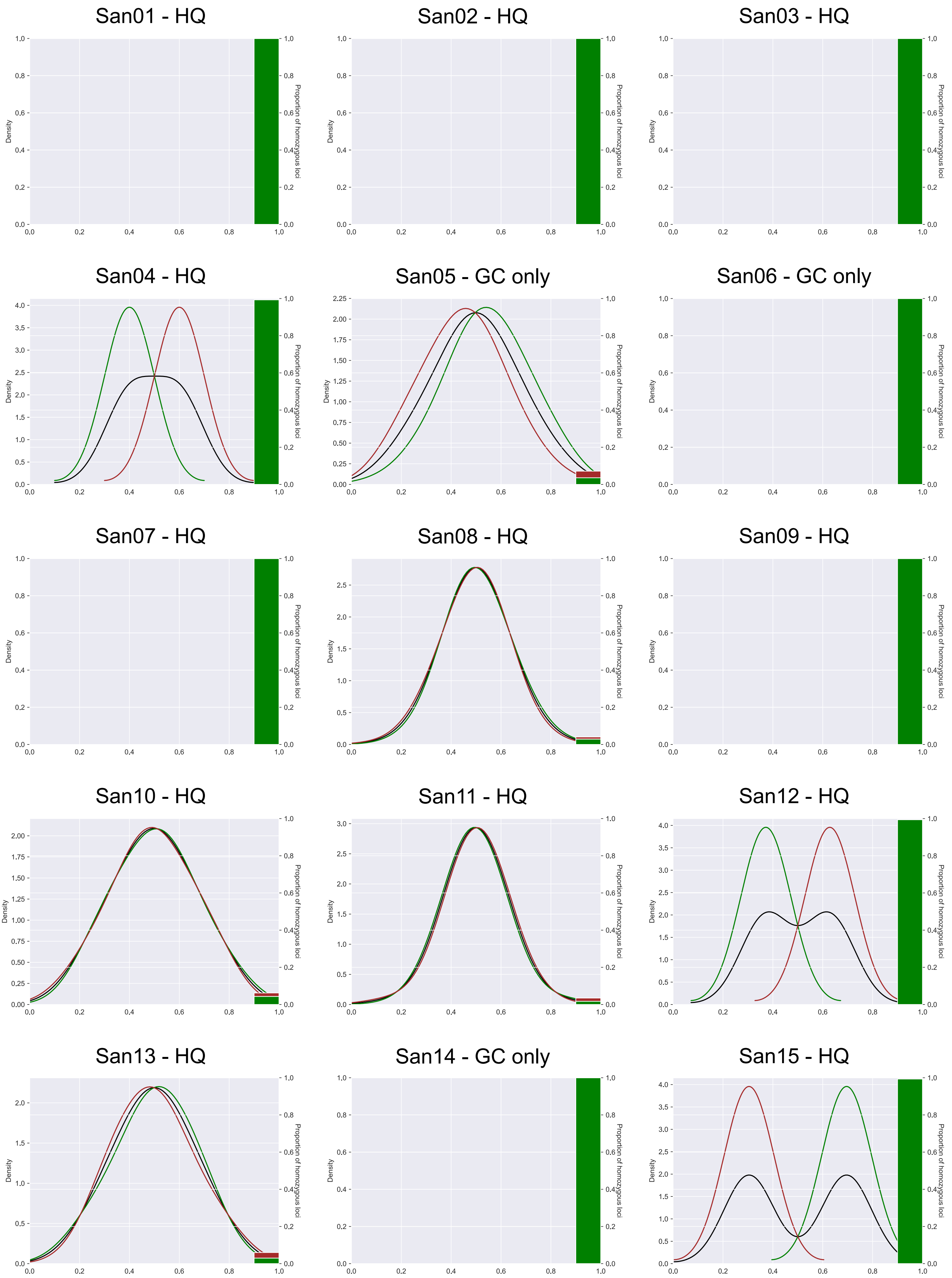

San01 - HQ
San02 - HQ
San03 - HQ
San04 - HQ
San05 - GC only
San06 - GC only
San07 - HQ
San08 - HQ
San09 - HQ
San10 - HQ
San11 - HQ
San12 - HQ
San13 - HQ
San14 - GC only
San15 - HQ
Density
Proportion of homozygous loci

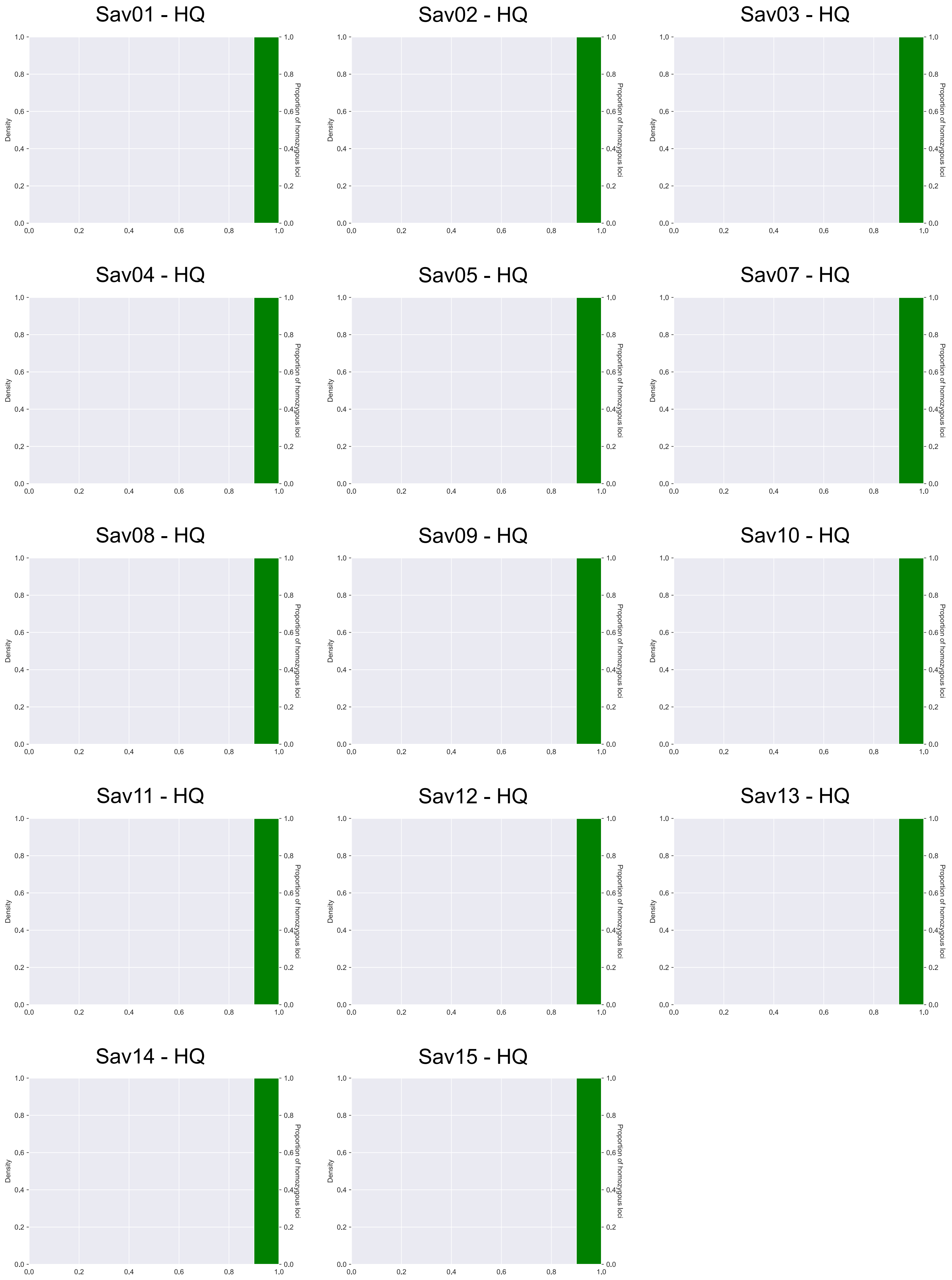
Sav01 - HQ
Sav02 - HQ
Sav03 - HQ
Sav04 - HQ
Sav05 - HQ
Sav07 - HQ
Sav08 - HQ
Sav09 - HQ
Sav10 - HQ
Sav11 - HQ
Sav12 - HQ
Sav13 - HQ
Sav14 - HQ
Sav15 - HQ
Density
Proportion of homozygous loci

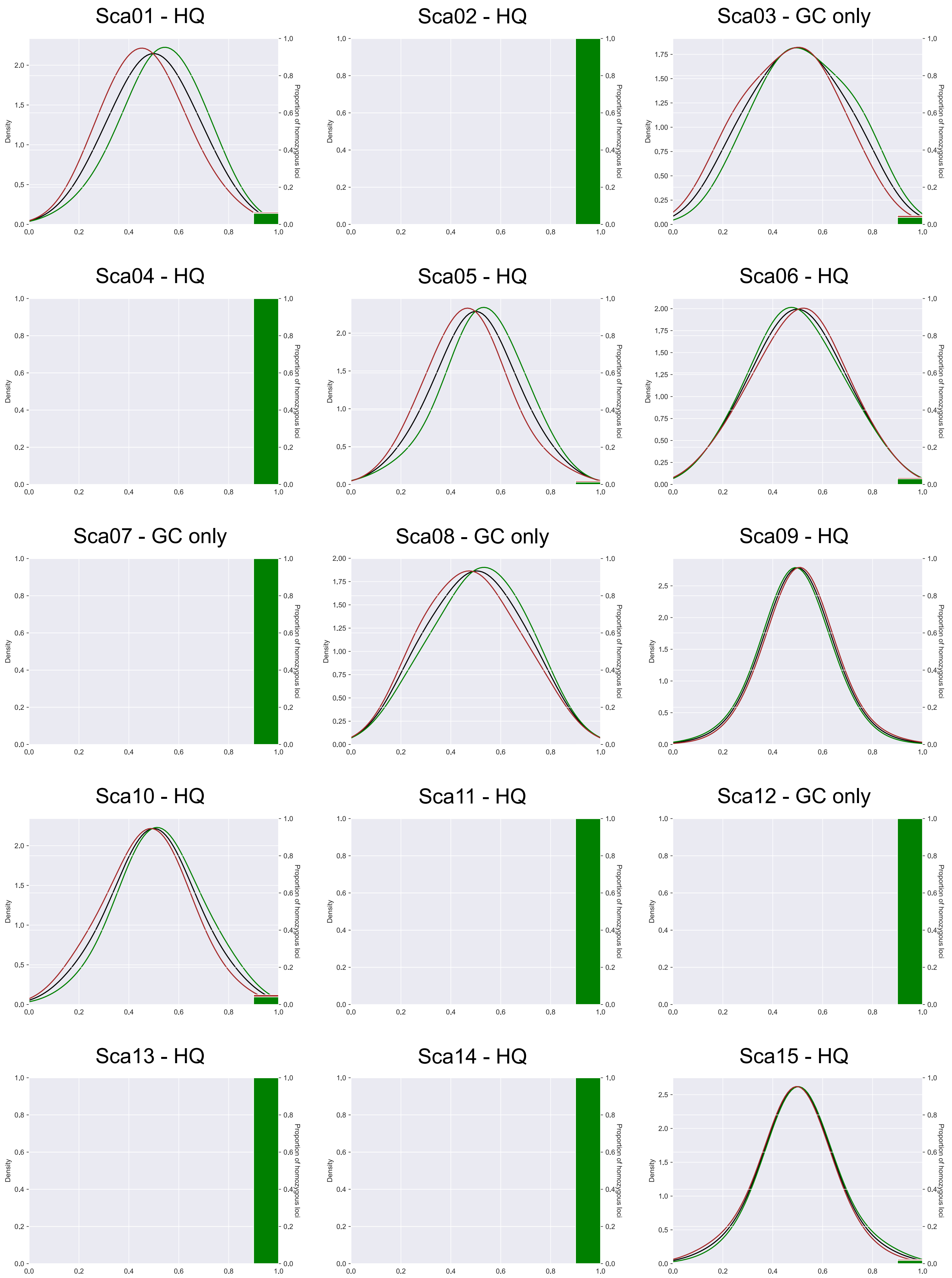
Sca01 - HQ
Sca02 - HQ
Sca03 - GC only
Sca04 - HQ
Sca05 - HQ
Sca06 - HQ
Sca07 - GC only
Sca08 - GC only
Sca09 - HQ
Sca10 - HQ
Sca11 - HQ
Sca12 - GC only
Sca13 - HQ
Sca14 - HQ
Sca15 - HQ
Density
Proportion of homozygous loci

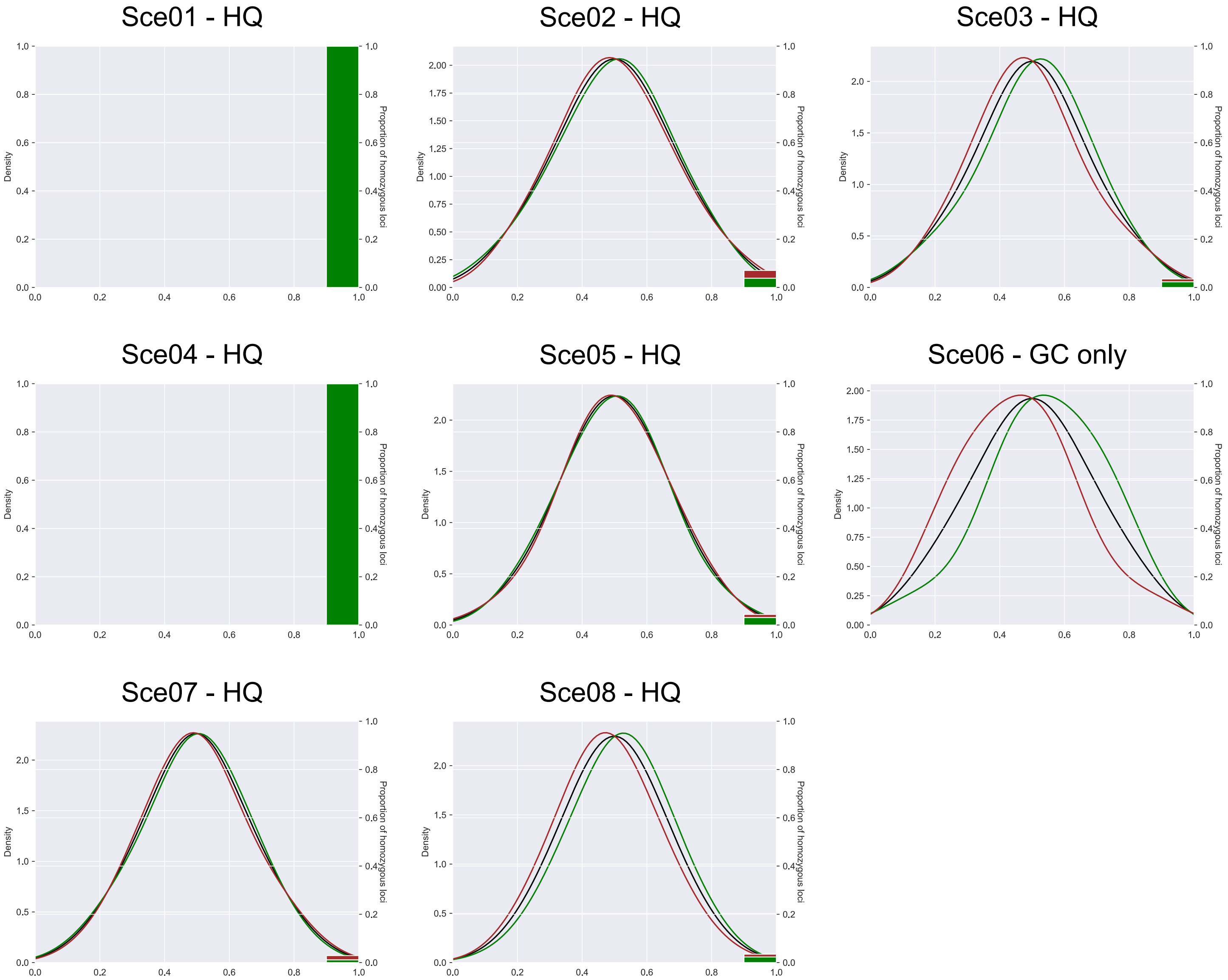

Sce01 - HQ
Sce02 - HQ
Sce03 - HQ
Sce04 - HQ
Sce05 - HQ
Sce06 - GC only
Sce07 - HQ
Sce08 - HQ
Density
Proportion of homozygous loci

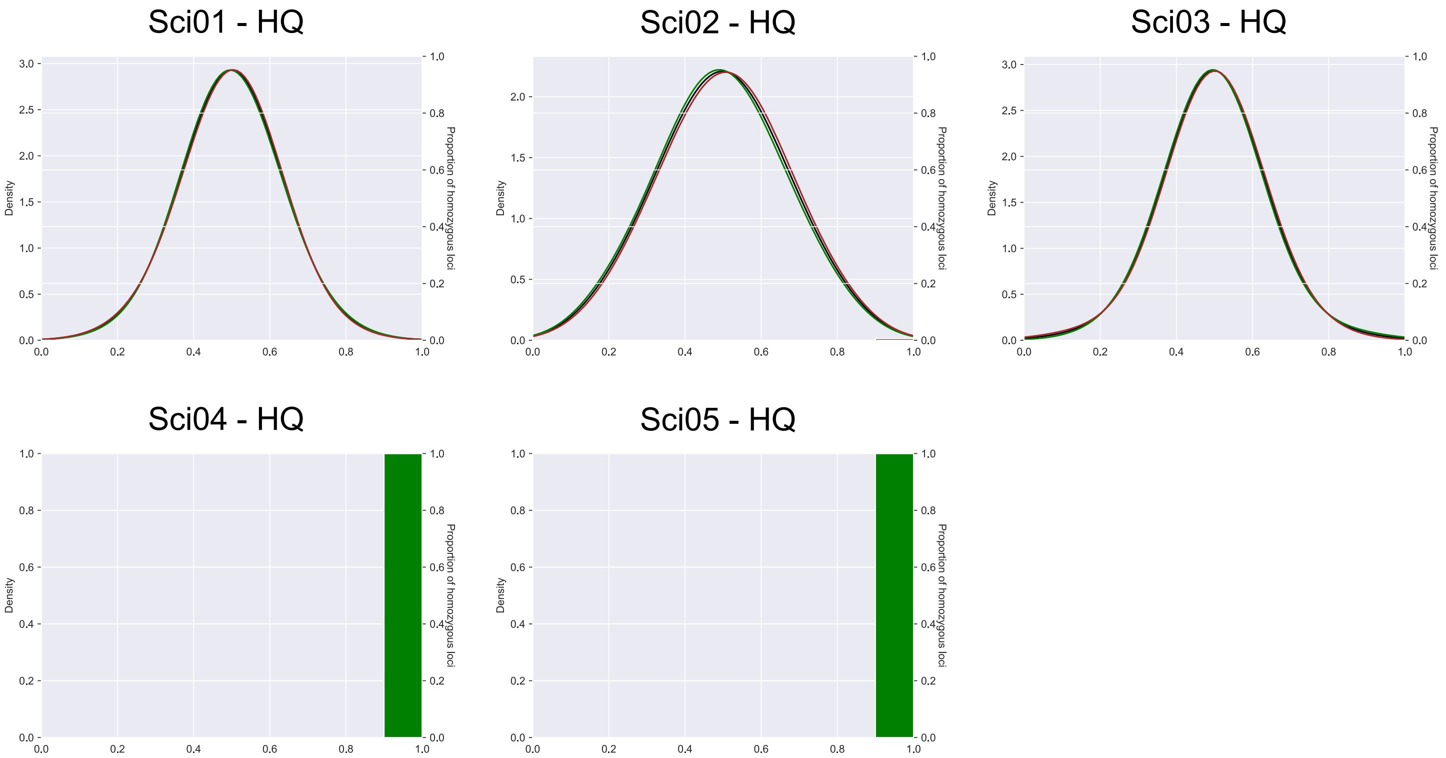
Sci01 - HQ
Sci02 - HQ
Sci03 - HQ
Sci04 - HQ
Sci05 - HQ
Density
Proportion of homozygous loci
0.0
0.2
0.4
0.6
0.8
1.0
0.5
1.5
2.0
2.5
3.0

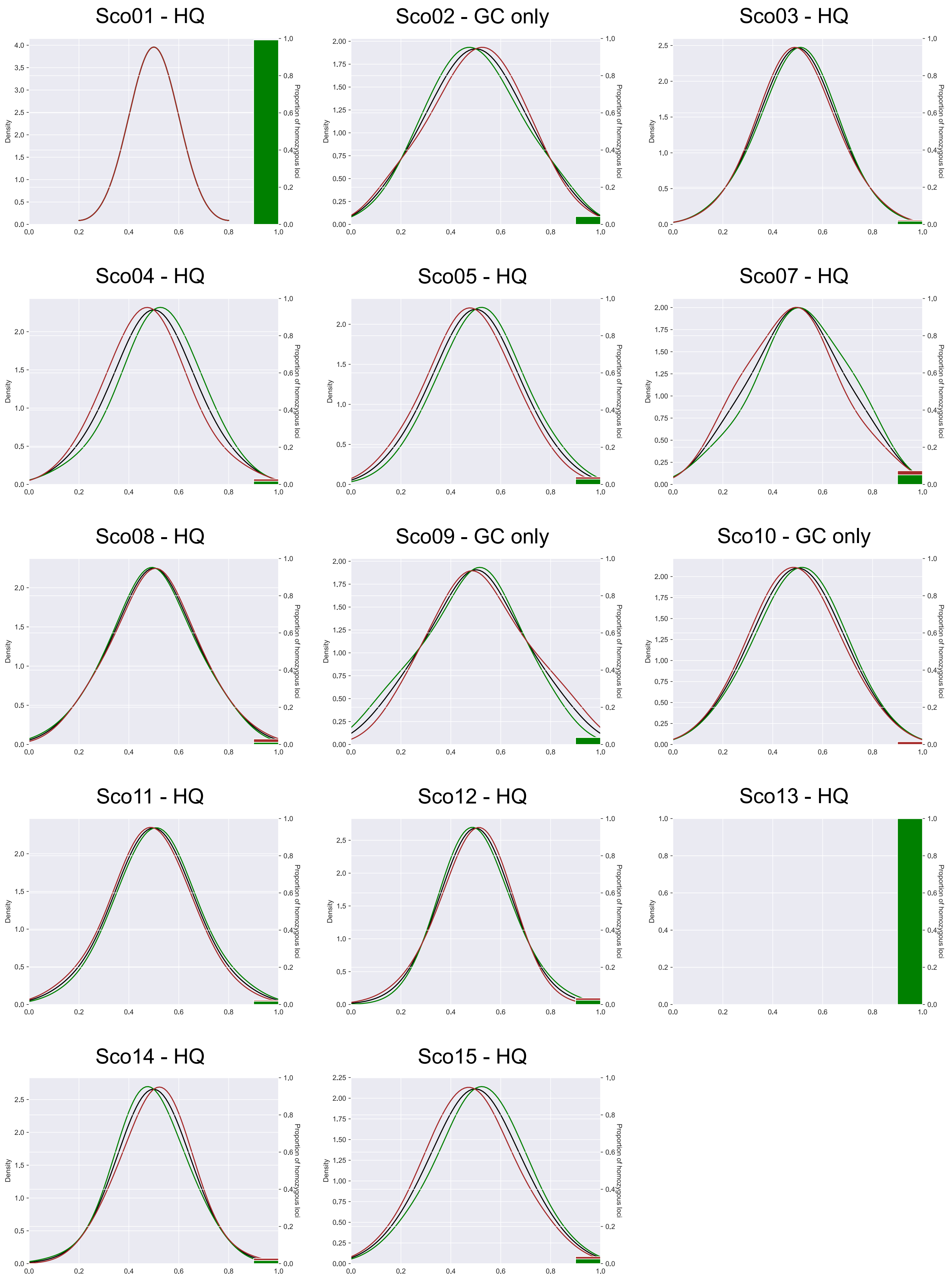

Sco01 - HQ
Sco02 - GC only
Sco03 - HQ
Sco04 - HQ
Sco05 - HQ
Sco07 - HQ
Sco08 - HQ
Sco09 - GC only
Sco10 - GC only
Sco11 - HQ
Sco12 - HQ
Sco13 - HQ
Sco14 - HQ
Sco15 - HQ
Density
Proportion of homozygous loci

Scu01 - GC onlyl

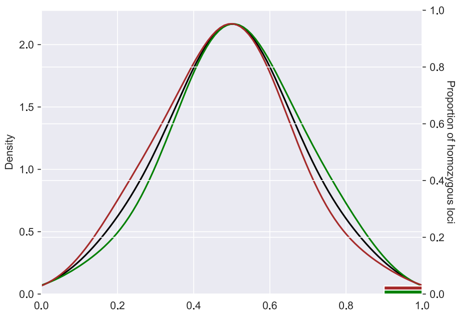

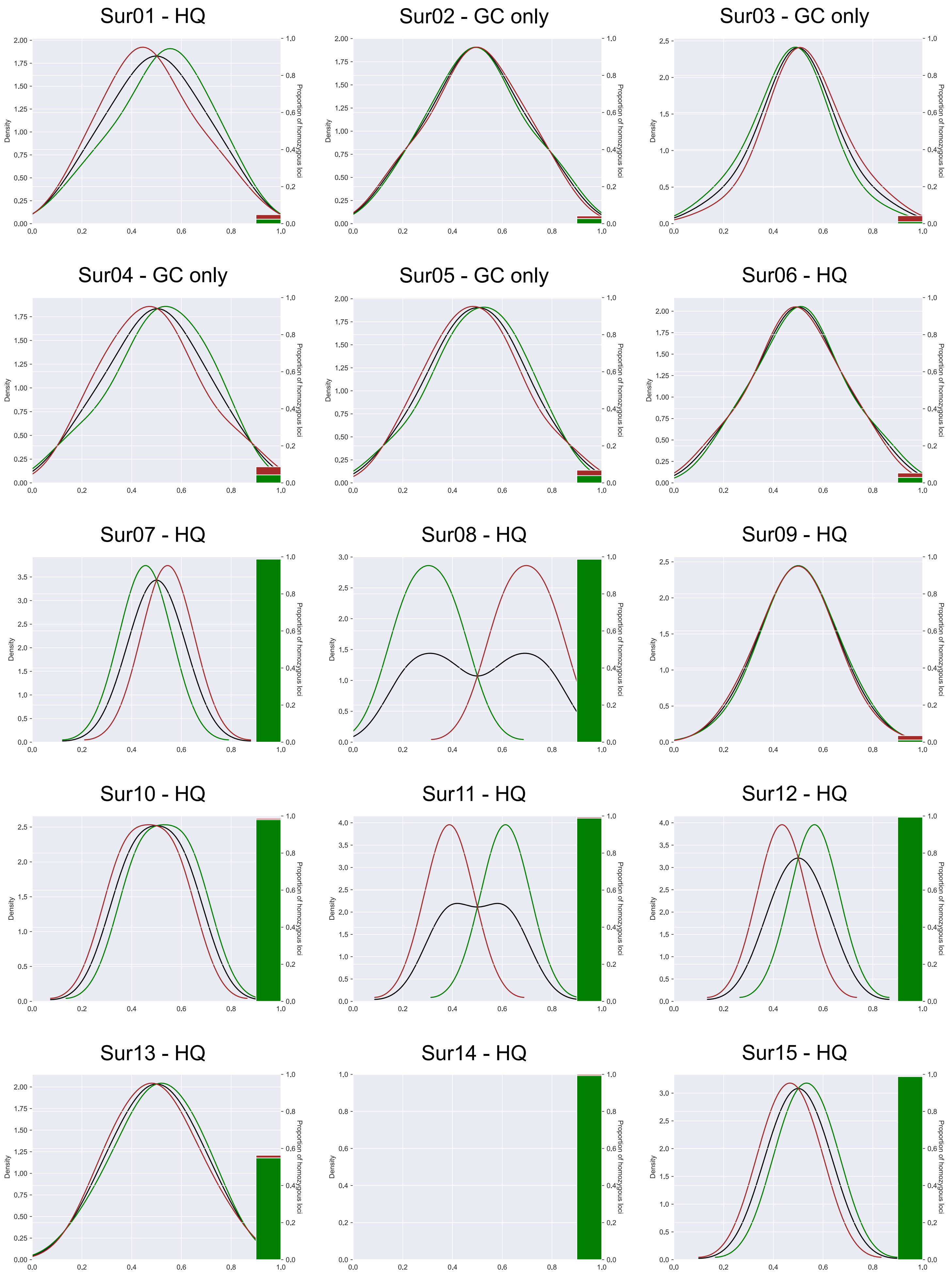
Sur01 - HQ
Sur02 - GC only
Sur03 - GC only
Sur04 - GC only
Sur05 - GC only
Sur06 - HQ
Sur07 - HQ
Sur08 - HQ
Sur09 - HQ
Sur10 - HQ
Sur11 - HQ
Sur12 - HQ
Sur13 - HQ
Sur14 - HQ
Sur15 - HQ
Density
Proportion of homozygous loci

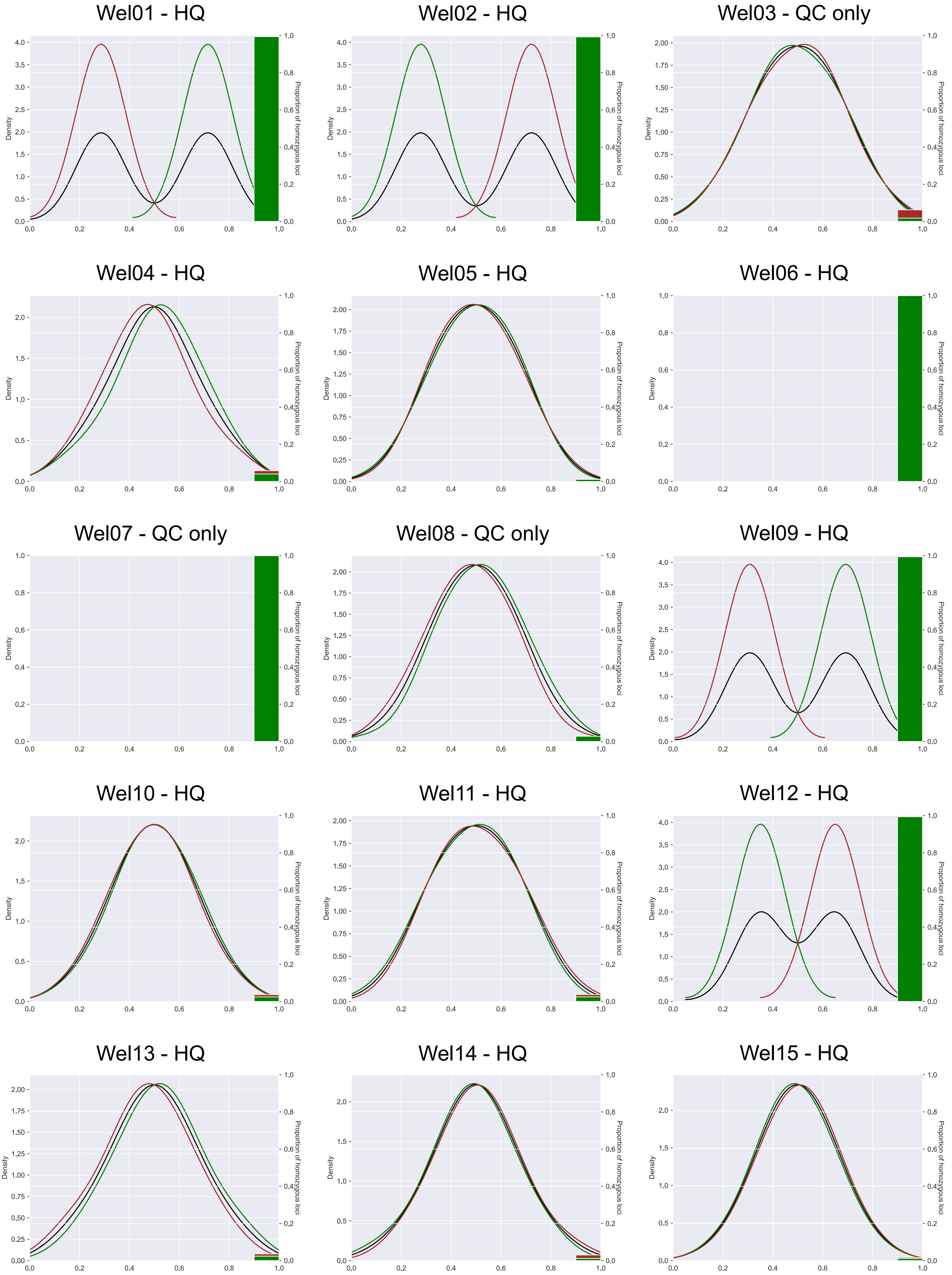
Wel01 - HQ
Wel02 - HQ
Wel03 - QC only
Wel04 - HQ
Wel05 - HQ
Wel06 - HQ
Wel07 - QC only
Wel08 - QC only
Wel09 - HQ
Wel10 - HQ
Wel11 - HQ
Wel12 - HQ
Wel13 - HQ
Wel14 - HQ
Wel15 - HQ
Density
Proportion of homozygous loci

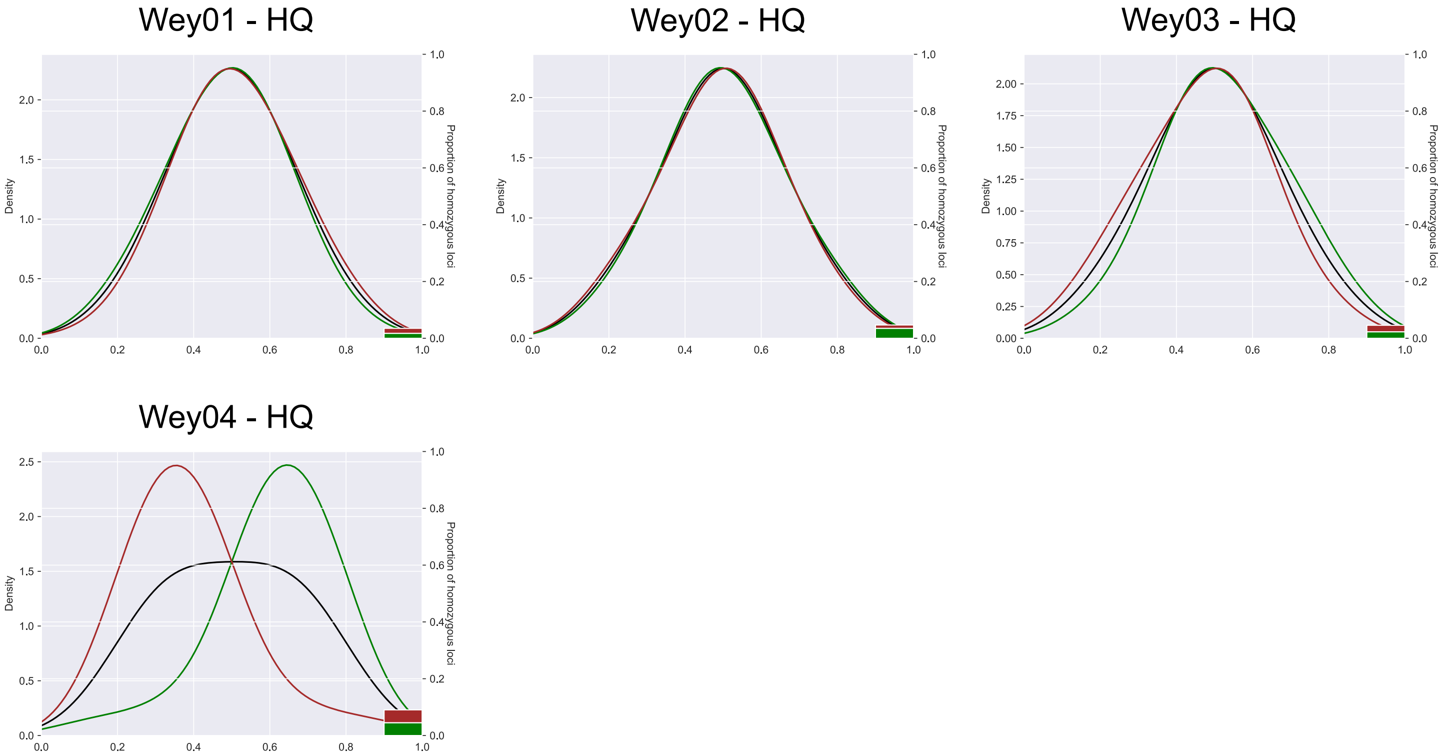
Wey01 - HQ
Wey02 - HQ
Wey03 - HQ
Wey04 - HQ
Density
Proportion of homozygous loci
0,0
0,2
0,4
0,6
0,8
1,0
0.00
0.25
0.50
0.75
1.00
1.25
1.50
1.75
2.00
0.0
0.5
1.0
1.5
2.0
2.5

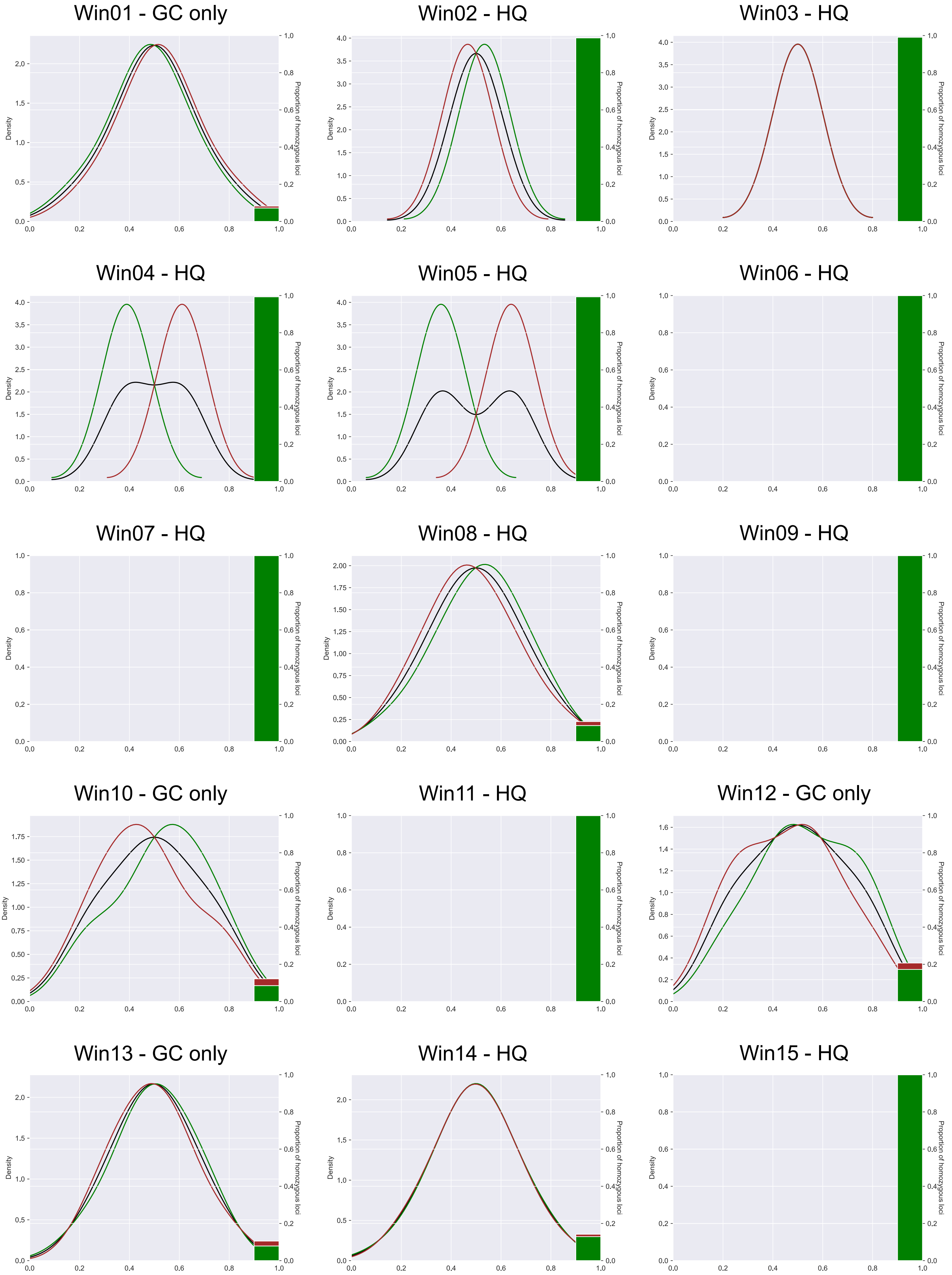
Win01 - GC only
Win02 - HQ
Win03 - HQ
Win04 - HQ
Win05 - HQ
Win06 - HQ
Win07 - HQ
Win08 - HQ
Win09 - HQ
Win10 - GC only
Win11 - HQ
Win12 - GC only
Win13 - GC only
Win14 - HQ
Win15 - HQ
Density
Proportion of homozygous loci
