# Supplementary material for: Genomic basis for an informed conservation management of Pelophylax water frogs in Luxembourg
Source: Ecol Evol. 2022 Apr 11;12(4):e8810. doi: 10.1002/ece3.8810 (PMC9001158; doi:10.1002/ece3.8810)
Supplement: Supplementary file 2 — Figure S2 [file ECE3-12-e8810-s005.pdf]

Figure S2. Chromatograms of the *MND1* sequences. For each individual, two variable sites (position 4 and 26 in the alignment) were analysed for species identification. If repeated sequencing of an individual resulted in conflicting species identifications, both sequences are shown (rep1 and rep2). All sequences are based on DNA from toe clips.

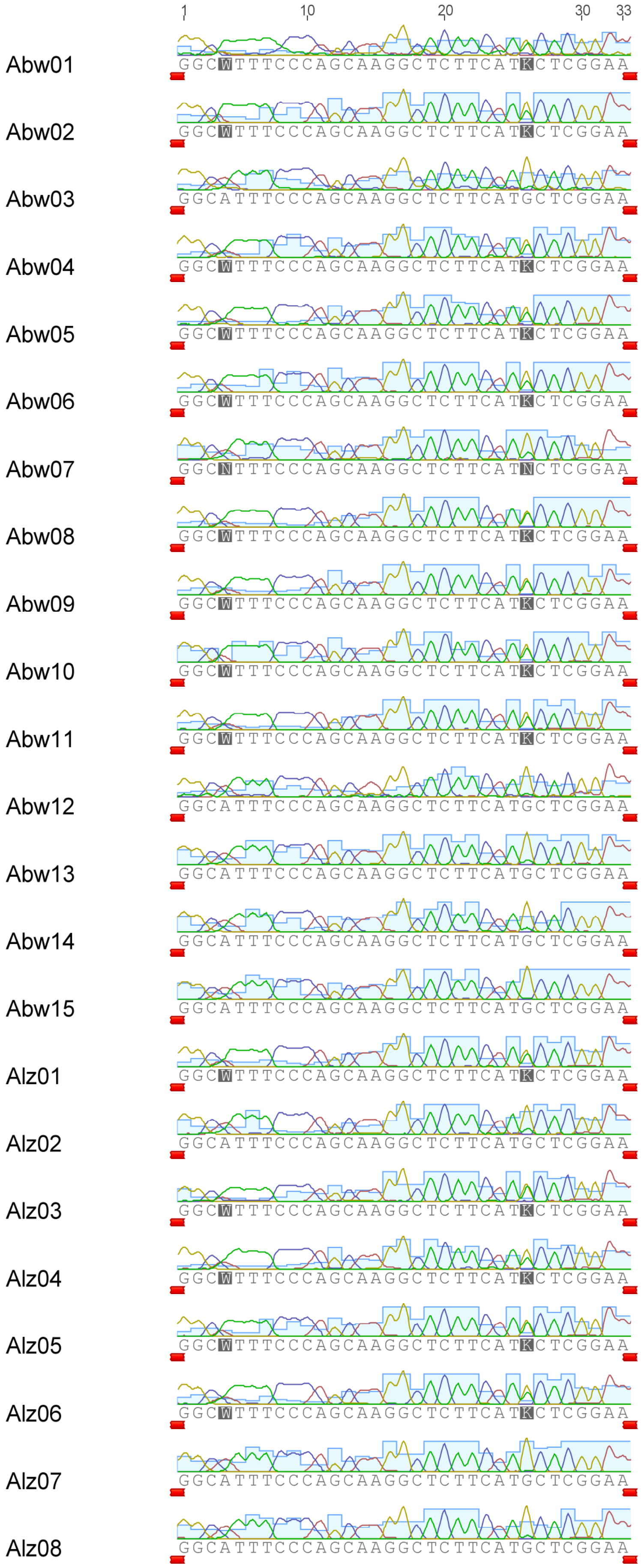
1 10 20 30 33
Abw01 GGCWTTTCCCAGCAAGGCTCTTCATKCTCGGAA
Abw02 GGCWTTTCCCAGCAAGGCTCTTCATKCTCGGAA
Abw03 GGCATTTCCCAGCAAGGCTCTTCATGCTCGGAA
Abw04 GGCWTTTCCCAGCAAGGCTCTTCATKCTCGGAA
Abw05 GGCWTTTCCCAGCAAGGCTCTTCATKCTCGGAA
Abw06 GGCWTTTCCCAGCAAGGCTCTTCATKCTCGGAA
Abw07 GGCNTTTCCCAGCAAGGCTCTTCATNCTCGGAA
Abw08 GGCWTTTCCCAGCAAGGCTCTTCATKCTCGGAA
Abw09 GGCWTTTCCCAGCAAGGCTCTTCATKCTCGGAA
Abw10 GGCWTTTCCCAGCAAGGCTCTTCATKCTCGGAA
Abw11 GGCWTTTCCCAGCAAGGCTCTTCATKCTCGGAA
Abw12 GGCATTTCCCAGCAAGGCTCTTCATGCTCGGAA
Abw13 GGCATTTCCCAGCAAGGCTCTTCATGCTCGGAA
Abw14 GGCATTTCCCAGCAAGGCTCTTCATGCTCGGAA
Abw15 GGCATTTCCCAGCAAGGCTCTTCATGCTCGGAA
Alz01 GGCWTTTCCCAGCAAGGCTCTTCATKCTCGGAA
Alz02 GGCATTTCCCAGCAAGGCTCTTCATGCTCGGAA
Alz03 GGCWTTTCCCAGCAAGGCTCTTCATKCTCGGAA
Alz04 GGCWTTTCCCAGCAAGGCTCTTCATKCTCGGAA
Alz05 GGCWTTTCCCAGCAAGGCTCTTCATKCTCGGAA
Alz06 GGCWTTTCCCAGCAAGGCTCTTCATKCTCGGAA
Alz07 GGCATTTCCCAGCAAGGCTCTTCATGCTCGGAA
Alz08 GGCATTTCCCAGCAAGGCTCTTCATGCTCGGAA

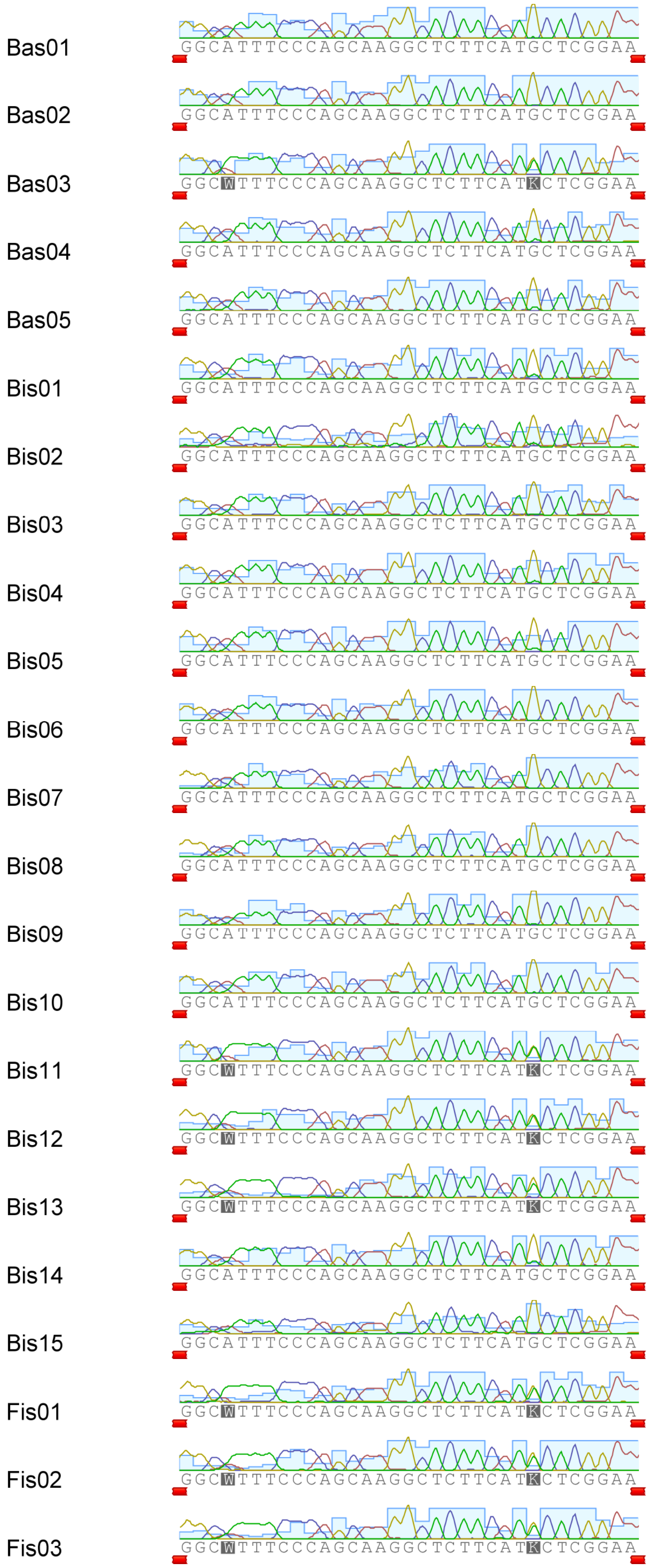
Bas01 GGCATTTCCCAGCAAGGCTCTTCATGCTCGGAA
Bas02 GGCATTTCCCAGCAAGGCTCTTCATGCTCGGAA
Bas03 GGCWTTTCCCAGCAAGGCTCTTCATKCTCGGAA
Bas04 GGCATTTCCCAGCAAGGCTCTTCATGCTCGGAA
Bas05 GGCATTTCCCAGCAAGGCTCTTCATGCTCGGAA
Bis01 GGCATTTCCCAGCAAGGCTCTTCATGCTCGGAA
Bis02 GGCATTTCCCAGCAAGGCTCTTCATGCTCGGAA
Bis03 GGCATTTCCCAGCAAGGCTCTTCATGCTCGGAA
Bis04 GGCATTTCCCAGCAAGGCTCTTCATGCTCGGAA
Bis05 GGCATTTCCCAGCAAGGCTCTTCATGCTCGGAA
Bis06 GGCATTTCCCAGCAAGGCTCTTCATGCTCGGAA
Bis07 GGCATTTCCCAGCAAGGCTCTTCATGCTCGGAA
Bis08 GGCATTTCCCAGCAAGGCTCTTCATGCTCGGAA
Bis09 GGCATTTCCCAGCAAGGCTCTTCATGCTCGGAA
Bis10 GGCATTTCCCAGCAAGGCTCTTCATGCTCGGAA
Bis11 GGCWTTTCCCAGCAAGGCTCTTCATKCTCGGAA
Bis12 GGCWTTTCCCAGCAAGGCTCTTCATKCTCGGAA
Bis13 GGCWTTTCCCAGCAAGGCTCTTCATKCTCGGAA
Bis14 GGCATTTCCCAGCAAGGCTCTTCATGCTCGGAA
Bis15 GGCATTTCCCAGCAAGGCTCTTCATGCTCGGAA
Fis01 GGCWTTTCCCAGCAAGGCTCTTCATKCTCGGAA
Fis02 GGCWTTTCCCAGCAAGGCTCTTCATKCTCGGAA
Fis03 GGCWTTTCCCAGCAAGGCTCTTCATKCTCGGAA

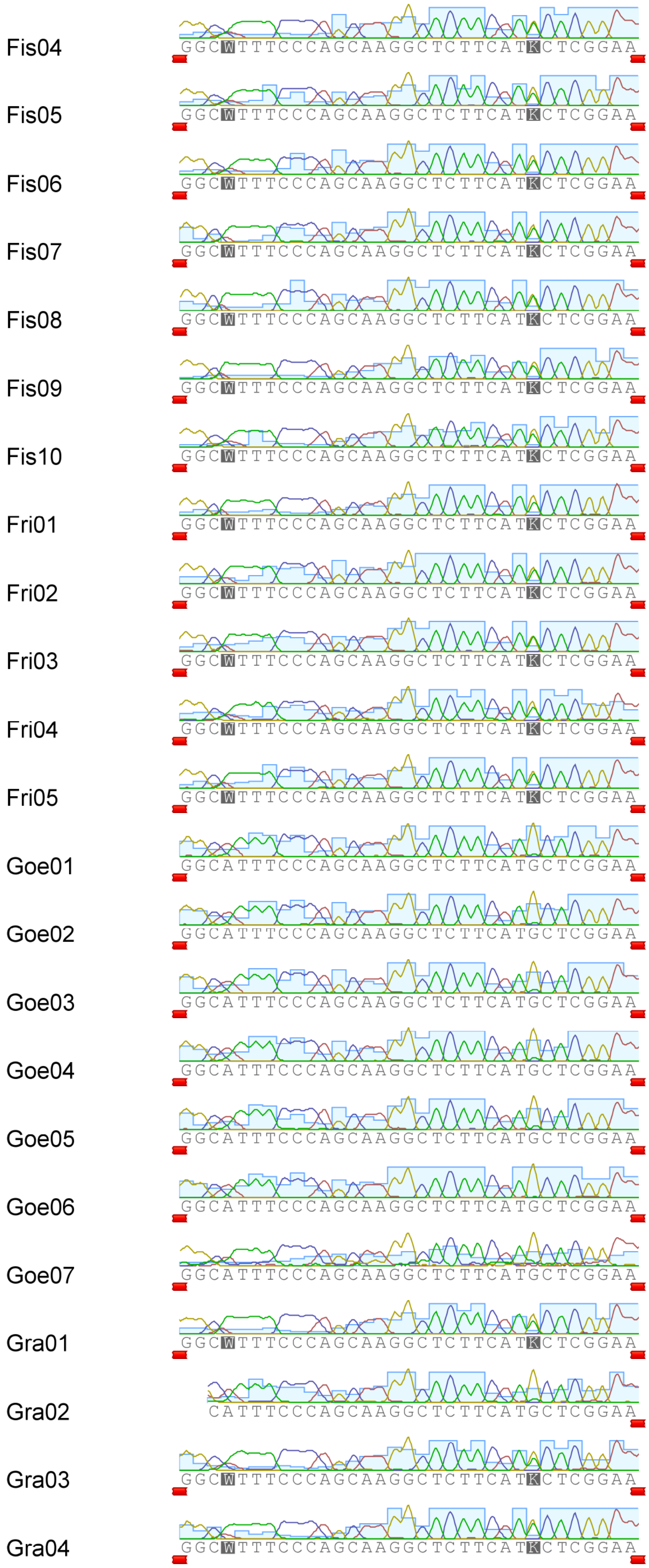
Fis04 GGCWTTTCCCAGCAAGGCTCTTCATKCTCGGAA
Fis05 GGCWTTTCCCAGCAAGGCTCTTCATKCTCGGAA
Fis06 GGCWTTTCCCAGCAAGGCTCTTCATKCTCGGAA
Fis07 GGCWTTTCCCAGCAAGGCTCTTCATKCTCGGAA
Fis08 GGCWTTTCCCAGCAAGGCTCTTCATKCTCGGAA
Fis09 GGCWTTTCCCAGCAAGGCTCTTCATKCTCGGAA
Fis10 GGCWTTTCCCAGCAAGGCTCTTCATKCTCGGAA
Fri01 GGCWTTTCCCAGCAAGGCTCTTCATKCTCGGAA
Fri02 GGCWTTTCCCAGCAAGGCTCTTCATKCTCGGAA
Fri03 GGCWTTTCCCAGCAAGGCTCTTCATKCTCGGAA
Fri04 GGCWTTTCCCAGCAAGGCTCTTCATKCTCGGAA
Fri05 GGCWTTTCCCAGCAAGGCTCTTCATKCTCGGAA
Goe01 GGCATTTCCCAGCAAGGCTCTTCATGCTCGGAA
Goe02 GGCATTTCCCAGCAAGGCTCTTCATGCTCGGAA
Goe03 GGCATTTCCCAGCAAGGCTCTTCATGCTCGGAA
Goe04 GGCATTTCCCAGCAAGGCTCTTCATGCTCGGAA
Goe05 GGCATTTCCCAGCAAGGCTCTTCATGCTCGGAA
Goe06 GGCATTTCCCAGCAAGGCTCTTCATGCTCGGAA
Goe07 GGCATTTCCCAGCAAGGCTCTTCATGCTCGGAA
Gra01 GGCWTTTCCCAGCAAGGCTCTTCATKCTCGGAA
Gra02 CATTTCCCAGCAAGGCTCTTCATGCTCGGAA
Gra03 GGCWTTTCCCAGCAAGGCTCTTCATKCTCGGAA
Gra04 GGCWTTTCCCAGCAAGGCTCTTCATKCTCGGAA

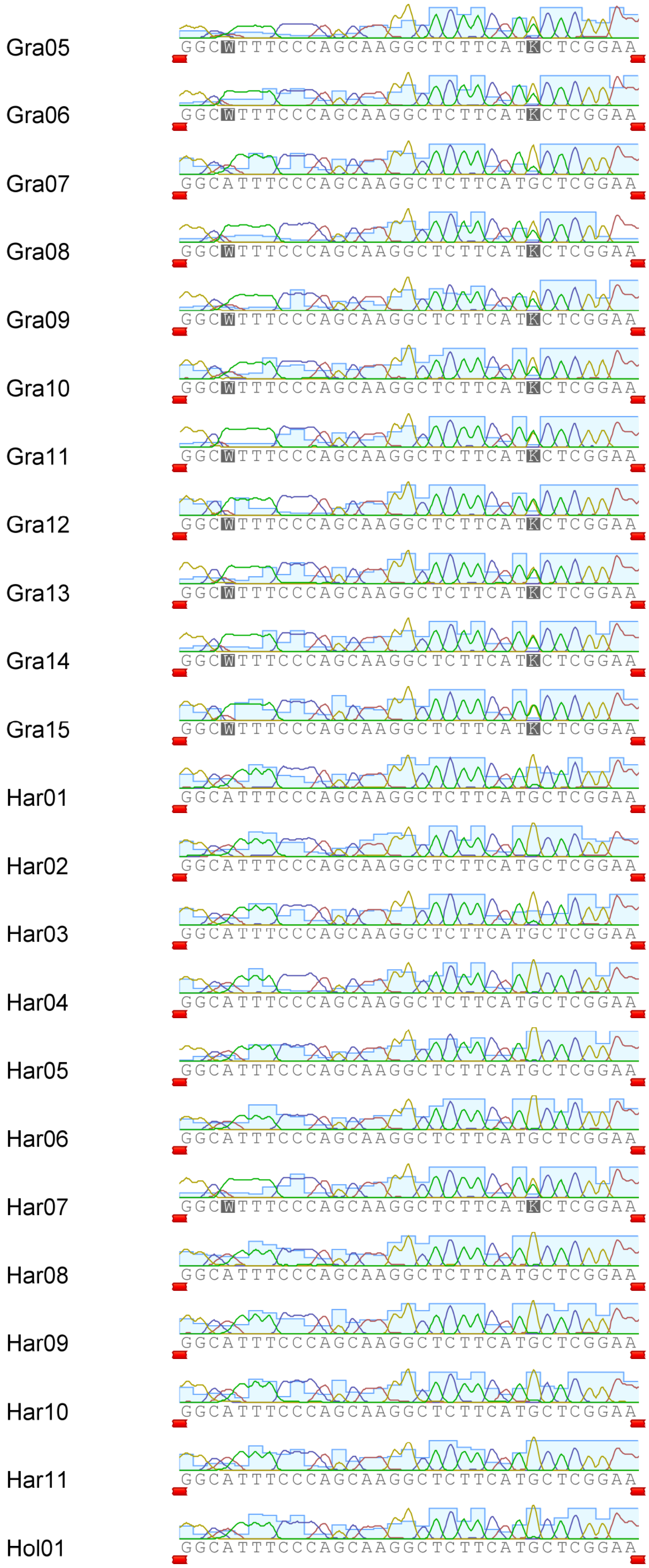
Gra05 GGCWTTTCCCAGCAAGGCTCTTCATKCTCGGAA
Gra06 GGCWTTTCCCAGCAAGGCTCTTCATKCTCGGAA
Gra07 GGCATTTCCCAGCAAGGCTCTTCATGCTCGGAA
Gra08 GGCWTTTCCCAGCAAGGCTCTTCATKCTCGGAA
Gra09 GGCWTTTCCCAGCAAGGCTCTTCATKCTCGGAA
Gra10 GGCWTTTCCCAGCAAGGCTCTTCATKCTCGGAA
Gra11 GGCWTTTCCCAGCAAGGCTCTTCATKCTCGGAA
Gra12 GGCWTTTCCCAGCAAGGCTCTTCATKCTCGGAA
Gra13 GGCWTTTCCCAGCAAGGCTCTTCATKCTCGGAA
Gra14 GGCWTTTCCCAGCAAGGCTCTTCATKCTCGGAA
Gra15 GGCWTTTCCCAGCAAGGCTCTTCATKCTCGGAA
Har01 GGCATTTCCCAGCAAGGCTCTTCATGCTCGGAA
Har02 GGCATTTCCCAGCAAGGCTCTTCATGCTCGGAA
Har03 GGCATTTCCCAGCAAGGCTCTTCATGCTCGGAA
Har04 GGCATTTCCCAGCAAGGCTCTTCATGCTCGGAA
Har05 GGCATTTCCCAGCAAGGCTCTTCATGCTCGGAA
Har06 GGCATTTCCCAGCAAGGCTCTTCATGCTCGGAA
Har07 GGCWTTTCCCAGCAAGGCTCTTCATKCTCGGAA
Har08 GGCATTTCCCAGCAAGGCTCTTCATGCTCGGAA
Har09 GGCATTTCCCAGCAAGGCTCTTCATGCTCGGAA
Har10 GGCATTTCCCAGCAAGGCTCTTCATGCTCGGAA
Har11 GGCATTTCCCAGCAAGGCTCTTCATGCTCGGAA
Hol01 GGCATTTCCCAGCAAGGCTCTTCATGCTCGGAA

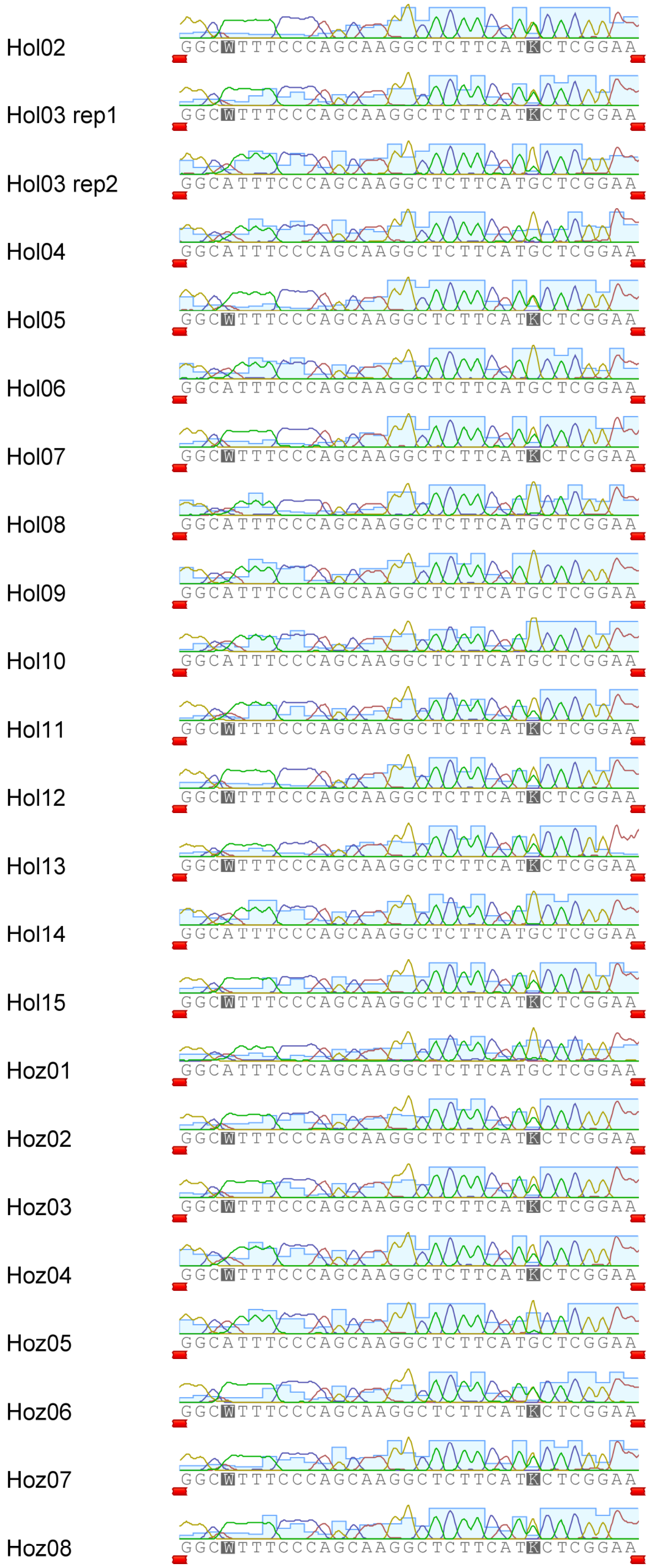
Hol02
GGCWTTTCCCAGCAAGGCTCTTCATKCTCGGAA
Hol03 rep1
GGCWTTTCCCAGCAAGGCTCTTCATKCTCGGAA
Hol03 rep2
GGCATTTCCCAGCAAGGCTCTTCATGCTCGGAA
Hol04
GGCATTTCCCAGCAAGGCTCTTCATGCTCGGAA
Hol05
GGCWTTTCCCAGCAAGGCTCTTCATKCTCGGAA
Hol06
GGCATTTCCCAGCAAGGCTCTTCATGCTCGGAA
Hol07
GGCWTTTCCCAGCAAGGCTCTTCATKCTCGGAA
Hol08
GGCATTTCCCAGCAAGGCTCTTCATGCTCGGAA
Hol09
GGCATTTCCCAGCAAGGCTCTTCATGCTCGGAA
Hol10
GGCATTTCCCAGCAAGGCTCTTCATGCTCGGAA
Hol11
GGCWTTTCCCAGCAAGGCTCTTCATKCTCGGAA
Hol12
GGCWTTTCCCAGCAAGGCTCTTCATKCTCGGAA
Hol13
GGCWTTTCCCAGCAAGGCTCTTCATKCTCGGAA
Hol14
GGCATTTCCCAGCAAGGCTCTTCATGCTCGGAA
Hol15
GGCWTTTCCCAGCAAGGCTCTTCATKCTCGGAA
Hoz01
GGCATTTCCCAGCAAGGCTCTTCATGCTCGGAA
Hoz02
GGCWTTTCCCAGCAAGGCTCTTCATKCTCGGAA
Hoz03
GGCWTTTCCCAGCAAGGCTCTTCATKCTCGGAA
Hoz04
GGCWTTTCCCAGCAAGGCTCTTCATKCTCGGAA
Hoz05
GGCATTTCCCAGCAAGGCTCTTCATGCTCGGAA
Hoz06
GGCWTTTCCCAGCAAGGCTCTTCATKCTCGGAA
Hoz07
GGCWTTTCCCAGCAAGGCTCTTCATKCTCGGAA
Hoz08
GGCWTTTCCCAGCAAGGCTCTTCATKCTCGGAA

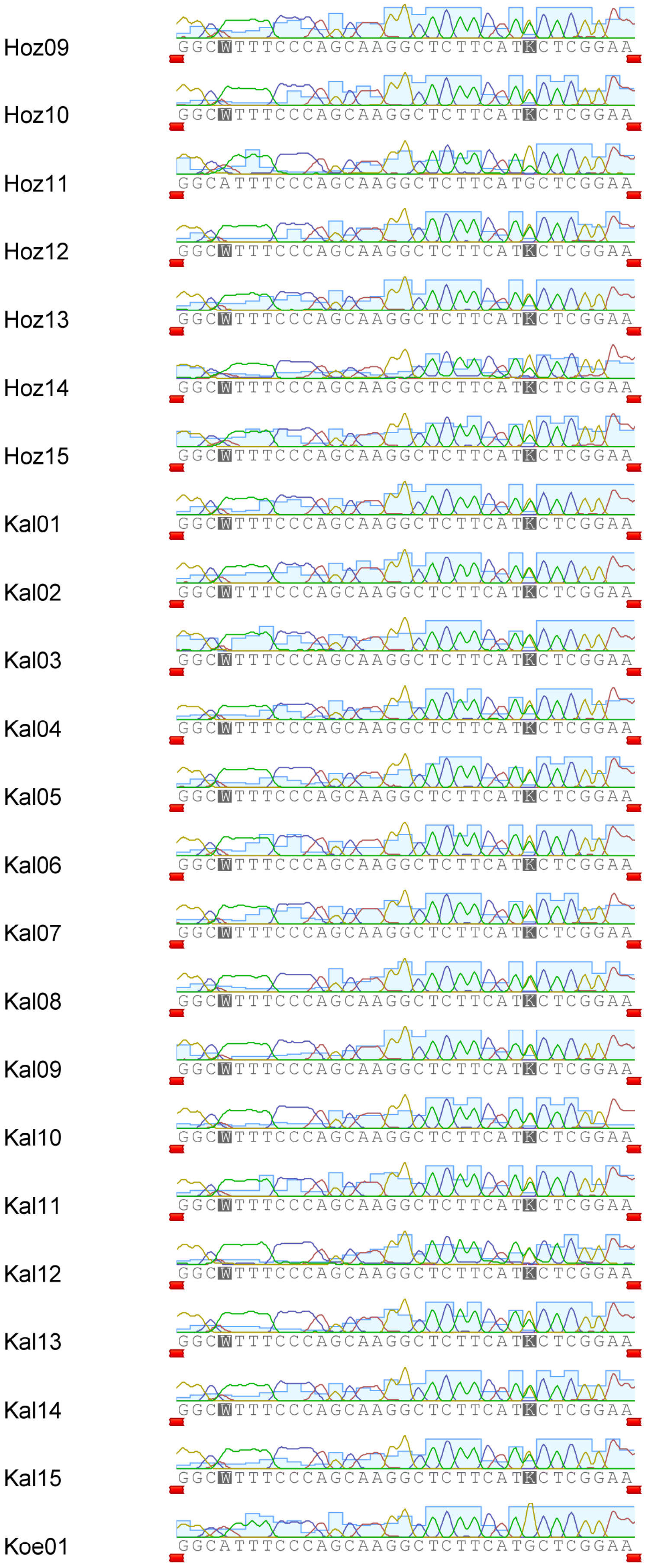
Hoz09 GGCWTTTCCCAGCAAGGCTCTTCATKCTCGGAA
Hoz10 GGCWTTTCCCAGCAAGGCTCTTCATKCTCGGAA
Hoz11 GGCATTTCCCAGCAAGGCTCTTCATGCTCGGAA
Hoz12 GGCWTTTCCCAGCAAGGCTCTTCATKCTCGGAA
Hoz13 GGCWTTTCCCAGCAAGGCTCTTCATKCTCGGAA
Hoz14 GGCWTTTCCCAGCAAGGCTCTTCATKCTCGGAA
Hoz15 GGCWTTTCCCAGCAAGGCTCTTCATKCTCGGAA
Kal01 GGCWTTTCCCAGCAAGGCTCTTCATKCTCGGAA
Kal02 GGCWTTTCCCAGCAAGGCTCTTCATKCTCGGAA
Kal03 GGCWTTTCCCAGCAAGGCTCTTCATKCTCGGAA
Kal04 GGCWTTTCCCAGCAAGGCTCTTCATKCTCGGAA
Kal05 GGCWTTTCCCAGCAAGGCTCTTCATKCTCGGAA
Kal06 GGCWTTTCCCAGCAAGGCTCTTCATKCTCGGAA
Kal07 GGCWTTTCCCAGCAAGGCTCTTCATKCTCGGAA
Kal08 GGCWTTTCCCAGCAAGGCTCTTCATKCTCGGAA
Kal09 GGCWTTTCCCAGCAAGGCTCTTCATKCTCGGAA
Kal10 GGCWTTTCCCAGCAAGGCTCTTCATKCTCGGAA
Kal11 GGCWTTTCCCAGCAAGGCTCTTCATKCTCGGAA
Kal12 GGCWTTTCCCAGCAAGGCTCTTCATKCTCGGAA
Kal13 GGCWTTTCCCAGCAAGGCTCTTCATKCTCGGAA
Kal14 GGCWTTTCCCAGCAAGGCTCTTCATKCTCGGAA
Kal15 GGCWTTTCCCAGCAAGGCTCTTCATKCTCGGAA
Koe01 GGCATTTCCCAGCAAGGCTCTTCATGCTCGGAA

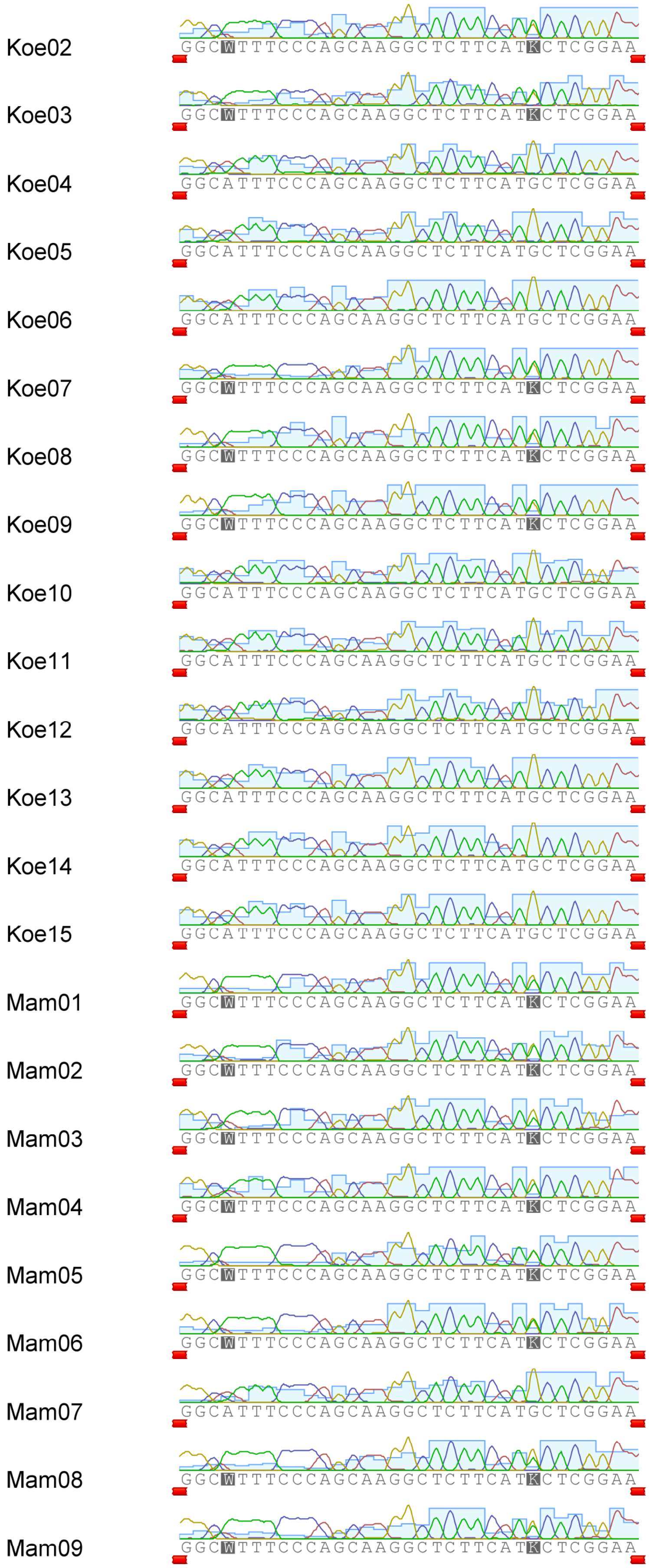
Koe02
GGCWTTTCCCAGCAAGGCTCTTCATKCTCGGAA
Koe03
GGCWTTTCCCAGCAAGGCTCTTCATKCTCGGAA
Koe04
GGCATTTCCCAGCAAGGCTCTTCATGCTCGGAA
Koe05
GGCATTTCCCAGCAAGGCTCTTCATGCTCGGAA
Koe06
GGCATTTCCCAGCAAGGCTCTTCATGCTCGGAA
Koe07
GGCWTTTCCCAGCAAGGCTCTTCATKCTCGGAA
Koe08
GGCWTTTCCCAGCAAGGCTCTTCATKCTCGGAA
Koe09
GGCWTTTCCCAGCAAGGCTCTTCATKCTCGGAA
Koe10
GGCATTTCCCAGCAAGGCTCTTCATGCTCGGAA
Koe11
GGCATTTCCCAGCAAGGCTCTTCATGCTCGGAA
Koe12
GGCATTTCCCAGCAAGGCTCTTCATGCTCGGAA
Koe13
GGCATTTCCCAGCAAGGCTCTTCATGCTCGGAA
Koe14
GGCATTTCCCAGCAAGGCTCTTCATGCTCGGAA
Koe15
GGCATTTCCCAGCAAGGCTCTTCATGCTCGGAA
Mam01
GGCWTTTCCCAGCAAGGCTCTTCATKCTCGGAA
Mam02
GGCWTTTCCCAGCAAGGCTCTTCATKCTCGGAA
Mam03
GGCWTTTCCCAGCAAGGCTCTTCATKCTCGGAA
Mam04
GGCWTTTCCCAGCAAGGCTCTTCATKCTCGGAA
Mam05
GGCWTTTCCCAGCAAGGCTCTTCATKCTCGGAA
Mam06
GGCWTTTCCCAGCAAGGCTCTTCATKCTCGGAA
Mam07
GGCATTTCCCAGCAAGGCTCTTCATGCTCGGAA
Mam08
GGCWTTTCCCAGCAAGGCTCTTCATKCTCGGAA
Mam09
GGCWTTTCCCAGCAAGGCTCTTCATKCTCGGAA

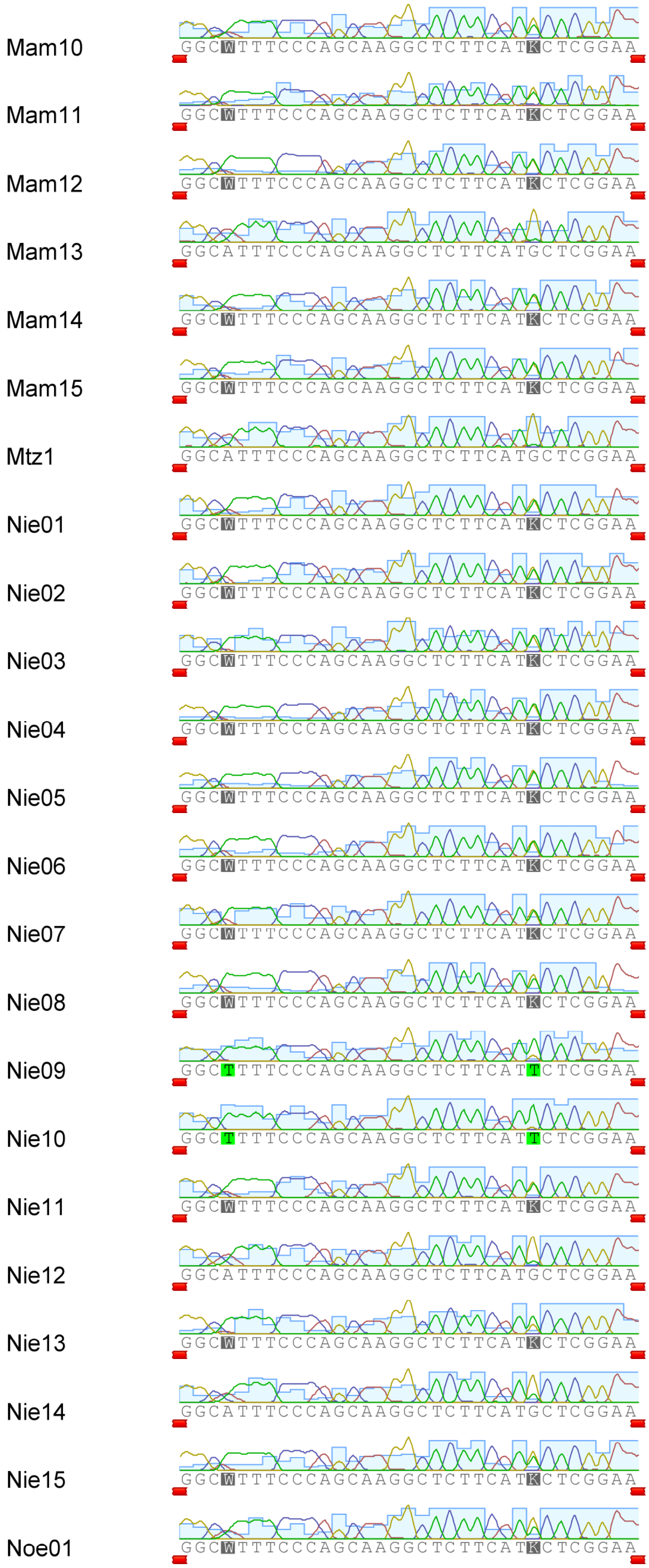
Mam10 GGCWTTTCCCAGCAAGGCTCTTCATKCTCGGAA
Mam11 GGCWTTTCCCAGCAAGGCTCTTCATKCTCGGAA
Mam12 GGCWTTTCCCAGCAAGGCTCTTCATKCTCGGAA
Mam13 GGCATTTCCCAGCAAGGCTCTTCATGCTCGGAA
Mam14 GGCWTTTCCCAGCAAGGCTCTTCATKCTCGGAA
Mam15 GGCWTTTCCCAGCAAGGCTCTTCATKCTCGGAA
Mtz1 GGCATTTCCCAGCAAGGCTCTTCATGCTCGGAA
Nie01 GGCWTTTCCCAGCAAGGCTCTTCATKCTCGGAA
Nie02 GGCWTTTCCCAGCAAGGCTCTTCATKCTCGGAA
Nie03 GGCWTTTCCCAGCAAGGCTCTTCATKCTCGGAA
Nie04 GGCWTTTCCCAGCAAGGCTCTTCATKCTCGGAA
Nie05 GGCWTTTCCCAGCAAGGCTCTTCATKCTCGGAA
Nie06 GGCWTTTCCCAGCAAGGCTCTTCATKCTCGGAA
Nie07 GGCWTTTCCCAGCAAGGCTCTTCATKCTCGGAA
Nie08 GGCWTTTCCCAGCAAGGCTCTTCATKCTCGGAA
Nie09 GGCTTTTCCCAGCAAGGCTCTTCATTCTCGGAA
Nie10 GGCTTTTCCCAGCAAGGCTCTTCATTCTCGGAA
Nie11 GGCWTTTCCCAGCAAGGCTCTTCATKCTCGGAA
Nie12 GGCATTTCCCAGCAAGGCTCTTCATGCTCGGAA
Nie13 GGCWTTTCCCAGCAAGGCTCTTCATKCTCGGAA
Nie14 GGCATTTCCCAGCAAGGCTCTTCATGCTCGGAA
Nie15 GGCWTTTCCCAGCAAGGCTCTTCATKCTCGGAA
Noe01 GGCWTTTCCCAGCAAGGCTCTTCATKCTCGGAA

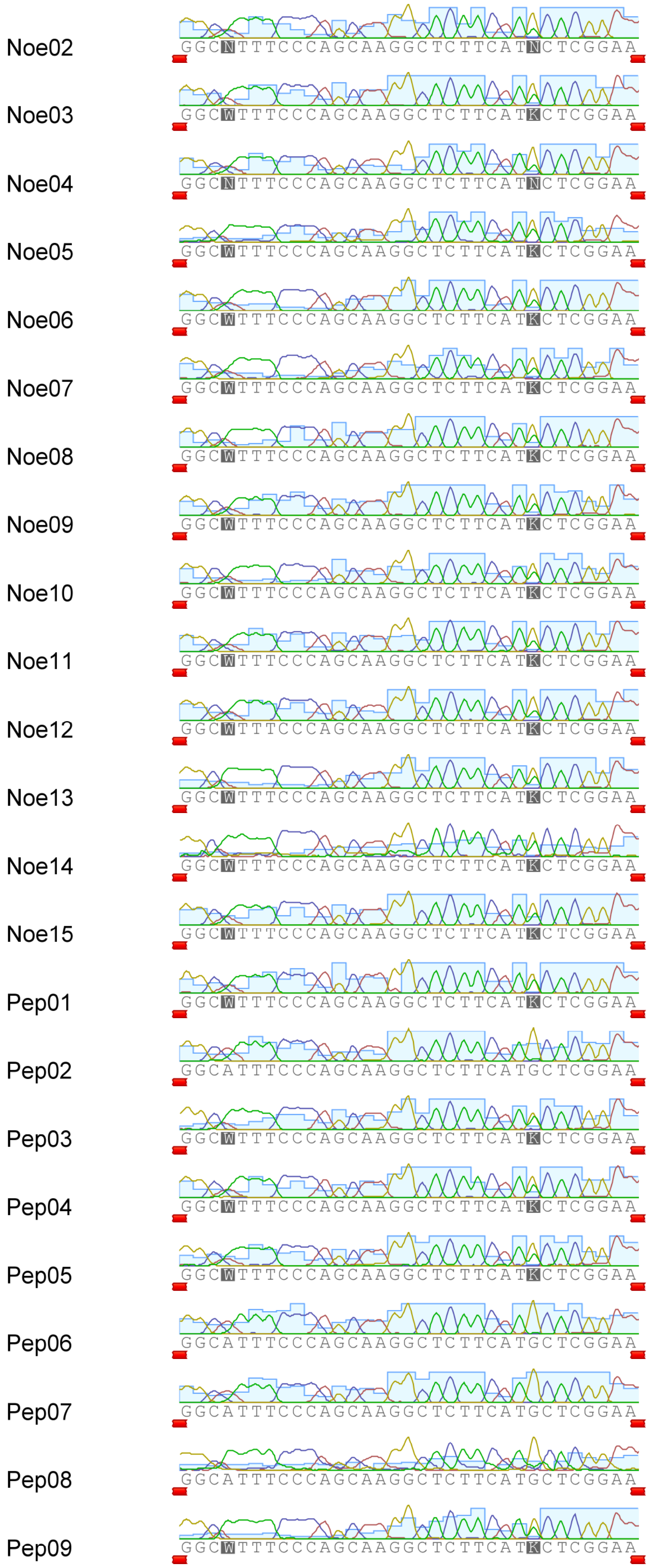
Noe02 GGCNTTTCCCAGCAAGGCTCTTCATNCTCGGAA
Noe03 GGCWTTTCCCAGCAAGGCTCTTCATKCTCGGAA
Noe04 GGCNTTTCCCAGCAAGGCTCTTCATNCTCGGAA
Noe05 GGCWTTTCCCAGCAAGGCTCTTCATKCTCGGAA
Noe06 GGCWTTTCCCAGCAAGGCTCTTCATKCTCGGAA
Noe07 GGCWTTTCCCAGCAAGGCTCTTCATKCTCGGAA
Noe08 GGCWTTTCCCAGCAAGGCTCTTCATKCTCGGAA
Noe09 GGCWTTTCCCAGCAAGGCTCTTCATKCTCGGAA
Noe10 GGCWTTTCCCAGCAAGGCTCTTCATKCTCGGAA
Noe11 GGCWTTTCCCAGCAAGGCTCTTCATKCTCGGAA
Noe12 GGCWTTTCCCAGCAAGGCTCTTCATKCTCGGAA
Noe13 GGCWTTTCCCAGCAAGGCTCTTCATKCTCGGAA
Noe14 GGCWTTTCCCAGCAAGGCTCTTCATKCTCGGAA
Noe15 GGCWTTTCCCAGCAAGGCTCTTCATKCTCGGAA
Pep01 GGCWTTTCCCAGCAAGGCTCTTCATKCTCGGAA
Pep02 GGCATTTCCCAGCAAGGCTCTTCATGCTCGGAA
Pep03 GGCWTTTCCCAGCAAGGCTCTTCATKCTCGGAA
Pep04 GGCWTTTCCCAGCAAGGCTCTTCATKCTCGGAA
Pep05 GGCWTTTCCCAGCAAGGCTCTTCATKCTCGGAA
Pep06 GGCATTTCCCAGCAAGGCTCTTCATGCTCGGAA
Pep07 GGCATTTCCCAGCAAGGCTCTTCATGCTCGGAA
Pep08 GGCATTTCCCAGCAAGGCTCTTCATGCTCGGAA
Pep09 GGCWTTTCCCAGCAAGGCTCTTCATKCTCGGAA

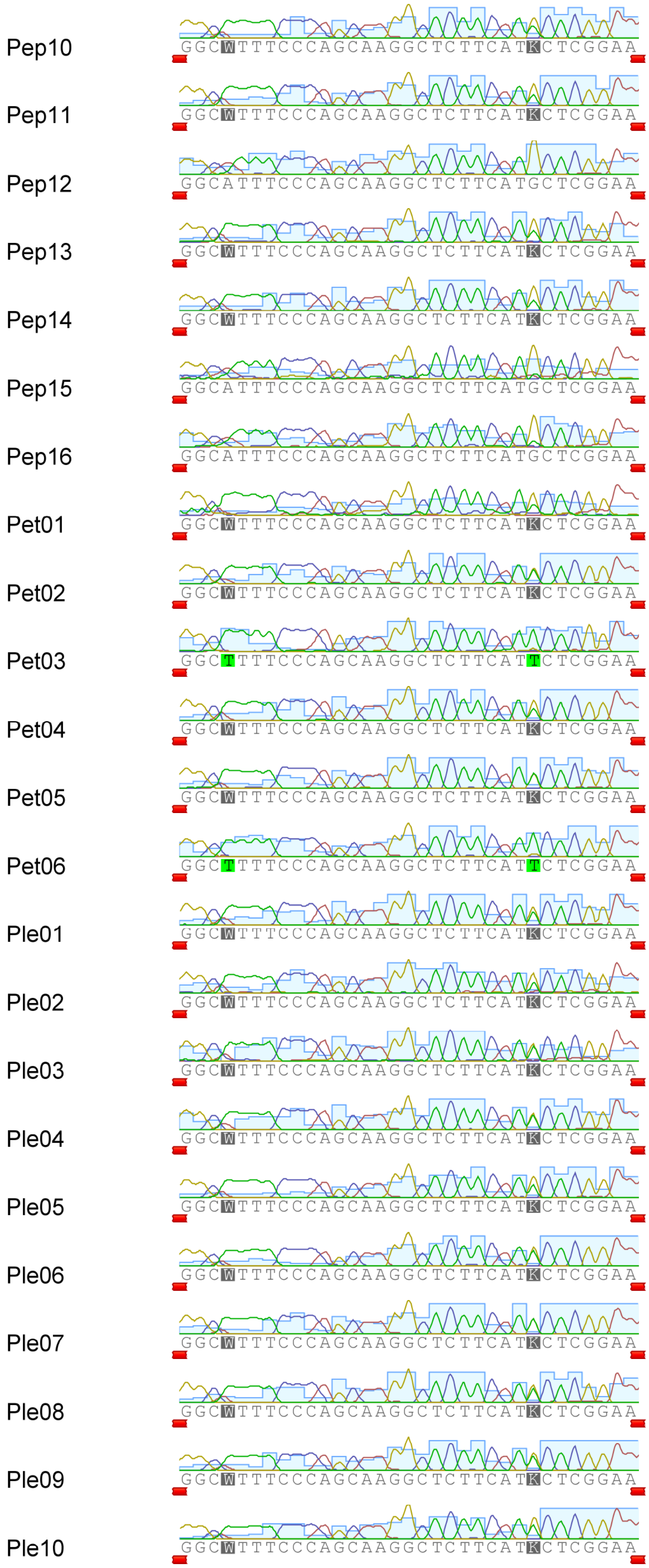
Pep10
GGCWTTTCCCAGCAAGGCTCTTCATKCTCGGAA
Pep11
GGCWTTTCCCAGCAAGGCTCTTCATKCTCGGAA
Pep12
GGCATTTCCCAGCAAGGCTCTTCATGCTCGGAA
Pep13
GGCWTTTCCCAGCAAGGCTCTTCATKCTCGGAA
Pep14
GGCWTTTCCCAGCAAGGCTCTTCATKCTCGGAA
Pep15
GGCATTTCCCAGCAAGGCTCTTCATGCTCGGAA
Pep16
GGCATTTCCCAGCAAGGCTCTTCATGCTCGGAA
Pet01
GGCWTTTCCCAGCAAGGCTCTTCATKCTCGGAA
Pet02
GGCWTTTCCCAGCAAGGCTCTTCATKCTCGGAA
Pet03
GGCTTTTCCCAGCAAGGCTCTTCATTCTCGGAA
Pet04
GGCWTTTCCCAGCAAGGCTCTTCATKCTCGGAA
Pet05
GGCWTTTCCCAGCAAGGCTCTTCATKCTCGGAA
Pet06
GGCTTTTCCCAGCAAGGCTCTTCATTCTCGGAA
Ple01
GGCWTTTCCCAGCAAGGCTCTTCATKCTCGGAA
Ple02
GGCWTTTCCCAGCAAGGCTCTTCATKCTCGGAA
Ple03
GGCWTTTCCCAGCAAGGCTCTTCATKCTCGGAA
Ple04
GGCWTTTCCCAGCAAGGCTCTTCATKCTCGGAA
Ple05
GGCWTTTCCCAGCAAGGCTCTTCATKCTCGGAA
Ple06
GGCWTTTCCCAGCAAGGCTCTTCATKCTCGGAA
Ple07
GGCWTTTCCCAGCAAGGCTCTTCATKCTCGGAA
Ple08
GGCWTTTCCCAGCAAGGCTCTTCATKCTCGGAA
Ple09
GGCWTTTCCCAGCAAGGCTCTTCATKCTCGGAA
Ple10
GGCWTTTCCCAGCAAGGCTCTTCATKCTCGGAA

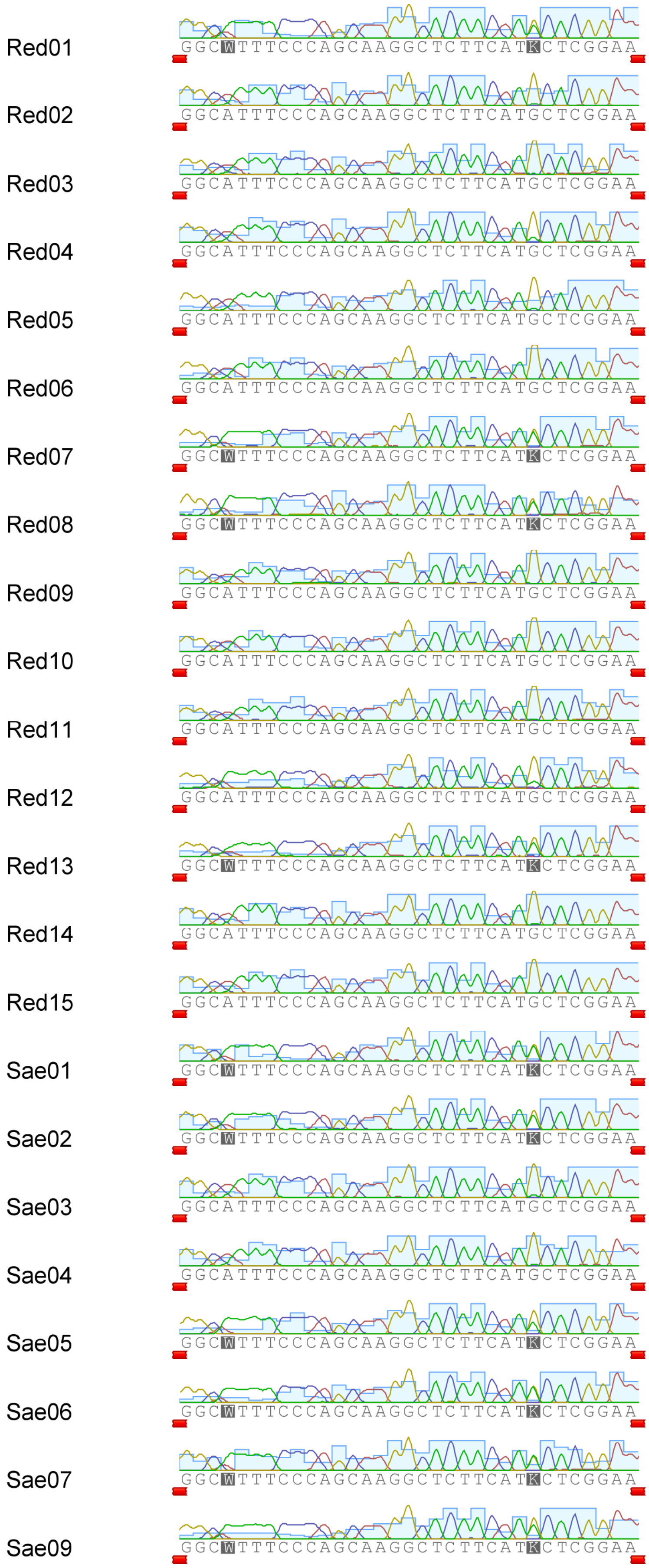
Red01 GGCWTTTCCCAGCAAGGCTCTTCATKCTCGGAA
Red02 GGCATTTCCCAGCAAGGCTCTTCATGCTCGGAA
Red03 GGCATTTCCCAGCAAGGCTCTTCATGCTCGGAA
Red04 GGCATTTCCCAGCAAGGCTCTTCATGCTCGGAA
Red05 GGCATTTCCCAGCAAGGCTCTTCATGCTCGGAA
Red06 GGCATTTCCCAGCAAGGCTCTTCATGCTCGGAA
Red07 GGCWTTTCCCAGCAAGGCTCTTCATKCTCGGAA
Red08 GGCWTTTCCCAGCAAGGCTCTTCATKCTCGGAA
Red09 GGCATTTCCCAGCAAGGCTCTTCATGCTCGGAA
Red10 GGCATTTCCCAGCAAGGCTCTTCATGCTCGGAA
Red11 GGCATTTCCCAGCAAGGCTCTTCATGCTCGGAA
Red12 GGCATTTCCCAGCAAGGCTCTTCATGCTCGGAA
Red13 GGCWTTTCCCAGCAAGGCTCTTCATKCTCGGAA
Red14 GGCATTTCCCAGCAAGGCTCTTCATGCTCGGAA
Red15 GGCATTTCCCAGCAAGGCTCTTCATGCTCGGAA
Sae01 GGCWTTTCCCAGCAAGGCTCTTCATKCTCGGAA
Sae02 GGCWTTTCCCAGCAAGGCTCTTCATKCTCGGAA
Sae03 GGCATTTCCCAGCAAGGCTCTTCATGCTCGGAA
Sae04 GGCATTTCCCAGCAAGGCTCTTCATGCTCGGAA
Sae05 GGCWTTTCCCAGCAAGGCTCTTCATKCTCGGAA
Sae06 GGCWTTTCCCAGCAAGGCTCTTCATKCTCGGAA
Sae07 GGCWTTTCCCAGCAAGGCTCTTCATKCTCGGAA
Sae09 GGCWTTTCCCAGCAAGGCTCTTCATKCTCGGAA

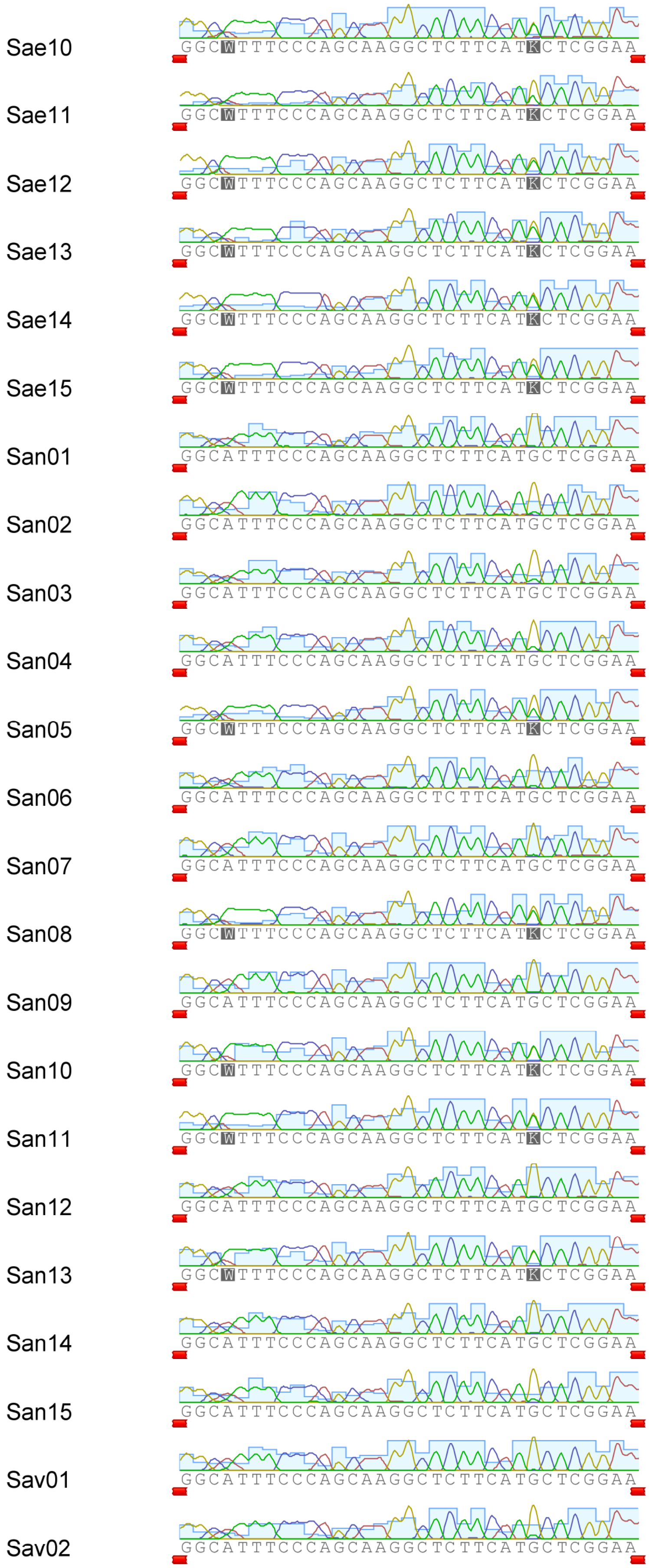
Sae10 GGCWTTTCCCAGCAAGGCTCTTCATKCTCGGAA
Sae11 GGCWTTTCCCAGCAAGGCTCTTCATKCTCGGAA
Sae12 GGCWTTTCCCAGCAAGGCTCTTCATKCTCGGAA
Sae13 GGCWTTTCCCAGCAAGGCTCTTCATKCTCGGAA
Sae14 GGCWTTTCCCAGCAAGGCTCTTCATKCTCGGAA
Sae15 GGCWTTTCCCAGCAAGGCTCTTCATKCTCGGAA
San01 GGCATTTCCCAGCAAGGCTCTTCATGCTCGGAA
San02 GGCATTTCCCAGCAAGGCTCTTCATGCTCGGAA
San03 GGCATTTCCCAGCAAGGCTCTTCATGCTCGGAA
San04 GGCATTTCCCAGCAAGGCTCTTCATGCTCGGAA
San05 GGCWTTTCCCAGCAAGGCTCTTCATKCTCGGAA
San06 GGCATTTCCCAGCAAGGCTCTTCATGCTCGGAA
San07 GGCATTTCCCAGCAAGGCTCTTCATGCTCGGAA
San08 GGCWTTTCCCAGCAAGGCTCTTCATKCTCGGAA
San09 GGCATTTCCCAGCAAGGCTCTTCATGCTCGGAA
San10 GGCWTTTCCCAGCAAGGCTCTTCATKCTCGGAA
San11 GGCWTTTCCCAGCAAGGCTCTTCATKCTCGGAA
San12 GGCATTTCCCAGCAAGGCTCTTCATGCTCGGAA
San13 GGCWTTTCCCAGCAAGGCTCTTCATKCTCGGAA
San14 GGCATTTCCCAGCAAGGCTCTTCATGCTCGGAA
San15 GGCATTTCCCAGCAAGGCTCTTCATGCTCGGAA
Sav01 GGCATTTCCCAGCAAGGCTCTTCATGCTCGGAA
Sav02 GGCATTTCCCAGCAAGGCTCTTCATGCTCGGAA

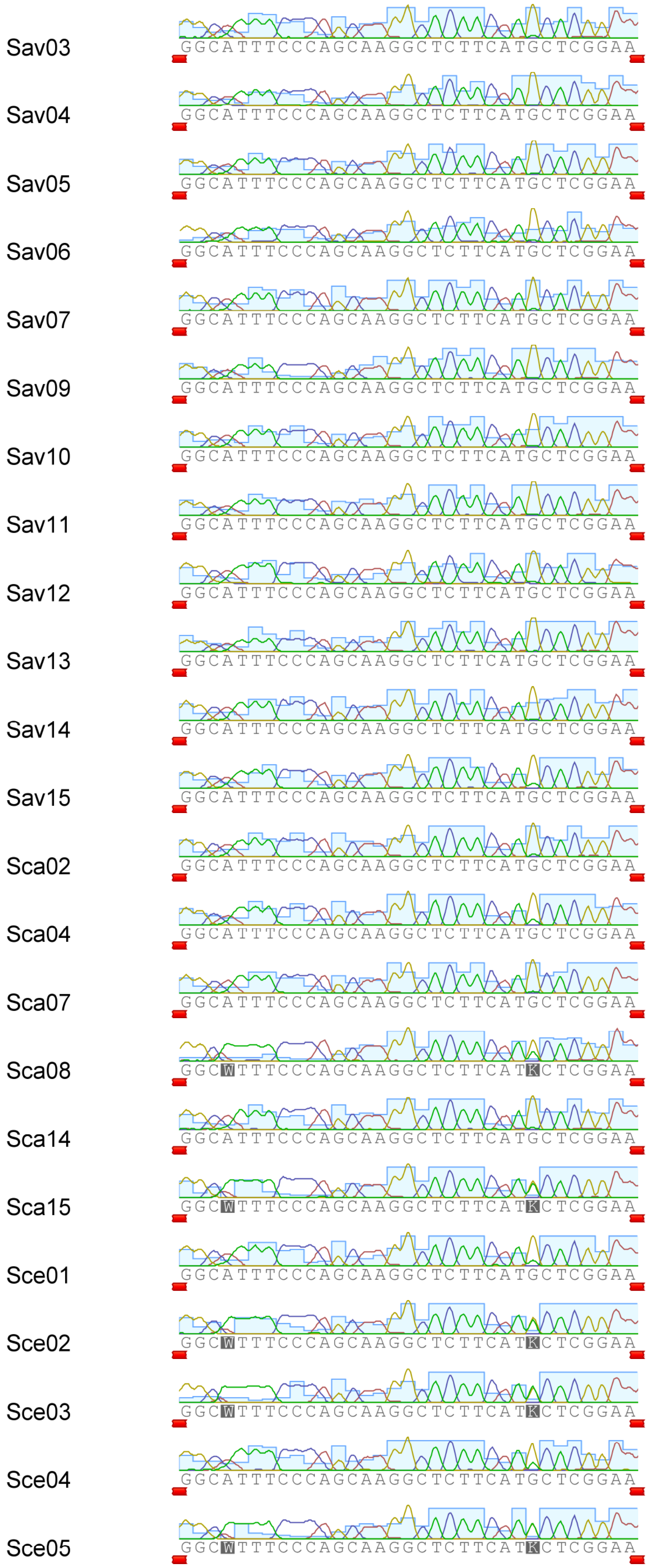
Sav03
GGCATTTCCCAGCAAGGCTCTTCATGCTCGGAA
Sav04
GGCATTTCCCAGCAAGGCTCTTCATGCTCGGAA
Sav05
GGCATTTCCCAGCAAGGCTCTTCATGCTCGGAA
Sav06
GGCATTTCCCAGCAAGGCTCTTCATGCTCGGAA
Sav07
GGCATTTCCCAGCAAGGCTCTTCATGCTCGGAA
Sav09
GGCATTTCCCAGCAAGGCTCTTCATGCTCGGAA
Sav10
GGCATTTCCCAGCAAGGCTCTTCATGCTCGGAA
Sav11
GGCATTTCCCAGCAAGGCTCTTCATGCTCGGAA
Sav12
GGCATTTCCCAGCAAGGCTCTTCATGCTCGGAA
Sav13
GGCATTTCCCAGCAAGGCTCTTCATGCTCGGAA
Sav14
GGCATTTCCCAGCAAGGCTCTTCATGCTCGGAA
Sav15
GGCATTTCCCAGCAAGGCTCTTCATGCTCGGAA
Sca02
GGCATTTCCCAGCAAGGCTCTTCATGCTCGGAA
Sca04
GGCATTTCCCAGCAAGGCTCTTCATGCTCGGAA
Sca07
GGCATTTCCCAGCAAGGCTCTTCATGCTCGGAA
Sca08
GGCWTTTCCCAGCAAGGCTCTTCATKCTCGGAA
Sca14
GGCATTTCCCAGCAAGGCTCTTCATGCTCGGAA
Sca15
GGCWTTTCCCAGCAAGGCTCTTCATKCTCGGAA
Sce01
GGCATTTCCCAGCAAGGCTCTTCATGCTCGGAA
Sce02
GGCWTTTCCCAGCAAGGCTCTTCATKCTCGGAA
Sce03
GGCWTTTCCCAGCAAGGCTCTTCATKCTCGGAA
Sce04
GGCATTTCCCAGCAAGGCTCTTCATGCTCGGAA
Sce05
GGCWTTTCCCAGCAAGGCTCTTCATKCTCGGAA

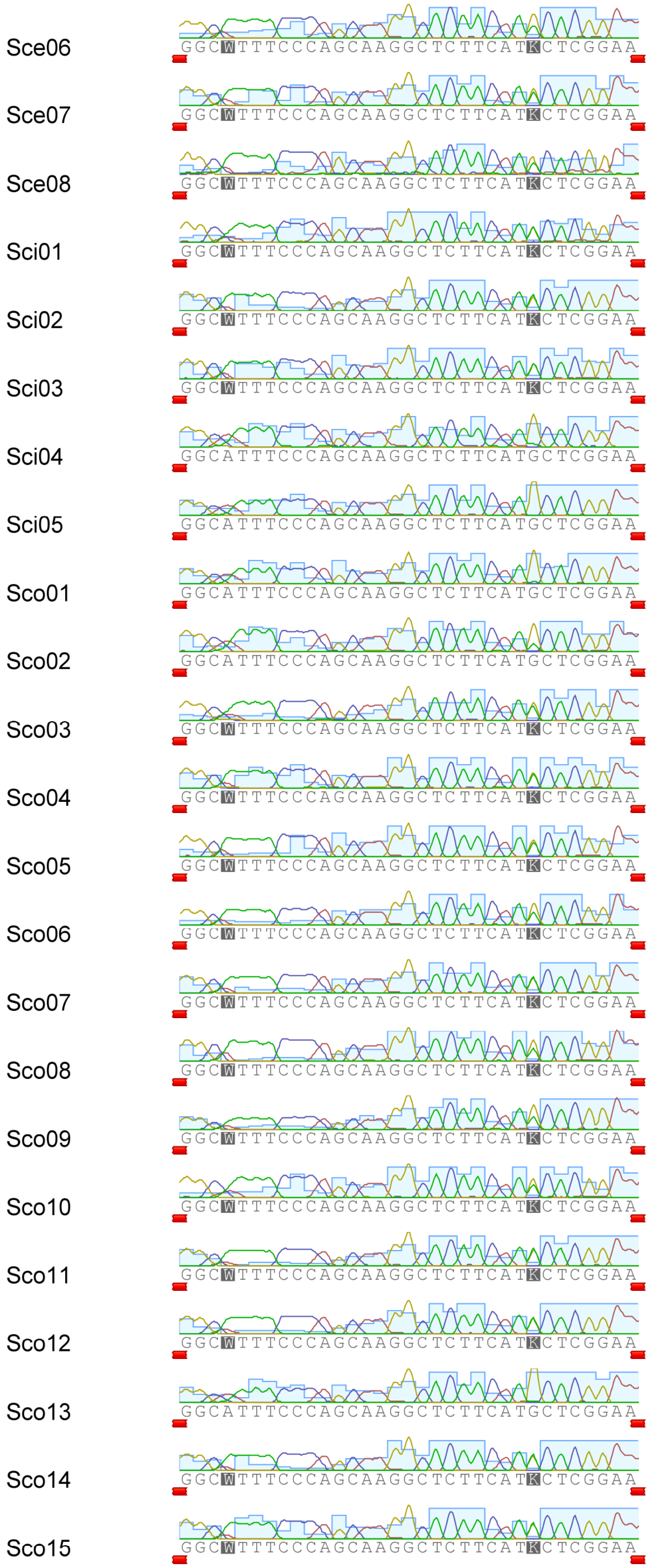

Sce06
GGCWTTTCCCAGCAAGGCTCTTCATKCTCGGAA
Sce07
GGCWTTTCCCAGCAAGGCTCTTCATKCTCGGAA
Sce08
GGCWTTTCCCAGCAAGGCTCTTCATKCTCGGAA
Sci01
GGCWTTTCCCAGCAAGGCTCTTCATKCTCGGAA
Sci02
GGCWTTTCCCAGCAAGGCTCTTCATKCTCGGAA
Sci03
GGCWTTTCCCAGCAAGGCTCTTCATKCTCGGAA
Sci04
GGCATTTCCCAGCAAGGCTCTTCATGCTCGGAA
Sci05
GGCATTTCCCAGCAAGGCTCTTCATGCTCGGAA
Sco01
GGCATTTCCCAGCAAGGCTCTTCATGCTCGGAA
Sco02
GGCATTTCCCAGCAAGGCTCTTCATGCTCGGAA
Sco03
GGCWTTTCCCAGCAAGGCTCTTCATKCTCGGAA
Sco04
GGCWTTTCCCAGCAAGGCTCTTCATKCTCGGAA
Sco05
GGCWTTTCCCAGCAAGGCTCTTCATKCTCGGAA
Sco06
GGCWTTTCCCAGCAAGGCTCTTCATKCTCGGAA
Sco07
GGCWTTTCCCAGCAAGGCTCTTCATKCTCGGAA
Sco08
GGCWTTTCCCAGCAAGGCTCTTCATKCTCGGAA
Sco09
GGCWTTTCCCAGCAAGGCTCTTCATKCTCGGAA
Sco10
GGCWTTTCCCAGCAAGGCTCTTCATKCTCGGAA
Sco11
GGCWTTTCCCAGCAAGGCTCTTCATKCTCGGAA
Sco12
GGCWTTTCCCAGCAAGGCTCTTCATKCTCGGAA
Sco13
GGCATTTCCCAGCAAGGCTCTTCATGCTCGGAA
Sco14
GGCWTTTCCCAGCAAGGCTCTTCATKCTCGGAA
Sco15
GGCWTTTCCCAGCAAGGCTCTTCATKCTCGGAA

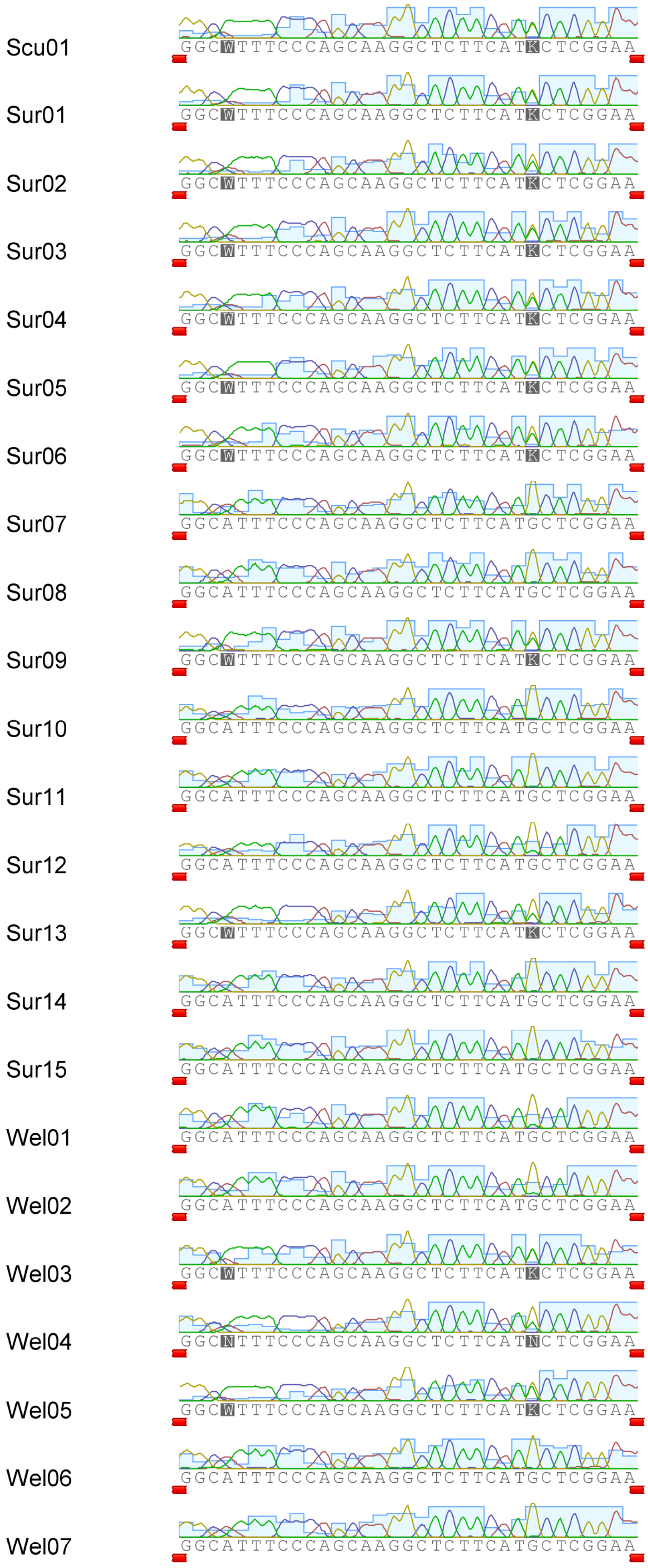
Scu01 GGCWTTTCCCAGCAAGGCTCTTCATKCTCGGAA
Sur01 GGCWTTTCCCAGCAAGGCTCTTCATKCTCGGAA
Sur02 GGCWTTTCCCAGCAAGGCTCTTCATKCTCGGAA
Sur03 GGCWTTTCCCAGCAAGGCTCTTCATKCTCGGAA
Sur04 GGCWTTTCCCAGCAAGGCTCTTCATKCTCGGAA
Sur05 GGCWTTTCCCAGCAAGGCTCTTCATKCTCGGAA
Sur06 GGCWTTTCCCAGCAAGGCTCTTCATKCTCGGAA
Sur07 GGCATTTCCCAGCAAGGCTCTTCATGCTCGGAA
Sur08 GGCATTTCCCAGCAAGGCTCTTCATGCTCGGAA
Sur09 GGCWTTTCCCAGCAAGGCTCTTCATKCTCGGAA
Sur10 GGCATTTCCCAGCAAGGCTCTTCATGCTCGGAA
Sur11 GGCATTTCCCAGCAAGGCTCTTCATGCTCGGAA
Sur12 GGCATTTCCCAGCAAGGCTCTTCATGCTCGGAA
Sur13 GGCWTTTCCCAGCAAGGCTCTTCATKCTCGGAA
Sur14 GGCATTTCCCAGCAAGGCTCTTCATGCTCGGAA
Sur15 GGCATTTCCCAGCAAGGCTCTTCATGCTCGGAA
Wel01 GGCATTTCCCAGCAAGGCTCTTCATGCTCGGAA
Wel02 GGCATTTCCCAGCAAGGCTCTTCATGCTCGGAA
Wel03 GGCWTTTCCCAGCAAGGCTCTTCATKCTCGGAA
Wel04 GGCNTTTCCCAGCAAGGCTCTTCATNCTCGGAA
Wel05 GGCWTTTCCCAGCAAGGCTCTTCATKCTCGGAA
Wel06 GGCATTTCCCAGCAAGGCTCTTCATGCTCGGAA
Wel07 GGCATTTCCCAGCAAGGCTCTTCATGCTCGGAA

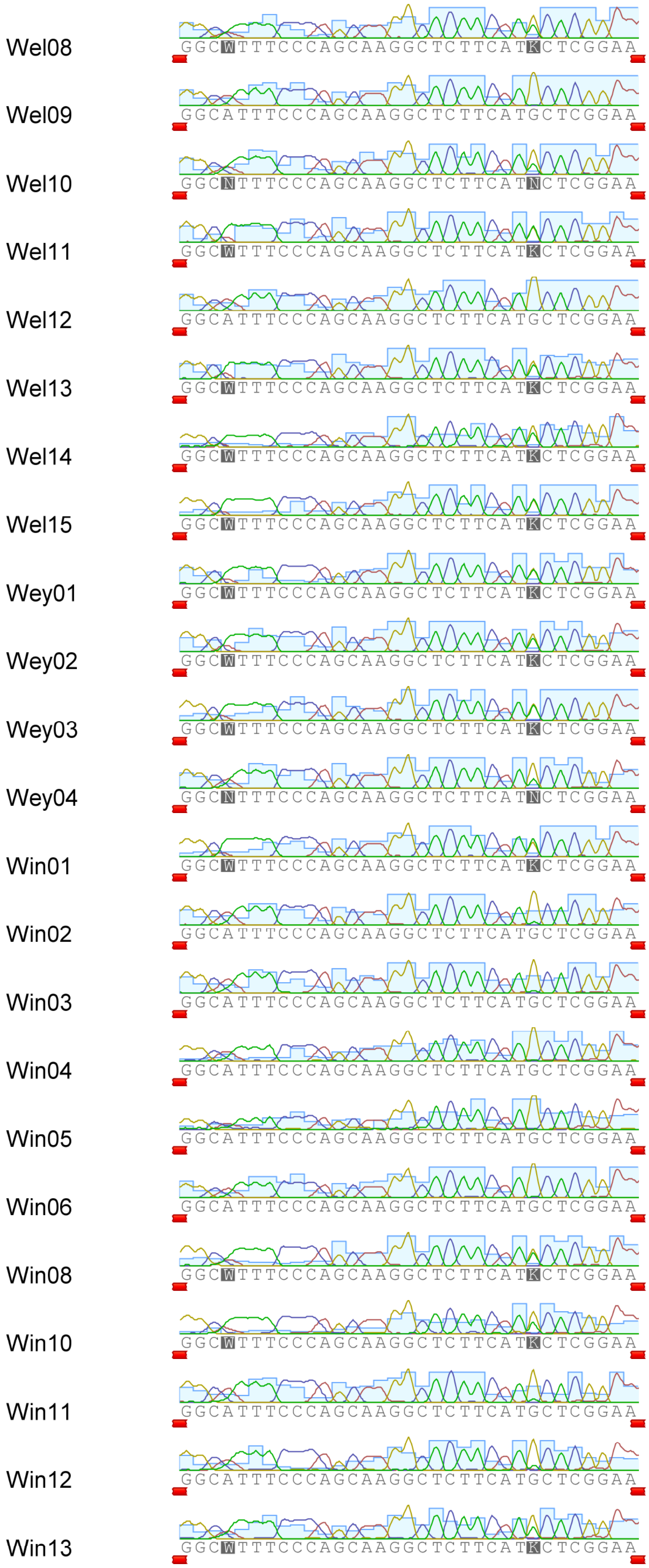
Wel08 GGCWTTTCCCAGCAAGGCTCTTCATKCTCGGAA
Wel09 GGCATTTCCCAGCAAGGCTCTTCATGCTCGGAA
Wel10 GGCNTTTCCCAGCAAGGCTCTTCATNCTCGGAA
Wel11 GGCWTTTCCCAGCAAGGCTCTTCATKCTCGGAA
Wel12 GGCATTTCCCAGCAAGGCTCTTCATGCTCGGAA
Wel13 GGCWTTTCCCAGCAAGGCTCTTCATKCTCGGAA
Wel14 GGCWTTTCCCAGCAAGGCTCTTCATKCTCGGAA
Wel15 GGCWTTTCCCAGCAAGGCTCTTCATKCTCGGAA
Wey01 GGCWTTTCCCAGCAAGGCTCTTCATKCTCGGAA
Wey02 GGCWTTTCCCAGCAAGGCTCTTCATKCTCGGAA
Wey03 GGCWTTTCCCAGCAAGGCTCTTCATKCTCGGAA
Wey04 GGCNTTTCCCAGCAAGGCTCTTCATNCTCGGAA
Win01 GGCWTTTCCCAGCAAGGCTCTTCATKCTCGGAA
Win02 GGCATTTCCCAGCAAGGCTCTTCATGCTCGGAA
Win03 GGCATTTCCCAGCAAGGCTCTTCATGCTCGGAA
Win04 GGCATTTCCCAGCAAGGCTCTTCATGCTCGGAA
Win05 GGCATTTCCCAGCAAGGCTCTTCATGCTCGGAA
Win06 GGCATTTCCCAGCAAGGCTCTTCATGCTCGGAA
Win08 GGCWTTTCCCAGCAAGGCTCTTCATKCTCGGAA
Win10 GGCWTTTCCCAGCAAGGCTCTTCATKCTCGGAA
Win11 GGCATTTCCCAGCAAGGCTCTTCATGCTCGGAA
Win12 GGCATTTCCCAGCAAGGCTCTTCATGCTCGGAA
Win13 GGCWTTTCCCAGCAAGGCTCTTCATKCTCGGAA

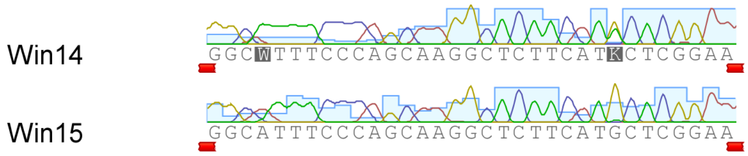
Win14
GGCWTTTCCCAGCAAGGCTCTTCATKCTCGGAA
Win15
GGCATTTCCCAGCAAGGCTCTTCATGCTCGGAA
