# Supplementary material for: Genomic basis for an informed conservation management of Pelophylax water frogs in Luxembourg
Source: Ecol Evol. 2022 Apr 11;12(4):e8810. doi: 10.1002/ece3.8810 (PMC9001158; doi:10.1002/ece3.8810)
Supplement: Supplementary file 3 — Figure S3 [file ECE3-12-e8810-s001.pdf]

Figure S3. Chromatograms of the *MND1* sequences. For each individual, two variable sites (position 4 and 26 in the alignment) were analysed for species identification. All sequences are based on DNA from buccal or skin swabs.

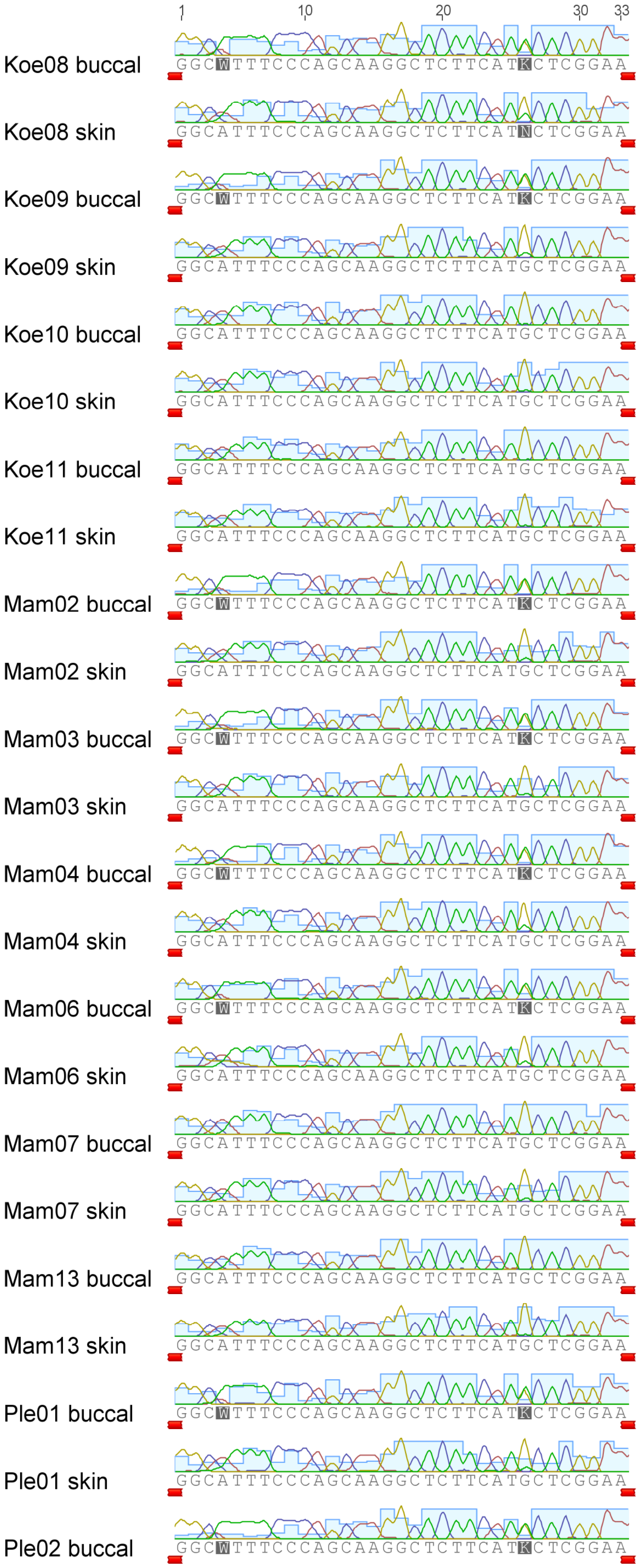
1
10
20
30
33
Koe08 buccal
GGCWTTTCCCAGCAAGGCTCTTCATKCTCGGAA
Koe08 skin
GGCATTTCCCAGCAAGGCTCTTCATNCTCGGAA
Koe09 buccal
GGCWTTTCCCAGCAAGGCTCTTCATKCTCGGAA
Koe09 skin
GGCATTTCCCAGCAAGGCTCTTCATGCTCGGAA
Koe10 buccal
GGCATTTCCCAGCAAGGCTCTTCATGCTCGGAA
Koe10 skin
GGCATTTCCCAGCAAGGCTCTTCATGCTCGGAA
Koe11 buccal
GGCATTTCCCAGCAAGGCTCTTCATGCTCGGAA
Koe11 skin
GGCATTTCCCAGCAAGGCTCTTCATGCTCGGAA
Mam02 buccal
GGCWTTTCCCAGCAAGGCTCTTCATKCTCGGAA
Mam02 skin
GGCATTTCCCAGCAAGGCTCTTCATGCTCGGAA
Mam03 buccal
GGCWTTTCCCAGCAAGGCTCTTCATKCTCGGAA
Mam03 skin
GGCATTTCCCAGCAAGGCTCTTCATGCTCGGAA
Mam04 buccal
GGCWTTTCCCAGCAAGGCTCTTCATKCTCGGAA
Mam04 skin
GGCATTTCCCAGCAAGGCTCTTCATGCTCGGAA
Mam06 buccal
GGCWTTTCCCAGCAAGGCTCTTCATKCTCGGAA
Mam06 skin
GGCATTTCCCAGCAAGGCTCTTCATGCTCGGAA
Mam07 buccal
GGCATTTCCCAGCAAGGCTCTTCATGCTCGGAA
Mam07 skin
GGCATTTCCCAGCAAGGCTCTTCATGCTCGGAA
Mam13 buccal
GGCATTTCCCAGCAAGGCTCTTCATGCTCGGAA
Mam13 skin
GGCATTTCCCAGCAAGGCTCTTCATGCTCGGAA
Ple01 buccal
GGCWTTTCCCAGCAAGGCTCTTCATKCTCGGAA
Ple01 skin
GGCATTTCCCAGCAAGGCTCTTCATGCTCGGAA
Ple02 buccal
GGCWTTTCCCAGCAAGGCTCTTCATKCTCGGAA

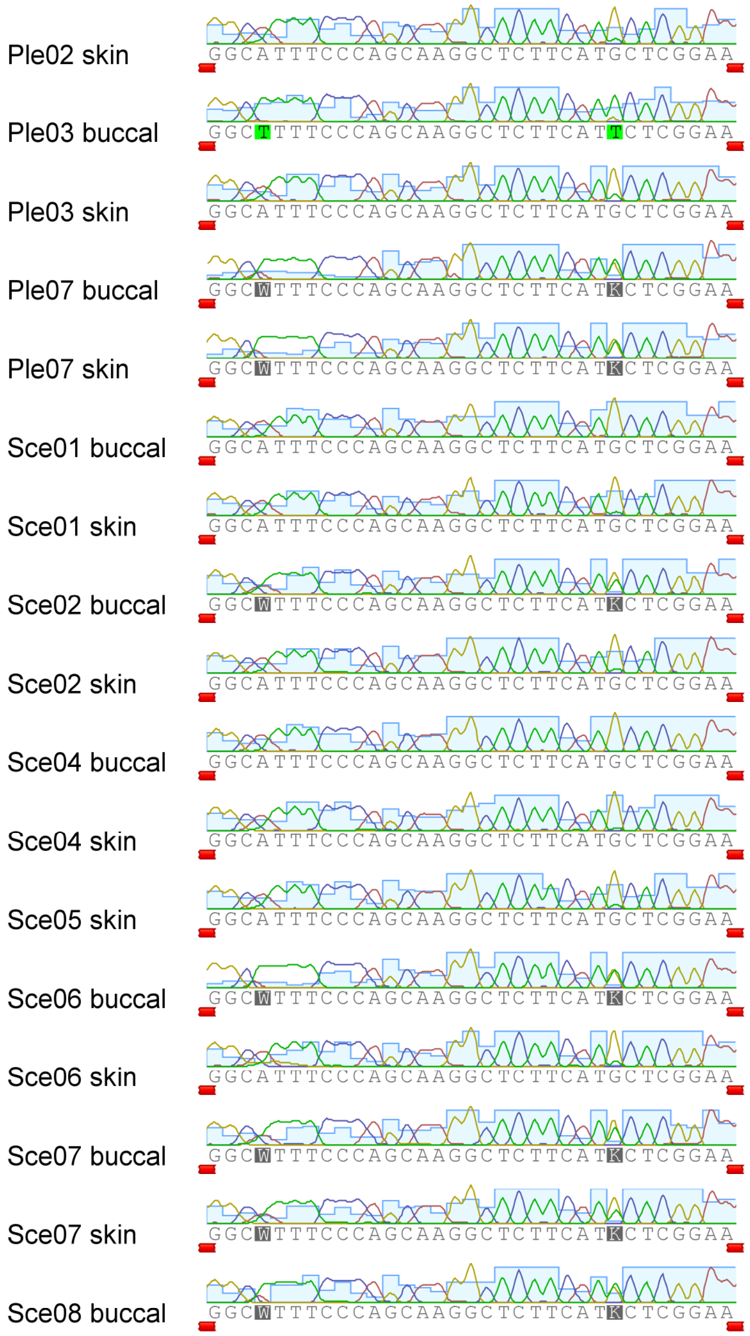
Ple02 skin
GGCATTTCCCAGCAAGGCTCTTCATGCTCGGAA
Ple03 buccal
GGCTTTTCCCAGCAAGGCTCTTCATTCTCGGAA
Ple03 skin
GGCATTTCCCAGCAAGGCTCTTCATGCTCGGAA
Ple07 buccal
GGCWTTTCCCAGCAAGGCTCTTCATKCTCGGAA
Ple07 skin
GGCWTTTCCCAGCAAGGCTCTTCATKCTCGGAA
Sce01 buccal
GGCATTTCCCAGCAAGGCTCTTCATGCTCGGAA
Sce01 skin
GGCATTTCCCAGCAAGGCTCTTCATGCTCGGAA
Sce02 buccal
GGCWTTTCCCAGCAAGGCTCTTCATKCTCGGAA
Sce02 skin
GGCATTTCCCAGCAAGGCTCTTCATGCTCGGAA
Sce04 buccal
GGCATTTCCCAGCAAGGCTCTTCATGCTCGGAA
Sce04 skin
GGCATTTCCCAGCAAGGCTCTTCATGCTCGGAA
Sce05 skin
GGCATTTCCCAGCAAGGCTCTTCATGCTCGGAA
Sce06 buccal
GGCWTTTCCCAGCAAGGCTCTTCATKCTCGGAA
Sce06 skin
GGCATTTCCCAGCAAGGCTCTTCATGCTCGGAA
Sce07 buccal
GGCWTTTCCCAGCAAGGCTCTTCATKCTCGGAA
Sce07 skin
GGCWTTTCCCAGCAAGGCTCTTCATKCTCGGAA
Sce08 buccal
GGCWTTTCCCAGCAAGGCTCTTCATKCTCGGAA
