# Supplementary material for: Genomic basis for an informed conservation management of Pelophylax water frogs in Luxembourg
Source: Ecol Evol. 2022 Apr 11;12(4):e8810. doi: 10.1002/ece3.8810 (PMC9001158; doi:10.1002/ece3.8810)
Supplement: Supplementary file 4 — Table S1 [file ECE3-12-e8810-s007.docx]

Table S1. Information per sample site.

| **Site** | **Village** | **Coordinates** | | **N** | ***P. lessonae*** | ***P.* kl. *esculentus*** | | **RR - Genotype** | **Unknown Species** | |
| --- | --- | --- | --- | --- | --- | --- | --- | --- | --- | --- |
|  |  | **Latitude** | **Longitude** |  |  | **Total** | **LR/LLR/?** |  | **With ddRAD data** | **Without ddRAD data** |
| Abw | Abweiler | 49.537 N | 6.091 E | 15 | 4 | 11 | 7/3/1 | 0 | 0 | 0 |
| Alz | Alzingen | 49.558 N | 6.149 E | 8 | 2 | 6 | 5/0/1 | 0 | 0 | 0 |
| Bas | Basbellain | 50.134 N | 5.988 E | 5 | 4 | 1 | 1/0/0 | 0 | 0 | 0 |
| Bis | Bissen | 49.808 N | 6.051 E | 15 | 11 | 4 | 2/0/2 | 0 | 0 | 0 |
| Fis | Fischbach | 49.758 N | 6.193 E | 10 | 0 | 10 | 9/0/1 | 0 | 0 | 0 |
| Fri | Frisange | 49.509 N | 6.188 E | 5 | 0 | 5 | 4/0/1 | 0 | 0 | 0 |
| Goe | Goedange | 50.143 N | 6.006 E | 7 | 7 | 0 | 0/0/0 | 0 | 0 | 0 |
| Gra | Grass | 49.627 N | 5.896 E | 15 | 1 | 14 | 14/0/0 | 0 | 0 | 0 |
| Har | Harlange | 49.935 N | 5.789 E | 11 | 11 | 0 | 0/0/0 | 0 | 0 | 0 |
| Hol | Hollefels | 49.719 N | 6.061 E | 15 | 8 | 7 | 2/4/1 | 0 | 0 | 0 |
| Hoz | Holzem | 49.603 N | 5.982 E | 15 | 2 | 13 | 10/0/3 | 0 | 0 | 0 |
| Kal | Kalkesbad | 49.800 N | 6.364 E | 15 | 0 | 15 | 14/0/1 | 0 | 0 | 0 |
| Koe | Koerich | 49.683 N | 5.941 E | 15 | 10 | 5 | 5/0/0 | 0 | 0 | 0 |
| Mam | Mamer | 49.631 N | 6.009 E | 15 | 2 | 13 | 11/0/2 | 0 | 0 | 0 |
| Mtz | Mertzig | 49.832 N | 6.032 E | 1 | 1 | 0 | 0/0/0 | 0 | 0 | 0 |
| Nie | Niederfeulen | 49.874 N | 6.046 E | 15 | 0 | 12 | 10/0/2 | 3 | 0 | 0 |
| Noe | Noertzange | 49.512 N | 6.049 E | 15 | 0 | 14 | 0/13/1 | 1 | 0 | 0 |
| Pep | Peppange | 49.520 N | 6.118 E | 16 | 4 | 11 | 1/8/2 | 0 | 1 | 0 |
| Pet | Petrange | 49.558 N | 5.861 E | 6 | 0 | 4 | 2/2/0 | 2 | 0 | 0 |
| Ple | Pleitrange | 49.593 N | 6.279 E | 10 | 0 | 9 | 7/0/2 | 1 | 0 | 0 |
| Red | Redange | 49.760 N | 5.879 E | 15 | 11 | 4 | 4/0/0 | 0 | 0 | 0 |
| Sae | Saeul | 49.733 N | 5.982 E | 15 | 2 | 13 | 11/0/2 | 0 | 0 | 0 |
| San | Sandweiler | 49.605 N | 6.222 E | 15 | 10 | 5 | 4/0/1 | 0 | 0 | 0 |
| Sav | Savelbur | 49.807 N | 6.237 E | 15 | 14 | 0 | 0/0/0 | 0 | 0 | 1 |
| Sca | Schandel | 49.799 N | 5.966 E | 15 | 7 | 8 | 7/0/1 | 0 | 0 | 0 |
| Sce | Scheidgen | 49.772 N | 6.371 E | 8 | 2 | 6 | 5/0/1 | 0 | 0 | 0 |
| Sci | Schieren | 49.835 N | 6.112 E | 5 | 2 | 3 | 3/0/0 | 0 | 0 | 0 |
| Sco | Schouweiler | 49.582 N | 5.941 E | 15 | 2 | 12 | 12/0/0 | 0 | 0 | 1 |
| Scu | Schuttrange | 49.622 N | 6.290 E | 1 | 0 | 1 | 1/0/0 | 0 | 0 | 0 |
| Sur | Surre | 49.903 N | 5.780 E | 15 | 7 | 7 | 6/0/1 | 0 | 1 | 0 |
| Wel | Welfrange | 49.538 N | 6.307 E | 15 | 6 | 9 | 9/0/0 | 0 | 0 | 0 |
| Wey | Weyer | 49.724 N | 6.197 E | 4 | 0 | 4 | 3/1/0 | 0 | 0 | 0 |
| Win | Wintrange | 49.506 N | 6.350 E | 15 | 9 | 6 | 4/0/2 | 0 | 0 | 0 |
| **Total** |  |  |  | **382** | **139** | **232** | **173/31/28** | **7** | **2** | **2** |
