# Supplementary material for: Genomic basis for an informed conservation management of Pelophylax water frogs in Luxembourg
Source: Ecol Evol. 2022 Apr 11;12(4):e8810. doi: 10.1002/ece3.8810 (PMC9001158; doi:10.1002/ece3.8810)
Supplement: Supplementary file 8 — Text S1 [file ECE3-12-e8810-s008.pdf]

Text S1 - DNA extraction protocol following Richardson et al. (2001)

1. Starting material
    a. Toe fragment: Add tissue to a 1.5 µl tube with 250 µl Digsol buffer and 10 µl Proteinase K (10 mg/ml)
    b. Swab stored in ethanol: Add swab to a 1.5 µl tube and evaporate ethanol at 55°C; Add 250 µl Digsol buffer and 10 µl Proteinase K (10 mg/ml) to swab
    c. Swab stored without ethanol: Add swab to a 1.5 µl tube with 250 µl Digsol buffer and 10 µl Proteinase K (10 mg/ml) to swab
2. Vortex the samples and incubate at 37°C overnight / 55°C for 3 h with occasional shaking
3. Add 300 µl of 4M ammonium acetate to the digestion
4. Incubate for 15 min. Vortex the tubes several times during the incubation
5. Centrifuge the tubes for 10 min at 14.000 rpm
6. Add supernatant to new 1.5 µl tubes
7. Centrifuge the tubes for 5 min at 14.000 rpm
8. Add supernatant to new 1.5 µl tubes
9. Add 1 ml 96 % ethanol to the supernatant
10. Centrifuge the tubes for 10 min at 14.000 rpm
11. Remove supernatant and add 500 µl 70 % ethanol to the pellet
12. Centrifuge the tubes for 5 min at 14.000 rpm
13. Remove supernatant. Dry pellet by either air-drying for 30 – 60 min or by incubating the tubes for 10 – 15 min at 55°C and 400 rpm
14. Dissolve the pellet with 50 µl Temin. Incubate the tube either for 30 min at 55°C or overnight at room temperature

Digsol buffer

Ingredient	Final concentration	Stock concentration	Amount for 200 ml buffer
**EDTA**	20 mM	0.5 M, pH 8	8 ml
**NaCl**	120 mM	solid	1,37 g
**Tris**	50 mM	1 M, pH 8	10 ml
**Autoclaved Water**			172 ml

Autoclave the buffer and cool down. Add 10 ml 20 % SDS-solution.

Ammonium acetate (4M)

Ingredient	Final concentration	Stock concentration	Amount for 200 ml buffer
**Ammonium acetate**	4 M	solid	61,66 g
**Autoclaved Water**			Add to 200 ml

Autoclave the buffer and cool down.
