# Supplementary material for: Genomic basis for an informed conservation management of Pelophylax water frogs in Luxembourg
Source: Ecol Evol. 2022 Apr 11;12(4):e8810. doi: 10.1002/ece3.8810 (PMC9001158; doi:10.1002/ece3.8810)
Supplement: Supplementary file 9 — Text S2 [file ECE3-12-e8810-s009.pdf]

Text S2 - ddRAD Protocol following Schweyen et al. (2014)

RNAse Digestion

- Digest DNA mit 1 µl (10 µg/µl) RNAse A für 30 min at 37°C
- Purify sample with NucleoSpin Gel und PCR Clean-up Kit (Macherey-Nagel), eluate two times with 25 µl elution buffer (after 1 min incubation at room temperature and after 5 min incubation at 70°C).

DNA quantification

- Use 2 µl DNA per sample
- Quantify with Xpose (Trinean) and Slide 40 (A260 dsDNA)

Double digest

	Stock	Volume for 50 µl
**CutSmart buffer**	10 x	5 µl
**MspI**	20.000 U/ml	1 µl
**SbfI-HF**	20.000 U/ml	1 µl
**DNA**		200 – 600 ng
**Water**		Fill up to 50 µl

- Incubate for 1 h at 37°C
- Purify sample with NucleoSpin Gel und PCR Clean-up Kit (Macherey-Nagel), eluate two times with 16 µl elution buffer (after 1 min incubation at room temperature and after 5 min incubation at 70°C).

Ligation

	Stock	Volume for 50 µl
**T4 ligase buffer**	10 x	5 µl
**Sample**		30 µl
**P5 adapter**	1 µM	
**P7 adapter**	10 µM	
**T4 ligase**	2.000.000 U/ml	0.5 µl
**Water**		Fill up to 50 µl

- Incubate for 2 h at room temperature
- Purify sample with NucleoSpin Gel und PCR Clean-up Kit (Macherey-Nagel), eluate two times with 26 µl elution buffer (after 1 min incubation at room temperature and after 5 min incubation at 70°C).

Size selection I

- Preparation
  - Prepare 180 µl 85 % ethanol fresh per sample
  - Shake SPRIselect bottle until the beads are dissolved
- Off the magnet
  - Add 47 µl sample to 47 µl SPRIselect in PCR stripes or 96-well plates
  - Mix by pipetting 10x
  - Incubate for 1 min
- At the magnet
  - Wait until the beads have sedimented
  - Remove supernatant
  - Add 180 µl 85 % Ethanol to the beads
  - Incubate for 30 sec
  - Remove supernatant
  - Dry beads by incubating at room temperature for 5 – 10 min or by using a vacuum centrifuge for 1 – 3 min
- Off the magnet
  - Add 30 µl water or elution buffer (NucleoSpin Gel und PCR Clean-up Kit) to the beads
  - Mix by pipetting 10x
  - Incubate for 1 min
- At the magnet
  - Wait until the beads have sedimented
  - Add supernatant to a new tube

PCR amplification

	Stock	Volume for 50 µl
**Q5 buffer**	5 x	10 µl
**dNTPs**	10 mM	1 µl
**P5 primer**	10 µM	5 µl
**P7 primer**	10 µM	5 µl
**Q5 HF polymerase**	2.000 U/ml	0,5 µl
**DNA**		10 µl
**Water**		18,5 µl

	Zeit	Temperature	
**Initial denaturation**	30 sec	98°C	
**Denaturation**	10 sec	98°C	
**Annealing**	30 sec	65°C	14 Cycle
**Elongation**	30 sec	72°C	
**Finale Elongation**	5 min	72°C	
**Final phase**	Hold	10°C	

PCR purification

- Preparation
    - Prepare 400 µl 70 % ethanol fresh per sample
    - Shake Ampure XP bottle until the beads are dissolved
- Off the magnet
    - Add 47 µl sample to 84.6 µl Ampure XP beads in PCR stripes or 96-well plates (Relation of beads : PCR Product = 1.8)
    - Mix by pipetting 10x
    - Incubate for 5 min
- Add the magnet
    - Wait until the beads have sedimented
    - Remove supernatant
    - Add 200 µl 70 % ethanol to the beads
    - Incubate for 30 sec
    - Remove supernatant
    - Add 200 µl 70 % ethanol to the beads
    - Incubate for 30 sec
    - Remove supernatant
    - Dry beads by incubating at room temperature for 5 – 10 min or by using a vacuum centrifuge for 1 – 3 min
- Off the magnet
    - Add 52 µl water to the beads
    - Mix by pipetting 10x
    - Incubate for 1 min
- Add the magnet
    - Wait until the beads have sedimented
    - Add supernatant to a new tube

Size selection II

- Preparation
  - Prepare 180 µl 85 % ethanol fresh per sample
  - Shake SPRIselect bottle until the beads are dissolved
- Off the magnet
  - Add 50 µl sample to 35 µl SPRIselect in PCR stripes or 96-well plates
  - Mix by pipetting 10x
  - Incubate for 1 min
- At the magnet
  - Wait until the beads have sedimented
  - Add supernatant to a new PCR stripes or 96-well plates
- Off the magnet
  - Add 7.5 µl SPRIselect to the supernatant
  - Mix by pipetting 10x
  - Incubate for 1 min
- At the magnet
  - Wait until the beads have sedimented
  - Remove supernatant
  - Add 180 µl 85 % Ethanol to the beads
  - Incubate for 30 sec
  - Remove supernatant
  - Dry beads by incubating at room temperature for 5 – 10 min or by using a vacuum centrifuge for 1 – 3 min
- Off the magnet
  - Add 22 µl water or elution buffer (NucleoSpin Gel und PCR Clean-up Kit) to the beads
  - Mix by pipetting 10x
  - Incubate for 1 min
- At the magnet
  - Wait until the beads have sedimented
  - Add supernatant to a new tube

Pooling and final purification of the library

- Visualize 2 µl on a 2 % agarose gel and quantify band intensity
- Pool samples by using 3 – 20 µl, depending on band intensity
- Reduce sample volume using a NucleoSpin Gel und PCR Clean-up Kit, eluate two times with 30 µl elution buffer (after 1 min incubation at room temperature and after 5 min incubation at 70°C).
- Repeat size selection II as described above with 50 µl of the eluate
- Quantify the pool as described in DNA quantification
