# Supplementary material for: Genomic basis for an informed conservation management of Pelophylax water frogs in Luxembourg
Source: Ecol Evol. 2022 Apr 11;12(4):e8810. doi: 10.1002/ece3.8810 (PMC9001158; doi:10.1002/ece3.8810)
Supplement: Supplementary file 10 — Text S3 [file ECE3-12-e8810-s010.pdf]

Text S3 - ddRAD Primer and Adapter

P5 forward adapter

P5_AF_1	ACACTCTTTCCCTACACGACGCTCTTCCGATCTNNNNNNIICACAGTGCA
P5_AF_2	ACACTCTTTCCCTACACGACGCTCTTCCGATCTANNNNNNIICACAGTGCA
P5_AF_3	ACACTCTTTCCCTACACGACGCTCTTCCGATCTTCNNNNNNIIGTCACTGCA
P5_AF_4	ACACTCTTTCCCTACACGACGCTCTTCCGATCTCGANNNNNNIIGTCACTGCA

P5 reverse adapter

P5_AR_1	CTGTGMMNNNNNNAGATCGGAAGAGCGTCGTGTAGGGAAAGAGTGT
P5_AR_2	CTGTGMMNNNNNNTAGATCGGAAGAGCGTCGTGTAGGGAAAGAGTGT
P5_AR_3	GTGACMMNNNNNNGAAGATCGGAAGAGCGTCGTGTAGGGAAAGAGTGT
P5_AR_4	GTGACMMNNNNNNTCGAGATCGGAAGAGCGTCGTGTAGGGAAAGAGTGT

P7 forward adapter

P7_AF_1	GTGACTGGAGTTCAGACGTGTGCTCTTCCGATCT

P7 reverse adapter

P7_AR_1	CGAGATCGGAAGAGCGAGAACAA

P5 primer

P5_Pr_UD01
AATGATACGGCGACCACCGAGATCTACACAGCGCTAGACACTCTTTCCCTACACGACGCTCTTCCGATCT
P5_Pr_UD02
AATGATACGGCGACCACCGAGATCTACACGATATCGAACACTCTTTCCCTACACGACGCTCTTCCGATCT
P5_Pr_UD03
AATGATACGGCGACCACCGAGATCTACACCGCAGACGACACTCTTTCCCTACACGACGCTCTTCCGATCT
P5_Pr_UD04
AATGATACGGCGACCACCGAGATCTACACTATGAGTAACACTCTTTCCCTACACGACGCTCTTCCGATCT
P5_Pr_UD05
AATGATACGGCGACCACCGAGATCTACACAGGTGCGTACACTCTTTCCCTACACGACGCTCTTCCGATCT
P5_Pr_UD06
AATGATACGGCGACCACCGAGATCTACACGAACATACACACTCTTTCCCTACACGACGCTCTTCCGATCT
P5_Pr_UD07
AATGATACGGCGACCACCGAGATCTACACACATAGCGACACTCTTTCCCTACACGACGCTCTTCCGATCT
P5_Pr_UD08
AATGATACGGCGACCACCGAGATCTACACGTGCGATAACACTCTTTCCCTACACGACGCTCTTCCGATCT
P5_Pr_UD11
AATGATACGGCGACCACCGAGATCTACACCGCGGTTCACACTCTTTCCCTACACGACGCTCTTCCGATCT
P5_Pr_UD12
AATGATACGGCGACCACCGAGATCTACACTATAACCTACACTCTTTCCCTACACGACGCTCTTCCGATCT

P7 primer

P7_Pr_UD01	CAAGCAGAAGACGGCATACGAGATAACCGCGGGTGACTGGAGTTCAGACGTGTGC
P7_Pr_UD02	CAAGCAGAAGACGGCATACGAGATGGTTATAAGTGACTGGAGTTCAGACGTGTGC
P7_Pr_UD03	CAAGCAGAAGACGGCATACGAGATCCAAGTCCGTGACTGGAGTTCAGACGTGTGC
P7_Pr_UD04	CAAGCAGAAGACGGCATACGAGATTTGGACTTGTGACTGGAGTTCAGACGTGTGC
P7_Pr_UD05	CAAGCAGAAGACGGCATACGAGATCAGTGGATGTGACTGGAGTTCAGACGTGTGC
P7_Pr_UD06	CAAGCAGAAGACGGCATACGAGATTGACAAGCGTGACTGGAGTTCAGACGTGTGC
P7_Pr_UD07	CAAGCAGAAGACGGCATACGAGATCTAGCTTGGTGACTGGAGTTCAGACGTGTGC
P7_Pr_UD08	CAAGCAGAAGACGGCATACGAGATTCGATCCAGTGACTGGAGTTCAGACGTGTGC
P7_Pr_UD11	CAAGCAGAAGACGGCATACGAGATAGTAGAGAGTGACTGGAGTTCAGACGTGTGC
P7_Pr_UD12	CAAGCAGAAGACGGCATACGAGATGACGAGAGGTGACTGGAGTTCAGACGTGTGC
P7_Pr_UD15	CAAGCAGAAGACGGCATACGAGATCTTAAGCCGTGACTGGAGTTCAGACGTGTGC
P7_Pr_UD16	CAAGCAGAAGACGGCATACGAGATTCCGGATTGTGACTGGAGTTCAGACGTGTGC
