# Supplementary material for: Genomic basis for an informed conservation management of Pelophylax water frogs in Luxembourg
Source: Ecol Evol. 2022 Apr 11;12(4):e8810. doi: 10.1002/ece3.8810 (PMC9001158; doi:10.1002/ece3.8810)
Supplement: Supplementary file 11 — Text S4 [file ECE3-12-e8810-s002.pdf]

Text S4 – Protocol for single markers

***MND1* – modified after Tecker et al. (2017)**
Forward primer: GGA CGA TGC CAT GGT AGA TT
Reverse primer: CCA GGG TCT CCA GCT TTC TT

	Stock	Volume for 8 µl
**Qiagen Multiplex PCR mastermix**	2 x	4 µl
**Forward primer**	2 µM	1 µl
**Reverse primer**	2 µM	1 µl
**DNA**		1 µl
**Water**		1 µl

	Time	Temperature	
**Initial denaturation**	5 min	95°C	
**Denaturation**	30 sec	95°C	
**Annealing**	30 sec	56°C	35 Cycle
**Elongation**	30 sec	72°C	
**Final elongation**	5 min	72°C	
**Final phase**	Hold	10°C	

***ND1* – modified after Holsbeek et al. (2008)**
Forward primer: AAA CTA TTT AYY AAA GAR CC
Reverse primer: GGG TAT GAN GCT CGN ACT CA

	Stock	Volume for 5 µl
**Qiagen Multiplex PCR mastermix**	2 x	2.5 µl
**Forward primer**	10 µM	0.25 µl
**Reverse primer**	10 µM	0.25 µl
**DNA**		0.5 µl
**Water**		1.5 µl

	Time	Temperature	
**Initial denaturation**	5 min	95°C	
**Denaturation**	30 sec	95°C	
**Annealing**	30 sec	48°C	35 Cycle
**Elongation**	45 sec	72°C	
**Final elongation**	7 min	72°C	
**Final phase**	Hold	10°C	
